# Cell density quantification of high resolution Nissl images of the juvenile rat brain

**DOI:** 10.3389/fnana.2024.1463632

**Published:** 2024-12-18

**Authors:** Julie Meystre, Jean Jacquemier, Olivier Burri, Csaba Zsolnai, Nicolas Frank, João Prado Vieira, Ying Shi, Rodrigo Perin, Daniel Keller, Henry Markram

**Affiliations:** ^1^Laboratory of Neural Microcircuitry, École Polytechnique Fédérale de Lausanne, Lausanne, Switzerland; ^2^Blue Brain Project, École Polytechnique Fédérale de Lausanne, Genève, Switzerland; ^3^Bioimaging and Optics Platform, École Polytechnique Fédérale de Lausanne, Lausanne, Switzerland

**Keywords:** rodent, somatosensory hindlimb, cell density, stereology, cortical layering, Cellpose, machine learning

## Abstract

Nissl histology underpins our understanding of brain anatomy and architecture. Despite its importance, no high-resolution datasets are currently available in the literature for 14-day-old rats. To remedy this issue and demonstrate the utility of such a dataset, we have acquired over 2000 high-resolution images (0.346 μm per pixel) from eight juvenile rat brains stained with cresyl violet. To analyze this dataset, we developed a semi-automated pipeline using open-source software to perform cell density quantification in the primary somatosensory hindlimb (S1HL) cortical column. In addition, we performed cortical layer annotations both manually and using a machine learning model to expand the number of annotated samples. After training the model, we applied it to 262 images of the S1HL, retroactively assigning segmented cells to specific cortical layers, enabling cell density quantification per layer rather than just for entire brain regions. The pipeline improved the efficiency and reliability of cell density quantification while accurately assigning cortical layer boundaries. Furthermore, the method is adaptable to different brain regions and cell morphologies. The full dataset, annotations, and analysis tools are made publicly available for further research and applications.

## 1 Introduction

Cell density and brain cytoarchitecture are important for many reasons. The spatial arrangement and density of specific cell types are essential for the function of neuronal microcircuits (Neurohr and Amon, 2020). Proper characterization of cell densities facilitates investigation of pathologies such as neurodegenerative diseases that result in the loss of selective cell populations. Cell density and layer information are also useful for construction of detailed cortical microcircuit models (Markram et al., 2015), though quantitative literature remains sparse (Keller et al., 2018). In this study, we used postnatal age 14 days (P14) animals to compare the data with previous electrophysiological studies. Electrophysiology recordings on P14 animals are easier to perform as juvenile cells are less myelinated.

The Nissl stain is widely used in neuroscience to count the total number of cells, yet data for the juvenile rat Wistar Han at age 14 days is lacking. Our review of available rat atlas revealed no existing resources for this age group. Additionally, no adult-age atlases provide sufficient image resolution or magnification to enable accurate cell counting and delineation of cortical layer boundaries in the specific region of interest (S1HL). While several adult rat atlases exist for different strains, including the Wistar Han (Paxinos and Watson, 2014; Blixhavn et al., 2023; Johnson et al., 2012), the Sprague Dawley (Papp et al., 2014) and the Fischer 344 (Goerzen et al., 2020), none offer adequate histological data for the Wistar Han strain.

For adult Wistar Han rats, histological data is limited to the reference atlas by Paxinos and Watson (2014) and the Nissl-Thionine dataset by Blixhavn et al. (2023). However, the image resolution in Paxinos and Watson (2014) is unspecified, the dataset is not publicly available, and the images are presented in PDF format, which is not suitable for cell counting. Although the Blixhavn et al. (2023) atlas includes higher-resolution images from a single adult animal, the publicly available files appear to be down-sampled, further limiting their utility.

In contrast, the mouse atlases from the Allen Institute for Brain Science (2004) cover both juvenile and adult (P56) ages. However, these atlases also present limitations: the image resolution is three times lower than our study and thus insufficient for reliable cell counting and the images are not consecutive, with a 200 μ*m* gap between samples. Moreover, while these atlases define brain regions, they do not provide cell counts, and most datasets are derived from a single animal.

The cerebral cortex is known for its distinct cytoarchitecture, with variations in the size and subdivisions of its cortical layers across different brain areas and species (Palomero-Gallagher and Zilles, 2019). The primary somatosensory hind limb area (S1HL) exhibits a characteristic six-layer structure, including a well-developed layer IV, comparable to that of primates (García-Cabezas et al., 2022; Briggs, 2010). Accurate quantification of cell densities in these cortical layers is essential for understanding cortical organization.

Manual stereology remains the most rigorous method for cell density quantification, but it is both time-consuming and susceptible to observer bias, as subjective differences in cell counting can lead to inconsistencies (West, 1999). From a standpoint of consistency and efficiency, automatic or semi-automatic methods represent a significant improvement over manual approaches.

In this study, we compiled a robust dataset of eight rodent brains stained with cresyl violet, a conventional Nissl stain commonly used for visualizing cell bodies via bright-field microscopy, which provides an accurate estimate of total cell numbers (García-Cabezas et al., 2016; Gurr, 1960; Nestor, 2008; Warr et al., 1981). To streamline the analysis, we enhanced an open-source cell detector for improved segmentation accuracy and developed a semi-automated pipeline for cell segmentation, stereological exclusion, and cell density analysis. These advancements significantly reduced the time required for traditionally laborious tasks.

Machine learning (ML) methods have been previously employed for layer discrimination in the isocortex (Li et al., 2019; Štajduhar et al., 2023; Wagstyl et al., 2020). Building on these approaches, we developed a pipeline that uses similar ML methods to provide a detailed laminar description of the somatosensory cortex in juvenile rats. This method allows for the retroactive assignment of segmented cells to specific layers, enabling the quantification of cell densities within individual layers, not just across broader brain regions.

By utilizing the same strain and age groupe as previously published studies (Keller et al., 2019), our dataset can be directly compared and incorporated into larger studies. Although the pipeline was primarily developed for the analysis of the somatosensory cortex, we tested it on another cortical region.

To our knowledge, this is the first time such a large dataset has been made publicly available, even though it does not qualify as an atlas due to the slicing angle used. Additionally, this is the first instance in which a complete pipeline—from histology processing to cortical layer identification—has been made available to the public. Finally, this works represents the first application of ML techniques to define cortical layer boundaries in the somatosensory cortex of the rat.

## 2 Materials and methods

### 2.1 Animals

All animal procedures were approved by the Veterinary Authorities and the Cantonal Commission for Animal Experimentation of the Canton of Vaud, according to the Swiss animal protection laws, under authorization number VD3516. The animals originated from the same provider and from three different litters. Each litter was processed individually on different experimental days.

### 2.2 Sample preparation

On postnatal day fourteen, rats were transferred to the experimental room in the morning to acclimate. The described procedure was conducted within a consistent 3-h window of the day (09:00–12:00). Initially, the rats were deeply anesthetized using pentobarbital (intraperitoneal dose of 150 mg/kg; concentration of 150 mg/mL). This was succeeded by transcardial perfusion with ice cold 0.1 M phosphate buffer (PB; pH 7.4), followed by cold 4% paraformaldehyde (PFA) in 0.1 M PB. Subsequently, the brain was carefully removed from the skull, post fixed at 4°C in 4% PFA overnight, and then rinsed in 0.1 M PB. The brains underwent a sequential storage process: first in a 15% sucrose solution (in 0.1 M PB) at 4°C for ~24 h, followed by a 30% sucrose solution at 4°C for an additional 24 h. The hemispheres were carefully divided along the midline, after which both right and left hemispheres were precisely sliced sagittally using a cryostat (Leica, VT-1200S) at 50 μ*m* employing an approximate angle rotation of 4 ± 1 degrees along the anterior-posterior axis to optimize alignment with apical dendrites. These brain slices were stored in a cryoprotectant solution (30% v/v ethylene glycol; 30% m/v sucrose in 0.1 M PB) at –20°C, preserving them until cytochemistry assays were executed (within a maximum of two weeks from extraction to cytochemistry).

Brain slices were stained using cresyl violet, a stain specifically targeting cell bodies, including the endoplasmic reticulum, also known as Nissl substance or Nissl bodies. Free-floating sections of 50 μ*m* thickness were transferred from cryoprotectant into 0.1 M PB to thaw and eliminate any cryoprotectant remnants. Subsequently, they were transferred into 0.01 M PB to minimize salt residues before being meticulously mounted onto SuperFrost©glass slides (Thermo Fisher Scientific Inc., Gerhard Menzel B.V. and Co. KG, GE). This mounting was carried out while considering the brain's orientation relative to the midline, from its external to internal regions. Slide-mounted sections were processed using an automated slide stainer Tissue-Tek^®^ Prisma Plus (Sakura Finetek-Europe, NL). These sections were incubated for 6 min at room temperature (RT = 20°C) in a 0.5% cresyl violet solution in water (with pH adjusted to 2.85 using acetic acid), followed by a brief wash in tap water. The sections underwent dehydration through a series of ethanol concentrations (70, 70, 96, 100, 100%) with each step lasting one minute at RT. Subsequently cleared with two steps of xylene for one minute each at RT, and the sections were mounted using Pertex (Sakura Finetek-Europe, NL) before being cover-slipped using the automated glass coverslipper Tissue-Tek^®^ Glas™ g2 (Sakura Finetek-Europe, NL). A meticulous assessment of the coloration was conducted and if the staining appeared faint, a repeat staining procedure was carried out.

Stained slides were scanned using an automated slide scanner (Olympus, VS120-L100, GER) equipped with a UPLSAPO 20x/0.75 air objective (Olympus, GER) and a Pike F505 Color camera leading to a pixel size of 0.346 μ*m*/pixel. This is three times higher than commonly used atlases (Allen Institute for Brain Science, 2004; Paxinos and Watson, 2014) (see also Table 1). Each brain slice was entirely scanned. Subsequently, the digital images obtained were meticulously organized and subjected to analysis using the open-source software QuPath v0.3.2 (Bankhead et al., 2017). The tasks of cell segmentation training, cell density analysis, and ML were analyzed employing tools from the Python programming language. We used an imaging pixel size of 0.346 × 0.346 μ*m* on the whole dataset.

**Table 1:** Datasets comparison.

**Study**	**Current Meystre et al. (2024)**	**Paxinos and Watson (“PandW”) 7th Ed. 2014**	**Blixhavn et al. (2023)**	**Allen Brain Institute (Juvenile)**	**Allen Brain Institute (Adult)**	**Johnson et al. (2012)**	**Papp et al. (2014)**	**Goerzen et al. (2020)**
**Animal species and strain**	Rat Wistar Han	Rat Wistar Han	Rat Wistar Han	Mouse C57BL/6J	Mouse C57BL/6J	Rat Wistar Han	Rat Sprague Dawley	Rat Fischer 344
**Animal sex**	Male	Male	Not specified^***^	Male	Male	Male	Male	Male, Female
**Animal age**	Juvenile (14 days)	Adult	Adult	Juvenile (14 days)	Adult (56 days)	Adult (80 days)	Adult	Adult (130 ± 7 days)
**Animal weight**	Mean 36.7 grams	270 grams	Not specified^*^	>6 grams	18.8–26.4 grams	250 grams	397.6 grams	282 ± 60 grams
**Sample size**	*N*_animal_ = 8; *N*_S1HL_ = 199	*N*_animal_ = 1; *N*_S1HL_ = 4	*N*_animal_ = 1	*N*_animal_ = 1	*N*_animal_ = 1; *N*_S1HL_ < 20	*N*_animal_ = 5	*N*_animal_ = 1	*N*_animal_ = 41 (24M, 17F)
**Slice thickness**	50 μm sagittal^*^	40 μm sagittal	40 μm sagittal	25 μm sagittal	25 μm sagittal	N/A	N/A	N/A
**Image interval**	Consecutive sample	250 μm gap between samples	80 μm gap between samples	200 μm gap between samples	200 μm gap between samples	N/A	N/A	N/A
**Resolution**	0.346 × 0.346 μm	Not specified^**^	0.22 × 0.22 μm	0.95 × 0.95 μm	0.95 × 0.95 μm	25 μm^3^	39 μm^3^	60 μm^3^
**Magnification**	20x	Not specified^**^	20x	10x	10x	N/A	N/A	N/A
**Modality**	Histology (Nissl-Cresyl Violet)	Histology (Nissl-Cresyl Violet)	Histology (Nissl-Thionine)	Histology (Nissl-thionin or Nissl-Cresyl Violet)	Histology (Nissl-thionin)	Histology, magnetic resonance (MR) and 3D diffusion tensor images (DTI)	Diffusion magnetic resonance images	Magnetic resonance (MR)
**Brain region**	Whole brain	Whole brain	Whole brain	Whole brain	Whole brain	Whole brain	Whole brain	Whole brain
**Label method**	Manual delineation using P/W atlas information	Pencil drawings	Images registered to the WHS atlas	Alignment possible with ARA atlases	Systematic conversion of traditional drawings to digital annotation database	Aligned with the P/W atlas	Semi-automatic (SNAP) and manual segmentation on MR	Stepwise manual segmentation based on T2 contrast on MR template, in conjunction with P/W atlas
**Number of Segmented structures**	1	Over 1,000 structures identified	Same as WHS	0	71	20	118	71
**Data format**	.tiff	.pdf	.tiff	.jpg	.jpg	Not specified	NIfTI	MINC and NIfTI
**Purpose of the study**	Machine learning for histological annotation and quantification of cortical layers	P/W atlas:	A Timm-Nissl multiplane microscopic atlas of rat brain zincergic terminal fields and metal-containing glia	Developing mouse brain atlas	Genome-wide atlas of gene expression in the adult mouse brain	A Multidimensional Magnetic Resonance Histology Atlas of the Wistar Rat Brain	WHS atlas: Waxholm Space atlas of the Sprague Dawley rat brain	An MRI-Derived Neuroanatomical Atlas of the Fischer 344 Rat Brain
**Reference**	10.3389/fnana.2024.1463632	The Rat Brain in Stereotaxic Coordinates, 7th Edition. Elsevier Academic Press, San Diego	doi.org/10.1038/s41597-023-02012-6	developingmouse. brain-map.org/ static/atlas	doi.org/10.1038/nature05453	10.1016/j.neuroimage.2012.05.041	10.1016/j.neuroimage.2014.04.001	doi.org/10.1038/s41598-020-63965-x
**Note and Caveat**	^*^With 4 ± 1° along the anterior-posterior axis.	^**^For the last edition, the biological sections were imaged for us by the ABI, which has enabled us to reproduce the sections in color.	^***^1973 publication, not available online	Mouse and a lower image resolution	Mouse, adult age, and a lower image resolution	Cell counting not possible	Cell counting not possible	Cell counting not possible
**Study**	**Current Meystre et al. (2024)**	**Paxinos and Watson (“PandW”) 7th Ed. 2014**	**Blixhavn et al. (** **2023** **)**	**Allen Brain Institute (Juvenile)**	**Allen Brain Institute (Adult)**			
**Sample image 100** **×100** **μm**	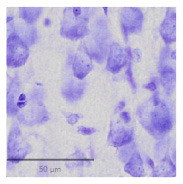	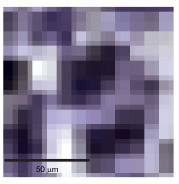	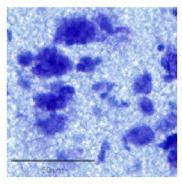	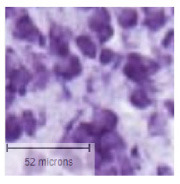	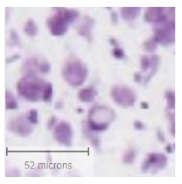			

Detailed information regarding the distinct experimental procedures, duration days within the cryoprotectant, incubation times for staining, the count of slices containing S1HL, the methodology of cell detection have been documented and can be found in Supplementary material.

### 2.3 Cell segmentation

We organized the dataset by structuring the QuPath projects on a per-hemisphere basis (i.e., one project dedicated to each hemisphere, as indicated in Supplementary Table 1). To determine the slice position, the brain distance to midline axis was evaluated by visually matching the closest sagittal section from the Paxinos and Watson (2014) atlas to each sample. This positional data was then integrated into each image as a metadata field called “distance to midline.” Due to the age difference of the specimens between our study and the Paxinos and Watson atlas (respectively, juvenile 40 grams vs. adult 270 grams), the resolution of the atlas plates (50 μ*m* between slices vs. 250 μ*m* between slices for the atlas) and the inherent angle in our slicing procedure (4 ± 1 degrees angle along the anterior-posterior axis vs. 0° for the atlas), a direct comparison of our images with the atlas is not entirely feasible. Because of this slicing angle, the part of the slice spanning from the pia to the hippocampus closely aligns with the published atlas, whereas a linear shift becomes apparent as we move from the hippocampus to the cerebral peduncle. To define the somatosensory cortex anterior border, we used the fact that Layer IV is absent in the motor cortex, whereas it is present in the somatosensory cortex. This helps to determine the start of the S1 region and to exclude slices without a visible Layer IV. To confirm the accuracy of the assigned “distance to midline” values for the acquired images, an expert assessment was conducted.

Each image falling within the S1HL range (spanning from lateral coordinates 1.90–3.18 mm) underwent delineation within the QuPath software. This delineation encompassed defining both the slice contour (SliceContour) and the S1HL brain region (S1HL). Initially, the SliceContour was roughly delineated using a pixel-based thresholder (Resolution: 11 μm/px, RGB average intensity, Gaussian Blur sigma: 4 px, threshold: 211.5) (“Pixel classification—QuPath 0.3.0 documentation,” n.d.). Manual refinement was conducted, with particular focus directed toward obtaining an accurate cortex segmentation. To ensure the integrity of cell density calculations and avoid introducing bias, the cellular aggregates near the pia were excluded from the SliceContour annotation. This exclusion was necessary due to the non-uniform representation of these aggregates in every slice, attributable to cutting artifacts.

We undertook a manual delineation process to define the S1HL brain region and closely replicate the corresponding Paxinos and Watson atlas plate as accurately as possible. The upper boundary of the S1HL region precisely followed the contours outlined in the SliceContour annotation, while the lower boundary was established by the initiation point of the cingulum brain region. In addition, we incorporated some pertinent metadata to each slice, such as a comment section (the “distance to midline” information, and a classification indicating whether the image should be included in the semi-automated analysis: Analyze/True or Analyze/False). This systematic approach was consistently applied across all datasets to ensure the possibility of batch processing.

We opted for using Cellpose, a general deep learning-based segmentation method described to be the most precise in the IoU comparison of different models (Cellpose, Mask R-CNN, Stardist, U-Net3 and U-Net2) (Stringer et al., 2021). Although Cellpose does struggle with handling overlapping cells, this limitation does not impact the primary objective of classifying brain regions based on cell shape and localization features. With Cellpose integrated into QuPath, we were able to generate ground truth cell data directly within the software, which facilitated the training of a custom model. Initially, we selected four images that represent the S1HL region, each sourced from distinct animals, litters, and points along the distance-to-midline axis. On those four images, we randomly positioned three validation and 24 training squares, with each square being (177,16 × 177,16 μ*m*). After applying the pre-trained Cellpose model *cyto*2 (Stringer et al., 2021) to each square, we engaged in manual corrections through visual inspection and curation of the identified cells. In cases where two cells were lightly stained and overlapping, they were counted as one cell. Similarly, if a darker and smaller cell overlapped with a fainter one, we accurately accounted for both entities. It is important to note that the analysis specifically excluded capillaries, endothelial cells, and the cellular aggregates near the pia. Moreover, the ground truth dataset includes not only neuronal cells but a diverse range of cell types including astrocytes, oligodendrocytes, neurons and glial cells.

Following this refinement, we proceeded to train a custom Cellpose model, denoted as *v*1. In the interest of enhancing precision, we imposed constraints on the *v*1 detection phase. This involved considering stacked cells as a single entity instead of two (as in *v*1), and ensuring that the shape was accurate at the edge of the regions. This led to a refined series of ground truth annotations, in order to train a new custom model *v*2. The cell segmentation quality was assessed using three recommended metrics developed by an international consortium (Maier-Hein et al., 2022). Unpaired Welch's *t*-tests were applied to compare results from *cyto*2 and *v*2 mean predictions (see Section 2.7). Then, in order to obtain a reference for the performance of the automated counts, we compared these with cell counts from another human annotator. We selected one of the images used for the segmentation training steps and assigned a second human to correct the segmentation provided by *cyto*2. We then compared this second human annotation (Human 2) and the segmenter final counts *v*2 to the original human ground truth (Human 1). The accuracy between Human 1 and Human 2 or *v*2 were also compared using the Welch's *t*-test.

Once cell segmentation was achieved, reference points (S1HL region of interest (ROI), Outside pia; S1HL contour, S1HL top-right point, S1HL top-left point, S1HL bottom-right point, S1HL bottom-left point) and custom cell measurements (image; name; centroid -*x* in μ*m*; centroid -*y* in μ*m*; cell maximum diameter in μ*m*; cell minimum diameter in μ*m*; the distance to annotation with the outside pia in μ*m*) were extracted from QuPath to proceed with stereological analysis.

### 2.4 Stereology and cell density

The conventional stereological approach to prevent cell overcounting during consecutive slice density estimation involves excluding cells on half of the boundaries of the sampled areas (West, 1999; West and Gundersen, 1990). In our approach, we counted all cells included in the S1HL region without applying such a correction in the -*x* and -*y* directions because all cells were fully counted and not sampled. However, in the -*z* direction, corresponding to the slice thickness and because our datasets contained consecutive slices, such a correction remained necessary. To derive a reasonable -*z* correction factor, a method involving a random assignment of a -*z* coordinate and an appropriate diameter to each cell was employed. The cell diameter was estimated from the six nearest neighboring cells, by computing a mean diameter between these cells. With this information, it is possible to identify cells touching the upper surface of the slice. We compared the -*z* coordinate of the cell added to the half of its diameter to the -*z* coordinate of the upper surface of the slice. The procedure was iterated a hundred times to facilitate bootstrapping, a statistical procedure. We randomly resampled the cell -*z* position to create many simulated samples. Once the stereology correction factor was obtained, cell densities as a function of the percentage of depth inside the somatosensory cortex can be calculated.

Initially, the cell centroid coordinates for the -*x* and -*y* axis as well as the S1HL polygon coordinates were exported from QuPath and the S1HL polygon cartesian point coordinates were converted into a shapely polygon (a two-dimensional feature with a non-*z*ero area enclosed by a linear ring). Cells outside of the polygon were excluded.

A nonlinear grid consisting of 10 columns and 20 rows was generated within the S1HL polygon. Straight vertical lines were calculated using the endpoints derived from the quadrilateral's top and bottom lines, ensuring an even division. The horizontal lines were formed by multiple segments that mimic the shape of the quadrilateral's top and bottom lines, effectively depicting the brain's depth. The resulting split polygons followed the S1HL's top and bottom lines. This nonlinear grid method was constrained to a convex polygon shape. When the method was applied to a concave shape, we realized that split polygons were not constrained anymore in the horizontal axis, resulting in cells from different somatosensory depth being wrongly considered in the same polygon. Slices with a concave S1HL shape were then excluded from further cell density analysis (slices spanning from lateral coordinates of 3.10–3.25 mm). The resulting S1HL size is consequently reduced within lateral coordinates of 1.90–3.05 mm.

For each split polygon, we counted the number of cells and computed its volume by using the theoretical slice thickness of 50 μ*m* (volume in mm^3^). The cell densities were then computed as a function of the percentage of the somatosensory depth. For each of the density computed per depth percentage, the mean values and the standard deviation were calculated (cells/mm^3^).

### 2.5 Cortical layer boundaries

Defining layer boundaries in an image is challenging for humans. To establish precise laminar delineation within our datasets, we used (Palomero-Gallagher and Zilles, 2019) layering properties. The cortical column extends from Layer I to Layer VI and each layer has a different and representative cell population. Layer I contains small soma sizes with round shape and is sparse in cell density; Layer II contains medium soma sizes with triangular soma shapes and is denser than Layer I; Layer III contains a broader range of soma sizes, with a majority of ovoid shape and has a slightly sparser density than Layer II; Layer IV contains a greater number of small soma sizes with similar ovoid shape and has a similar density as Layer II; Layer V contains small, medium and large soma sizes with a lower density compared with Layer IV (Layer Va) and in the transition to Layer VI, the soma sizes increase drastically and become more distinctly pyramidal in shape (large pyramidal cell population; Layer Vb); Layer VIa has an average to dense cell density, with small and medium soma sizes and lacks large pyramidal cells; Layer VIb is sparse, with a majority of small soma oriented horizontally. The white matter clearly defines the bottom of Layer VI.

We manually curated a subset of thirty-eight distributed images, with each hemisphere represented by two to three images covering the entire S1HL volume. The SliceContour and S1HL annotation, as well as the S1HL cell segmentation (*v*2) output were imported from the cell segmentation phase. The manually annotated images were curated to form a dataset that can be used to train and evaluate a model (“layer boundaries expert-classified dataset”). In line with the classification established by Brodmann (1909), we-human identified and categorized the following cortical layers: Layer I, Layer II, Layer III, Layer II/III, Layer IV, Layer V, Layer VIa, Layer VIb. In instances where conditions allowed for a clear visual differentiation between Layer II and Layer III, we ensured their distinct separation (Layer II and Layer III). Otherwise, in cases where visual separation was not feasible, we opted to merge these layers (Layer II/III). For measurement purposes, the annotation “Outside Pia” was added, the cell positions registered and the corresponding result file exported.

Once this baseline expert-classified dataset was defined, a supervised ML approach for cell classification in cortical layers was developed. The goals were to decrease the bias that can occur between human annotators and to improve the annotation throughput. Careful feature selection using the permutation importance algorithm (Breiman, 2001) was carried out to reduce the dimensionality of the data and improve the model's performance. Only features with a positive impact on the predictions were kept. This ML task can be defined as a multiclass classification problem at the cell level (i.e., each cell within an image has to be classified as belonging to one of the seven cortical layers classes: Layer I, Layer II, Layer III, Layer IV, Layer V, Layer VIa or Layer VIb). To train the models, the dataset was split into images for training and others used for testing. Models of varying degrees of complexity (such as image segmentation models) could be employed to solve the task. However, due to the large number of pixels in the sample set [one full image contains roughly 46,000 (width) × 31,000 (height) pixels], employing complex convolutional neural network architectures like U-Net (Ronneberger et al., 2015) would result in significant computational overhead and increase processing times without necessarily yielding improved results. Furthermore, we would lose the rich features gained from QuPath, such as area, length, circularity, solidity of the cells. Therefore, we did not explore the feasibility of using segmentation models, but structured the problem as a cell level classification task instead.

The classification displays an intrinsic spatial relationship as each layer sequentially appears in the slices. We therefore experimented with simpler models such as a K-Nearest Neighbor (KNN) (Cover and Hart, 1967) and a Random Forest (RF) (Breiman, 2001). These models were evaluated according to four metrics: accuracy, precision, recall and F1-score (Fawcett, 2006), using a 10-fold cross-validation (Hastie et al., 2001) method. These metrics provide good insights into the model performance, and facilitate comparison across models. Notably, they are computed by averaging the per-class metrics for each model, ensuring that classes with varying sample sizes contribute equally to the final metric values. We tested the models on two paradigms: one in which Layer II was separated from Layer III (LII and LIII distinguished), and another in which Layer II was merged with Layer III (LII/LIII merged). Once the model was trained, we applied it to naive data and assigned a predicted layer to each cell. For each image, the predicted layer to each cell was registered.

### 2.6 Cell densities and size predictions

With the cells segmented and the predicted layer assigned to each cell, the cell densities and the cell soma sizes as a function of the somatosensory predicted layer can be calculated. We first created the smallest polygon possible containing all the cells of a given predicted layer using the Alpha-Concave hull method (Asaeedi et al., 2017). In this method, the alpha value (α) can be adjusted to tighten or loosen the polygon, thereby integrating points inside or outside the concave hull.

To generalize the method and account for the slight variations in cell density between images, we aimed to find a single α value that could be applied universally. For each α tested, we measured the bounding area surrounding each layer and counted the rejected cells. We selected the value that created a bounding area as close as possible to the actual layer shape without rejecting any cells. This empirical testing approach provided a single α value applicable to all layers, except for Layer I. Due to the much lower cell density in Layer I, the method yielded a smaller α value, corresponding to one-tenth of the value used for the other layers.

The volume of each alpha shape polygon, corresponding to each layer, was computed using the theoretical slice thickness of 50 μ*m*. The number of cells within each alpha shape polygon was counted, and the cell density was subsequently calculated. The cell soma size was determined by utilizing the area (μ*m*^2^) feature of each cell, assuming a perfect circular morphology. From this, a mean diameter was calculated for analysis.

We applied statistical tests to determine if a bimodal Gaussian for to the cell diameter distribution produces statistically different means. We also tested if there are statistically different cell density differences across layers (see statistical methods).

### 2.7 Statistical methods

The cell segmentation quality was assessed using several metrics. The Dice Similarity Coefficient (DSC) is the most widely used counting metric in medical image analysis. It measures the overlap between the prediction and the reference and yields a value between 0, for no overlap, and 1, in the case of full overlap. The Intersection over Union (IoU) metric quantifies the degree of overlap between two objects. As IoU approaches 1, the annotation converges to ground truth. The accuracy, commonly used in classification tasks to assess the overall detection performance (with respect to the ground truth), was calculated as follows:


(1) $$\begin{array}{l} \text{Accuracy}=\frac{\text{True Positives}}{\text{True Positives + False Positives + False Negatives}} \end{array}$$

The advantages and disadvantages of each metric are listed in Stringer et al. (2021)).

Unpaired Welch's *t*-tests were applied to compare results from *cyto*2 and *v*2 mean predictions. Results are plotted and *p*-values are reported with asterisk convention (e.g., *corresponds to *p*-value ≤ 0.1, ***corresponds to *p*-value ≤ 0.001). The unpaired Welch's *t*-test assumes that the means being compared are normally distributed, but does not assume that the samples come from populations with equal variance. The segmentation accuracy between Human 1 and Human 2 or *v*2 was also compared using the Welch's *t*-test.

The distribution of cell diameters followed a bimodal Gaussian distribution. Using scikit-learn's GaussianMixture model, we estimated the parameters of a Gaussian mixture distribution for the cell diameters on an image-by-image basis (228 images). This approach yielded two mean diameters for each layer in each image: one representing the smaller cell population and one for the larger cell population. Next, we calculated the average smaller and larger mean diameters across all images for each layer. To assess whether these two mean diameters significantly differ within each layer, we conducted two-sample t-tests, applying a p-value threshold of 0.05 to evaluate the significance.

To determine the statistical significance of cell density differences across layers, first, cell density for each cortical layer and in each image was calculated. Second, these cell density values were aggregated into seven datasets, each representing a distinct cortical layer. Finally, the Kruskal-Wallis *H*-test using the SciPy kruskal function was performed on the seven datasets, and p-value was calculated to determine the statistical significance of cell density differences across layers. Finally, We used two-sample t-tests corrected for multiple comparisons with the Bonferroni method adjusting the alpha value for the six comparisons (α = 0.05/6 = 0.0083) to assess the difference in cell density between adjacent layers.

We observed a significant improvement in the segmenter accuracy (*cyto*2: 0.2457 ± 0.0596; *v*2: 0.5554 ± 0.0643) and in the overlap between the prediction and the reference (Dice Similarity Coefficient metric, DSC) for *v*2 compared with *cyto*2 (*cyto*2: 0.3908 ± 0.0768; *v*2: 0.7119 ± 0.0542). The intersection over the union (IoU) improvement was below a threshold of *p* = 0.05 and then cannot be considered statistically significant (*cyto*2: 0.6383 ± 0.0677; *v*2: 0.7260 ± 0.0626). A possible explanation for the generalized increase in all metrics can be an improvement in the segmentation task, as *v*2 seemed to be less biased in favor of annotating larger cells than *cyto*2. Although statistical analysis with (N_*Image*_ = 4) was at the low end limit of applicability due to samples size, the cell segmentation improvement allowed us to calculate and refine the rat somatosensory cortical cell density numbers previously published (Keller et al., 2019; Markram et al., 2015).

## 3 Results

### 3.1 Sample preparation

The 8 animals behaved normally and no brain abnormality was noticed at the organ collection. Three had their eyes closed, two had their eyes starting to open, and three had their eyes opened. The animal weights were comprised between 26.8 and 45.6 grams with a mean of 35.48 ± 7.90 grams. Sixteen hemispheres were completely sliced at a thickness of 50 μ*m* and the Nissl staining protocol was applied on 2,077 slices.

During processing, one batch of staining required corrections. Specifically, one dataset needed re-staining due to an acetic acid step that caused a reduction in staining intensity. To rectify this issue, these slides underwent a de-coverslipping process by immersing them in a xylene bath. Subsequently, the cresyl violet staining procedure was repeated. The goal was to restore the precise color pattern and to match the quality and characteristics of the previous successful stainings.

In another dataset, an improper slicing orientation led to the exclusion of a left hemisphere while a distinct right hemisphere was omitted due to the evident hydrocephalic brain anatomy appearance. It is worth noting that the corresponding left hemisphere from the same subject exhibited normal anatomical features.

**Figure 1:**
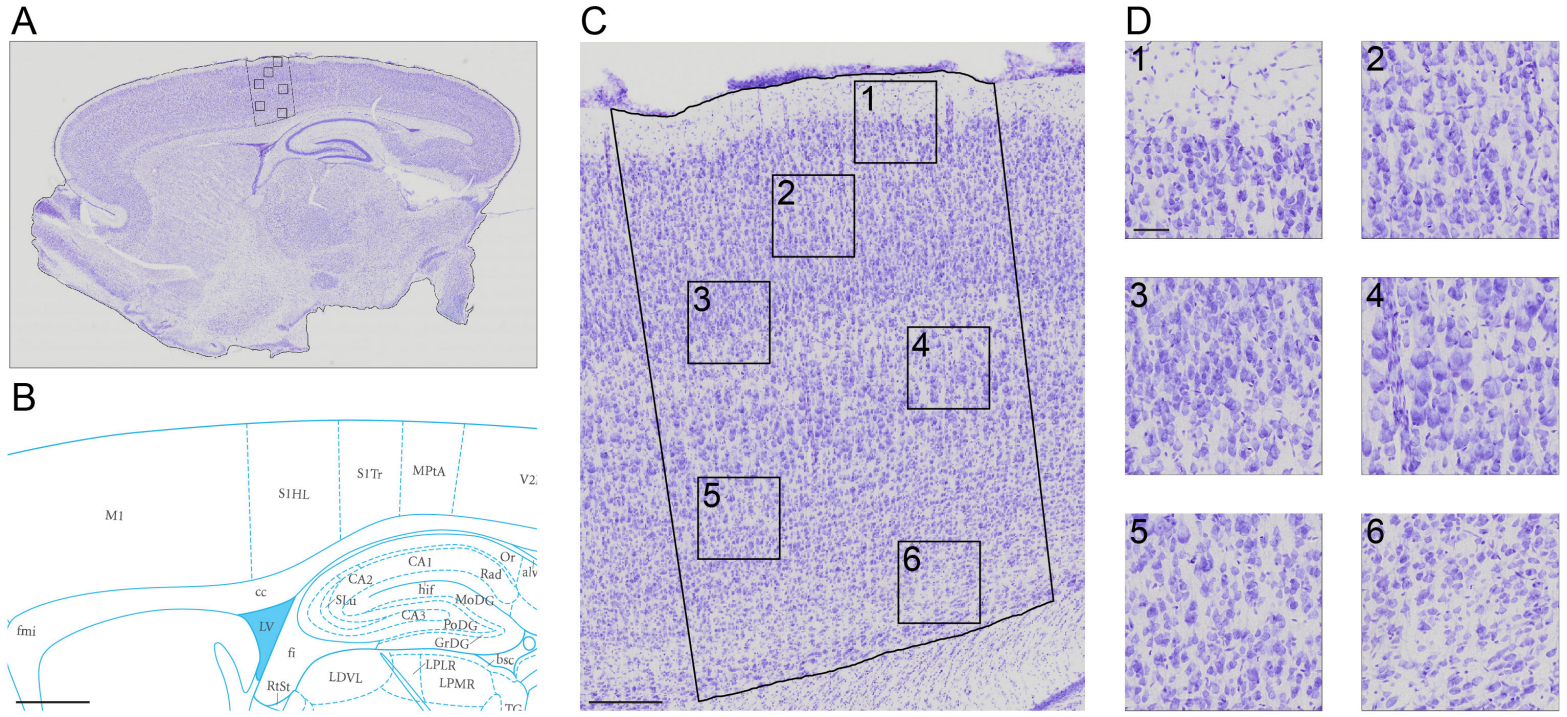
A Nissl-stained sagittal section **(A)** showing the slice contour and the S1HL boundaries (black full lines) **(B)** The corresponding Paxinos and Watson atlas plate (Figure 172—Lateral 2.62 mm) **(C)** A zoom-in of the S1HL brain region **(D)** with close-up images of cells along the cortical column (1) Layer I and Layer II border (2) Layer III (3) Layer IV and Layer V border (4) Layer V (5) Layer V border and Layer VIa and (6) Layer VIb. Imaging pixel size: 0,346 μ*m*/pixel. Scale bars: 1,000 μ*m* **(A, B)**; 250 μ*m* **(C)**; 50 μ*m* **(D)**.

The slice images (Figure 1A) were mapped into particular regions using the corresponding atlas (Figure 1B). The high resolution of 0.346 × 0.346 μ*m* (Figures 1C, D) allows for semi-automatic and automatic cell segmentation for better cell quantification.

Each hemisphere dataset was then prepared in QuPath. Using the Paxinos and Watson atlas as a reference, we began by identifying the most similar slice containing the S1HL and matching the overall slice anatomy. The atlas-described distance to the midline was then assigned to this selected slice. From this reference point the appropriate increments or decrements of 50 μ*m* were applied for all slices of the hemisphere. Among the remaining samples, comprising the S1HL brain region, further selection was performed using specific criteria: slices were disregarded if they were damaged, exhibited a curled configuration, lacked proper visualization of the somatosensory cortex, displayed hydrocephalic brain characteristics, or presented severe CPu atrophy. At the end of this process, we drew the Slice Contour, the S1HL ROI, added S1HL reference points and the Outside Pia contour. *N*_*Slices*_ = 199 remained available for further cell densities analysis.

### 3.2 Cell segmentation

To automatically collect cell positional data from each slice image, we tested a cytoplasm model (“Cellpose—cytoplasm 2.0 model—*cyto*2”). This model was utilized on high-resolution images to effectively detect and segment nuclei within the S1HL region. However, it became evident that this model had limitations in accurately constraining the segmentation or distinguishing the smallest cells. Additionally, it tended to inaccurately identify two adjacent cells as a single larger cell, thereby impacting the accuracy of the segmentation process. Because the data used in this study differed significantly from the training set used for the Cellpose *cyto*2 model (most Cellpose training data consists of homogeneous objects, despite the capability of Cellpose to work with non-homogeneous populations), we refined the *cyto*2 cell segmentation process.

We enhanced the accuracy of cell soma contours, disentangled double-cell instances, and removed artifacts on four images (three squares for validation and twenty-four squares for training, each square size is equal to 177,16 × 177,16 μ*m*). This task was challenging for several reasons: the large number of cells and the need to check and update the contours at each training attempt of the cell detection algorithm. Throughout the process, we adopted a standard software zooming factor of 5.4% to mitigate inconsistencies in drawing precision. This labor-intensive endeavor resulted in the creation of what we refer to as a ground truth cell segmentation dataset (“cell segmentation GT dataset”).

**Figure 2:**
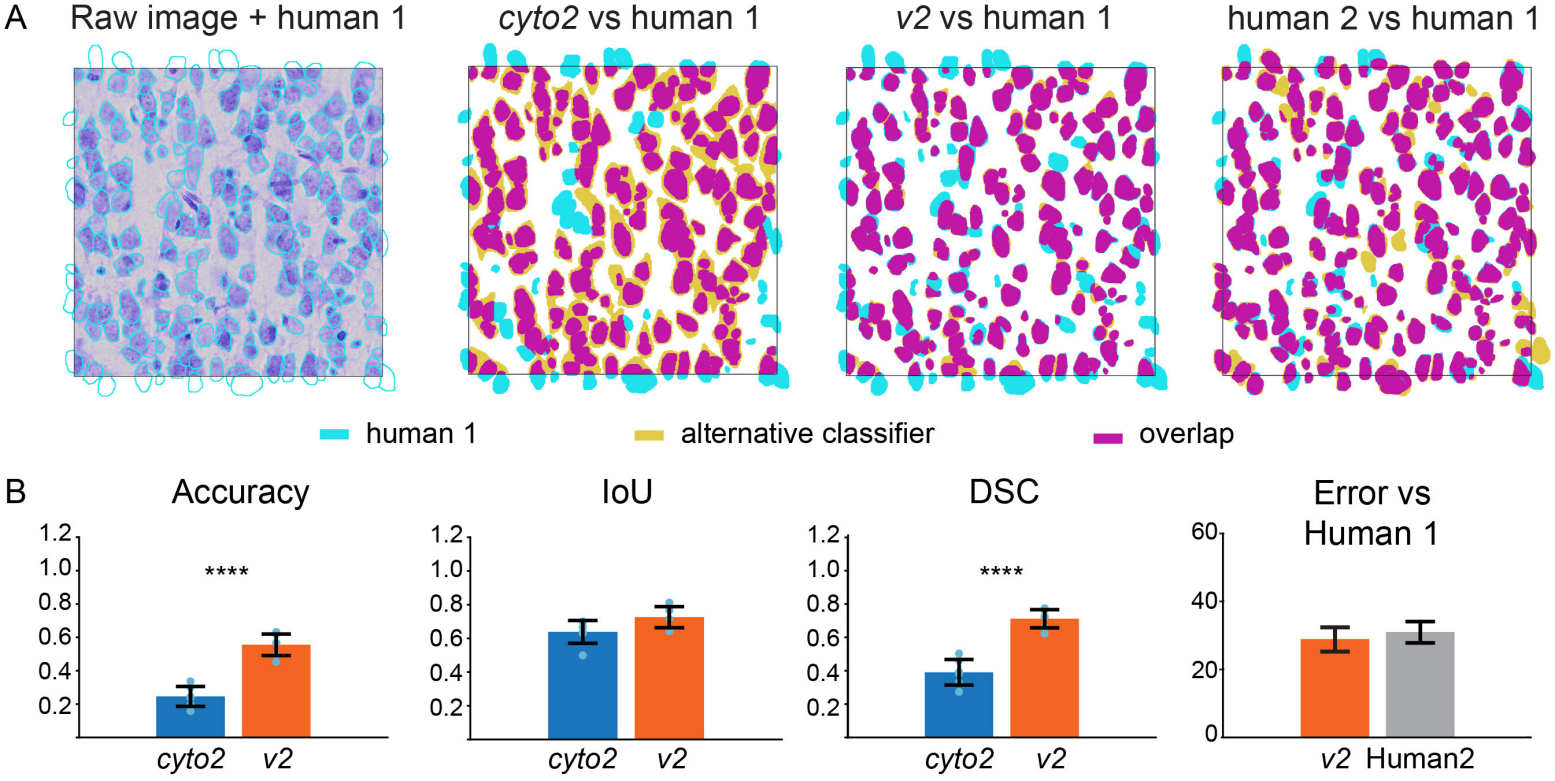
Comparison of the cell segmentation in Layer III **(A)** from left to right: the human annotation is superposed on the raw image, on *cyto*2 and on the final *v*2. The human annotation is represented in cyan, the alternative classifiers in olive and the overlap in purple. **(B)** Quality control measures of the cell segmentation model comparing *cyto*2 (blue) with *v*2 (orange), human 2 (gray), standard deviation (black), Statistical analysis by Welch **T**-test ****p < 0.0001.

We then compared this augmented segmentation version, referred to as *v*2, with *cyto*2 and compared two human annotators (Figure 2A). The accuracy between Human 1 and Human 2 demonstrated a high degree of overlap of 72.5 ± 3.5%. The accuracy between *v*2 and Human 1 was even higher than that of Human 2 74.2 ± 2.5%. These results give us confidence in the reliability of the submitted segmenter as shown in Figure 2B and in Supplementary Figure 1.

### 3.3 Stereology and cell density

**Figure 3:**
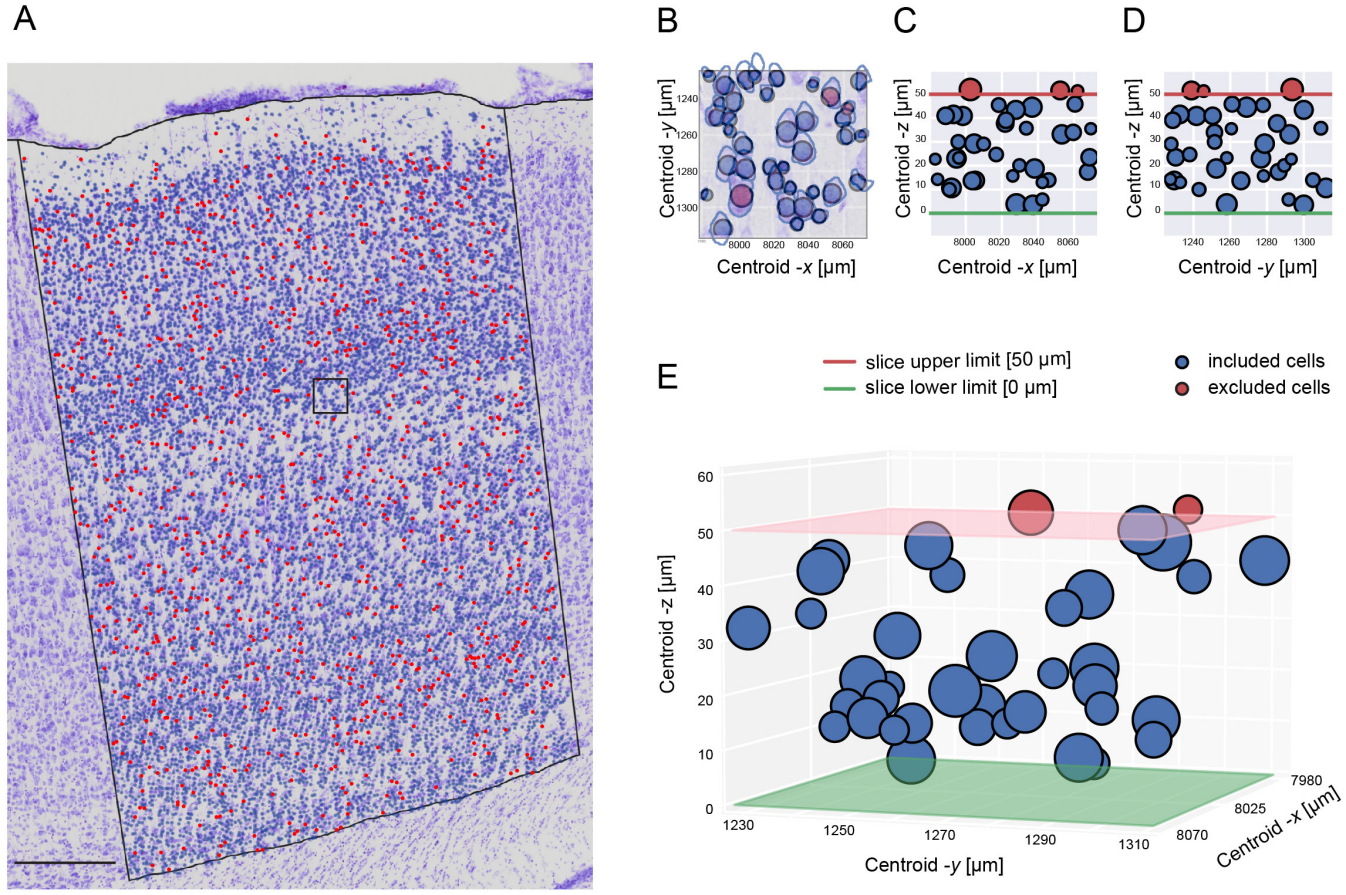
Stereological cell exclusion. **(A)** An example showing the exclusion distribution on one S1HL brain image. Scale bar: 250 μ*m*. **(B)** Zoom-in of the superposition of the raw image with the 2D visualization of the excluded cells in the -*x* and -*y* axis. **(C)** 2D visualization of the excluded cells in the -*x* and -*z* axis. **(D)** 2D visualization of the excluded cells in the -*y* and -*z* axis. **(E)** 3D visualization of the excluded cells in the three axis. **(C–E)** panels shows the exclusion of cells being restricted on the slice upper limit.

As our datasets contained consecutive slices, we adapted the classical optical dissector method by adding a virtual -*z* coordinate to cells to realistically estimate the cell density within our defined volume of interest (see Supplementary Figure 2). The bootstrapping technique excluded only part of the cells (Figure 3A) touching the upper surface of the slice (Figures 3B–E).

**Figure 4:**
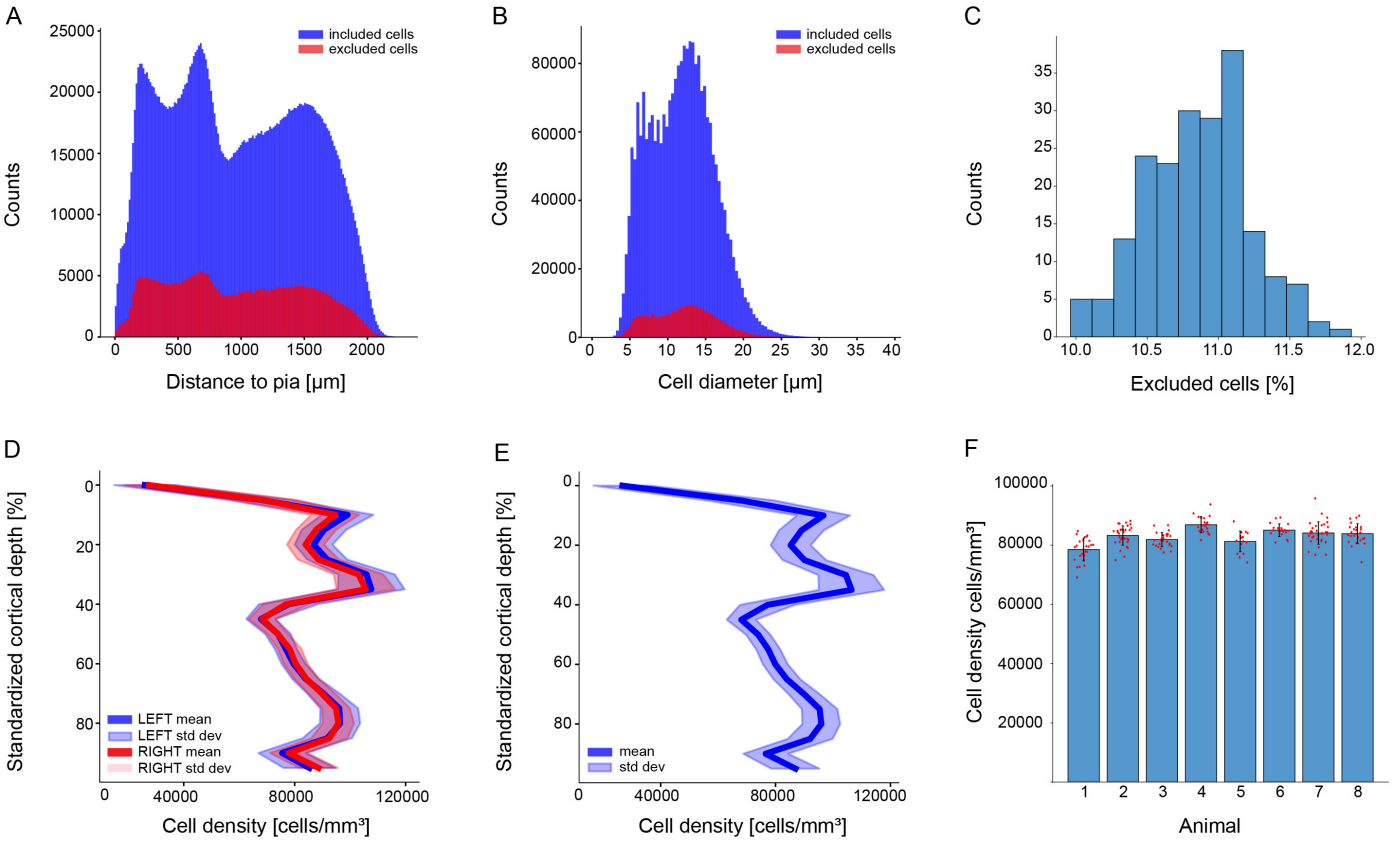
Stereology exclusion result and cell density of the juvenile rat Wistar Han (P14) as a function of percentage of cortical depth of the S1HL brain region. The upper panel shows the stereology exclusion results of the whole dataset. **(A)** Superposition of excluded and included cells per distance to the pia (red for excluded cells and blue for included cells). **(B)** Superposition of the diameters of excluded and included cells (red for excluded cells and blue for included cells). **(C)** The percentage of excluded cells per image. The bottom panel shows cell density as a function of cortical depth with **(D)** the inter-hemisphere variability (right hemispheres mean in dark red, left hemispheres mean in dark blue, standard deviation in light red and light blue), **(E)** the cell density of the whole dataset (mean in dark blue, standard deviation in light blue), and **(F)** the variability between animals (one bar per animal, mean in black, each individual brain slice as a red dot).

A total of 9.79% of the cells were excluded from the analysis *N*_*Image*_ = 199, *N*_*meanDetectedCell*_ = 13690, *N*_*meanExcludedCell*_ = 1340, *N*_*Animal*_ = 8, *N*_*Hemisphere*_ = 14 (7 RH, 7 LH). The proportion of included and excluded cells remains constant throughout the cortical column and the cell soma diameters (Figures 4A–C). This exclusion enhanced the reliability and confidence of our cell density results.

Our data reports no significant differences of the juvenile male rat somatosensory cortex cell densities between the right (*N*_*RightHemisphere*_ = 7) and the left hemisphere (*N*_*LeftHemisphere*_ = 7) (Figure 4D). The sexual dimorphism could not be analyzed as only male datasets were processed.

The cell density curve along the somatosensory cortex is consistent across animals, litters, and experimental days (Figures 4E, F and Supplementary Figure 3). The cell density results for individual brains are shown in Supplementary Figures 4, 5. The total mean density of cells positive for the Nissl substance in the S1HL brain region is 83,100 ± 2,393 per cubic mm (cells/mm^3^). This result is lower than expected (Morin and Beaulieu, 1994; Markram et al., 2015); however, comparing methods, animal age, brain regions, staining molecules or sample size is challenging. Nevertheless, a study from the late 90s reports a barrel cortex cell density of 79,130 ± 26,440 per cubic mm (same strain, older animal, same fixation procedure, similar methodologies, same cresyl violet staining) (Keller and Carlson, 1999).

### 3.4 Cortical layer boundaries

**Figure 5:**
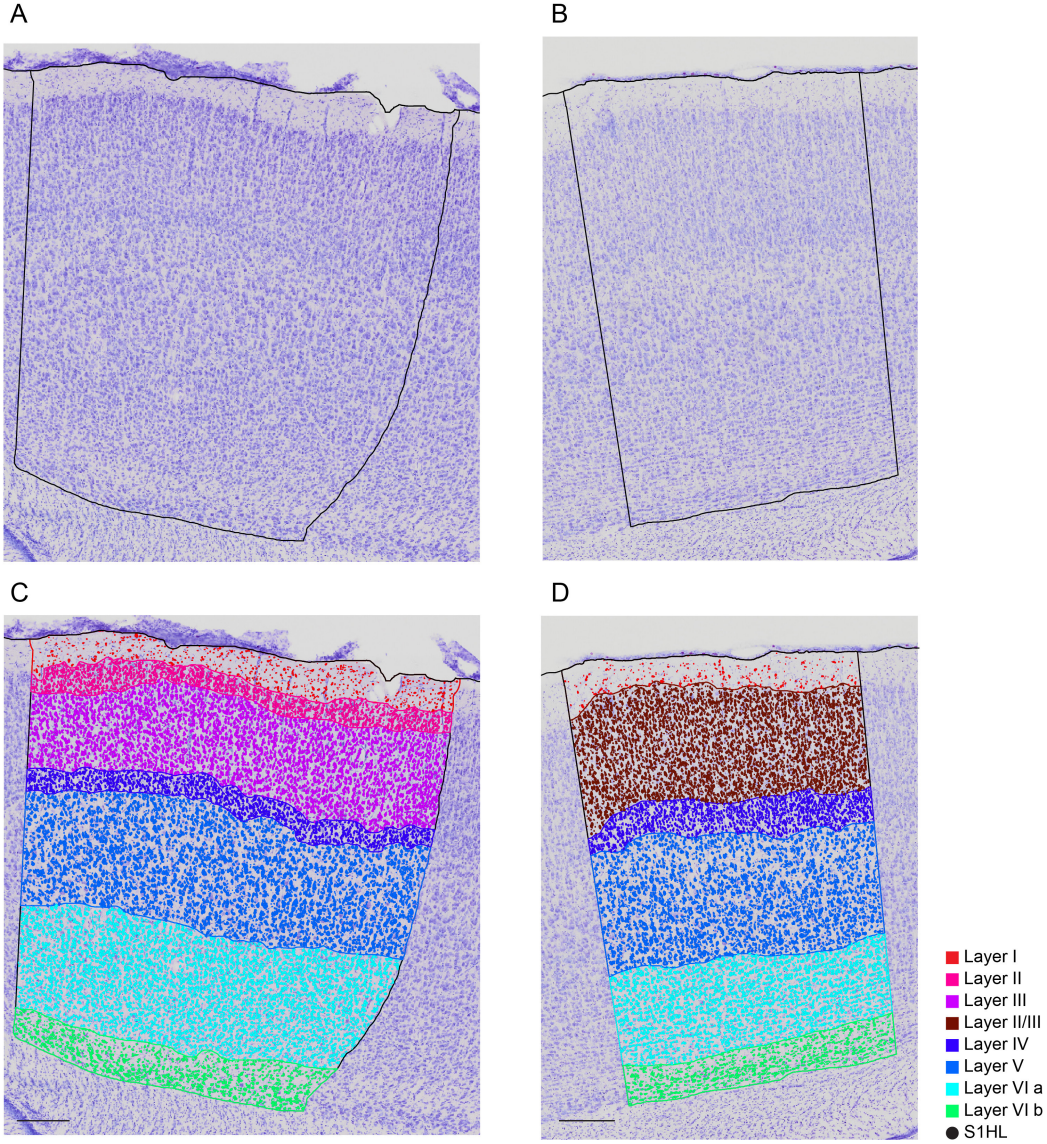
Example of the cortical layering annotated by a human expert. **(A, B)** are the unannotated images of **(C, D)**, respectively. In **(C)** Layer II is distinguished from Layer III (Lateral 2.90 mm). Whereas, in **(D)** Layer II and Layer III are merged (Lateral 2.40 mm). Scale bar: 250 μ*m*. Color code: red (Layer I), pink (Layer II), purple (Layer III), brown (Layer II/III), blue (Layer IV), lighter blue (Layer V), cyan (Layer VIa), green (Layer VIb), black (S1HL).

The boundary between Layer II and Layer III could be distinguished in 30 out of the 38 images (79% of the cases), while we were not able to separate them in the remaining eight images (21% of the cases) (Figure 5A). We therefore decided to label cells belonging to these indistinguishable layers as being part of a merged Layer II/III (Figure 5B).

Anatomically, it is more interesting to train a model on separated Layer II and Layer III data. Therefore, we present here results based on two ML models, RF and KNN, trained on separated Layer II and Layer III. The results on the merged LII/LIII are provided in Supplementary Figures 6, 7. We evaluated other models, such as Gradient Boosting and Feed Forward Neural Networks, but they either showed worse performance or had higher computational cost for similar performance.

Out of the 72 available cell features, 19 were selected to enhance computational efficiency and predictive accuracy. A detailed list of these features with their importance is provided in Supplementary Tables 2, 3. The most critical features include the distance to the annotation with the Outside pia (μ*m*) and its smoothed value (Smoothed 50 μ*m*: Distance to annotation with Outside Pia μ*m*), highlighting the intrinsic spatial dependency of the layers. Those two features individually resulted in a decrease in accuracy of 0.2288 ± 0.018 % and 0.1902 ± 0.0015 % when permuted.

To avoid drastic dataset shrinking, we included cells from every image and excluded only those from the undistinguishable Layer II and Layer III. This dataset was then split into 32 images for training, representing 395,926 cells, while the remaining six images containing 76,515 cells were used for testing.

**Figure 6:**
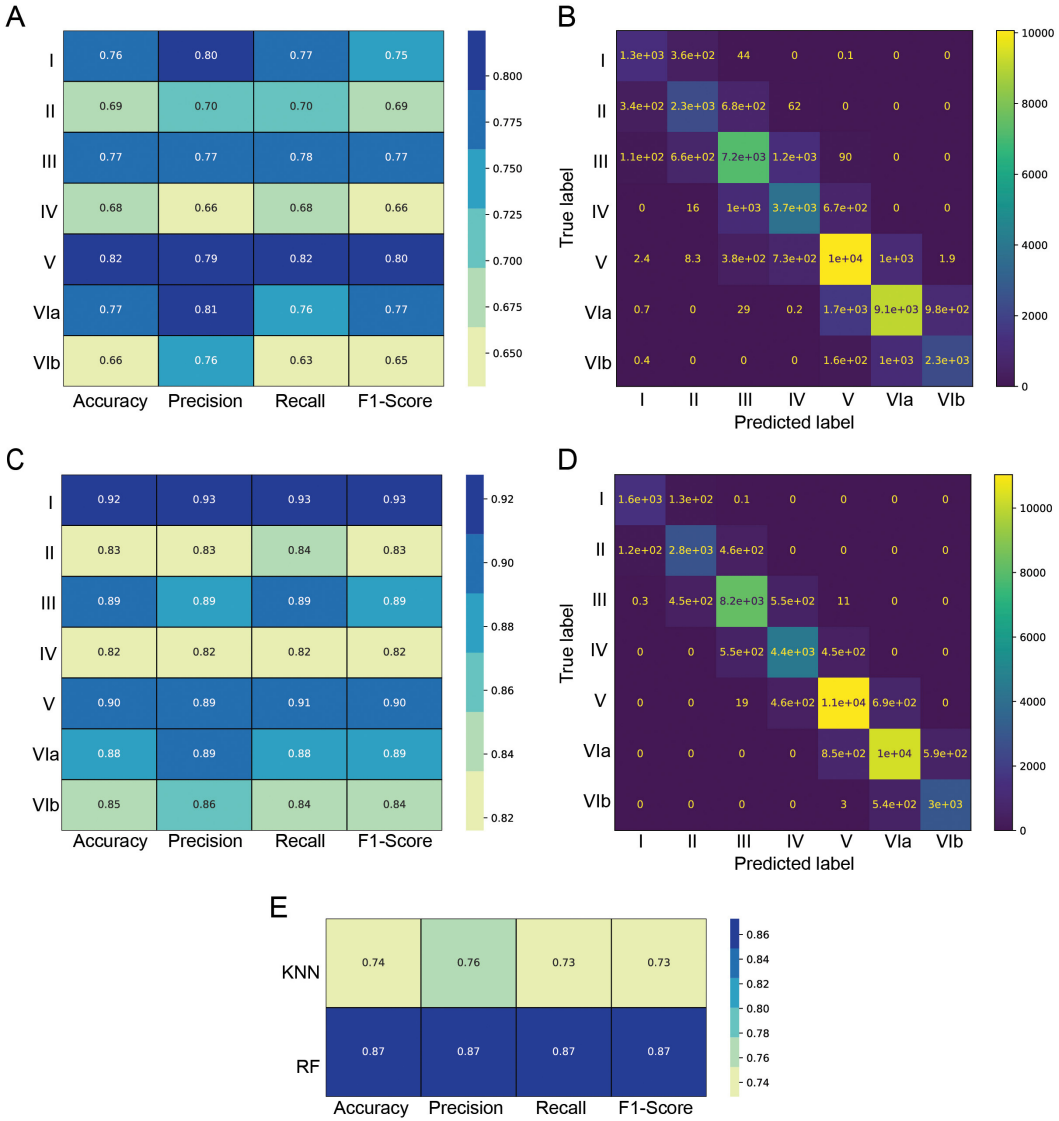
Classification metrics for KNN and RF models on Layer II and III separated. **(A)** KNN generated classification metrics per layer, **(B)** confusion matrix of the KNN model **(C)**, RF generated classification metrics per layer **(D)**, confusion matrix of the RF model, **(E)** overall per-model metrics.

We evaluated KNN and RF performance using four metrics: accuracy, precision, recall and F1-score. The evaluation shows that Layer II, IV and VIb are the most challenging to classify for both models (panel A for KNN and panel C for RF of Figure 6).

A confusion matrix, where each entry represents the number of cells classified into each combination of ground truth and predicted labels is shown in panel B for KNN and panel D for RF of Figure 6. The result shows that almost every cell is either classified in the correct layer, or in the adjacent one. This underlines that the models captured the sequential nature of the layers and that no neighbor cells in space should pertain to distant classes. The results clearly demonstrate the superior performance of the RF model over the KNN, across all metrics and all layers.

We averaged the per-class metrics for each model, ensuring that classes with varying sample sizes contribute equally to the final metric values. The overall KNN prediction accuracy reaches 74%, whereas the RF prediction accuracy is higher and seems to be capped at 87% for separated Layer II and Layer III (Figure 6E). This could be partially influenced by the approximate nature of the ground truth annotation by a human, especially around the layer boundaries. Supplementary Figure 8 compares the results of (Figure 6E) with classes with more samples having a proportionally higher weight in the metrics and shows a minimal difference due to an imbalance of samples.

**Figure 7:**
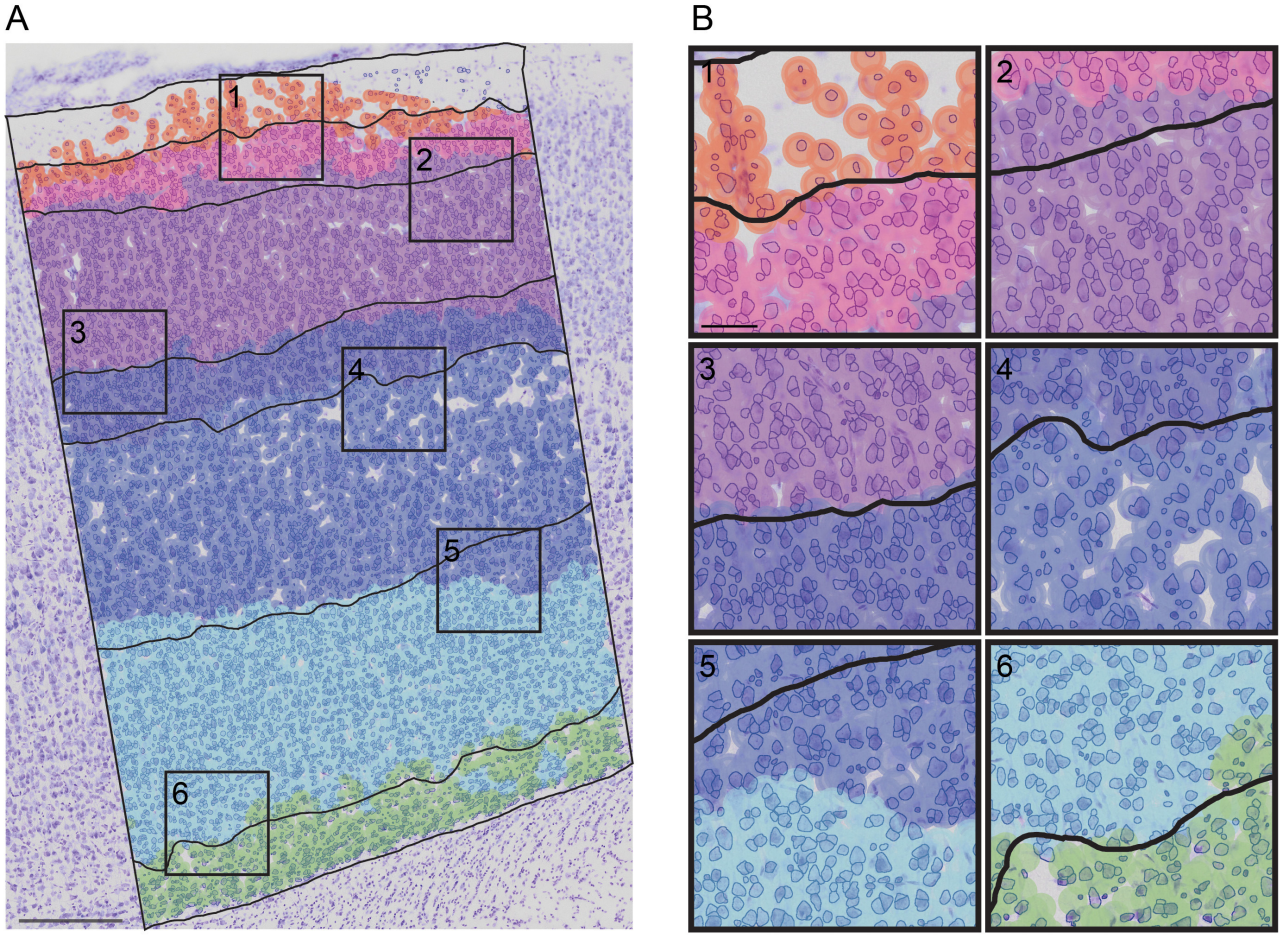
A superposition of the RF prediction onto the RAW image with the cell segmented and the human-annotation superposed. **(A)** RF prediction and human-annotation (black lines) superposed onto the RAW image with the cell segmented (blue). **(B)** close-up of each layer borders predictions [(1): LI to LII border; (2) LII to LIII border; (3) LIII to LIV border; (4) LIV to LV border; (2) LV to LVIa border; (2) LVIa to LVIb border]. Scale bar in **(A)** 250 μ*m* and in **(B)** 50 μ*m*.

To further assess the predictions of cortical layers, we visually evaluated them on test images for their biological relevance (Figure 7A; Supplementary Figure 9). A close-up of the RF predictions superposed onto the human annotation is shown in Figure 7B.

As RF outperformed KNN, we applied it to the remaining unannotated *N*_*Images*_ = 262 to predict the S1HL layer boundaries of each image and we visually verified the predicted layers. This drastically increased the layer annotation throughput a human-annotator would reach.

### 3.5 Cell densities and size prediction

**Figure 8:**
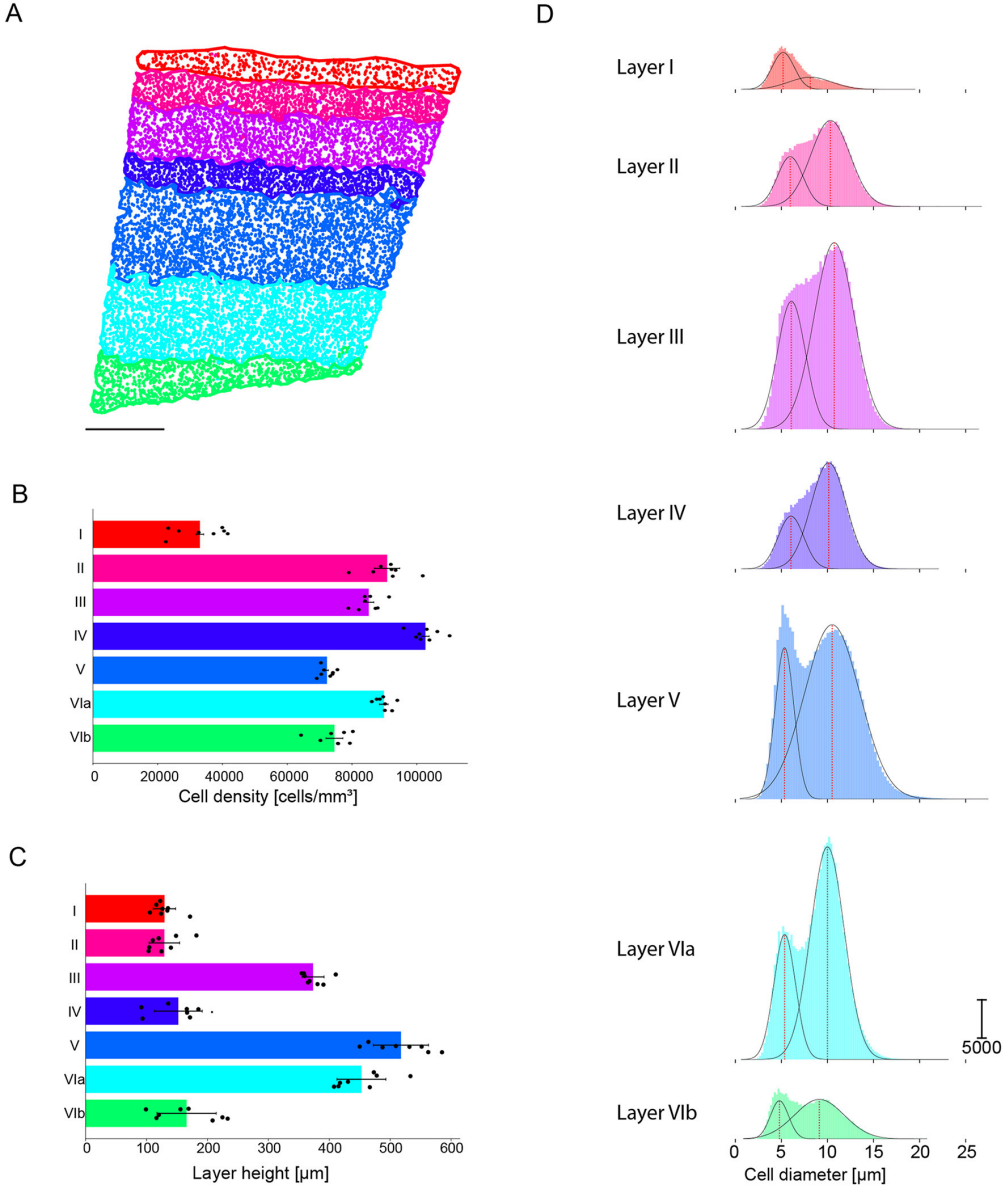
Example of cortical layer boundaries generated with the alpha shape toolbox. **(A)** Region of interest with cell bodies colored according to layer. Scale bar: 250 μ*m* **(B)** Cell density per predicted layer [cells/mm^3^], averaged across all samples, with each orange dot representing one animal. **(C)** Predicted layer height [μ*m*], averaged across all samples, with each orange dot representing one animal. **(D)** Mean S1HL cell diameter [μ*m*] per layer under the assumption of a perfect circular morphology. Black dotted lines the Gaussian and red dotted line the center peak of the fit Gaussian. **(E)** Predicted S1HL cell density per animal (each bar plot represents an animal) [cells/mm^3^].

Because the cell density only varies slightly between images, we aimed to find a single α value, for which the bounding area enveloping each layer was as close as possible to the layer shape and that would not reject any cells (Figure 8A). The value of 0.05 is applied on all layers, with the exception of Layer I characterized by a significantly lower cell density, for which we opted for a value of 0.005.

We then counted the number of cells located in each alpha shape polygon corresponding to each layer and computed the volume of each polygon by using the theoretical slice thickness of 50 μ*m*. Consequently we found the following mean densities: Layer I contains 32,568 cells/mm^3^, Layer II contains 90,226 cells/mm^3^, Layer III contains 85,094 cells/mm^3^, Layer IV contains 103,016 cells/mm^3^, Layer V contains 72,005 cells/mm^3^, Layer VIa contains 89,198 cells/mm^3^, Layer VIb contains 71,153 cells/mm^3^ (Figure 8B; Table 2). The cell density per predicted layer and per hemisphere is shown in Supplementary Table 4.

**Table 2:** The cell density per predicted layer (cells/mm^3^).

	**Cell density per predicted layer (cells/mm^3^)**	**Predicted layer height (*μm*)**	**Cell diameter per predicted layer (** ***μm*** **)**		
			**Mean (**μ*m***)**	**Population 1 (**μ*m***)**	**Population 2 (**μ*m***)**
Layer I-VIb Overall cortical height		1,915 ± 50.57			
Layer I	32,568	129 ± 18	6.47 ± 2.27	5.20 ± 1.20	8.14 ± 2.33
Layer II	90,226	129 ± 25	9.17 ± 2.73	5.96 ± 1.38	10.33 ± 2.11
Layer III	85,094	373 ± 18	9.26 ± 2.86	6.09 ± 1.52	10.74 ± 2.18
Layer IV	103,016	152 ± 39	9.09 ± 2.53	6.04 ± 1.40	10.14 ± 1.91
Layer V	72,005	517 ± 45	9.38 ± 3.42	5.35 ± 1.03	10.51 ± 3.00
Layer VIa	89,198	452 ± 40	8.80 ± 2.58	5.36 ± 1.18	10.01 ± 1.83
Layer VIb	71,153	165 ± 48	8.03 ± 2.94	4.80 ± 0.99	9.12 ± 2.69

The predicted layer height (μ*m* ± standard deviation between animals in μ*m*). The mean soma diameter per predicted layer (μ*m*), with the larger population represented in Population 1 and the smaller in Population 2. *N*_*Hemispheres*_ = 13 (*N*_*Right*_ = 7, *N*_*Left*_ = 6).

Using the layer prediction generated for each cell by the machine learning model, we determined the height of each layer by fitting the cell densities predictions. The height of each layer is the distance between the top and the bottom of a layer. The projected height of the S1HL cortical column is 1,915 μ*m* with a standard deviation of 50.57 μ*m*, detailed height per layer is reported in Figure 8C and in Table 2. These projections are consistent with previously published data (Beaulieu, 1993; DeFelipe et al., 2002; Markram et al., 2015; Meyer et al., 2010).

Once a predicted layer was assigned to each cell, the distribution of cell diameters was calculated under the assumption of a perfect circular morphology of the same area (Figure 8D). The somatosensory cortical column contains two distinct cell populations, with a cell diameter distribution ranging from 0.39 to 27.57 μ*m*; each of the populations consists of multiple subtypes. The first population of small cells spans from 4.80 to 6.09 μ*m* and is present throughout the entire cortical column height. The second population of larger cells peaks from 8.14 to 10.74 μ*m* and is present in all layers except Layer I. Layers I, V, and VIb contained a larger proportion of smaller cells, whereas Layers II, III, IV, VIa contained a larger proportion of larger cells. Supplementary Figure 10 shows the cell diameter distribution per layer, which is well-described by two Gaussian distributions. Supplementary Table 5 displays the mean values for the two Gaussian distributions of cell diameters calculated per image and per layer. To determine the statistical significance of cell density differences across the layers, a Kruskal-Wallis *H*-test was performed on data obtained from 228 images. This confirmed that cell densities are different across layers and that they are not drawn from the same distribution. The last column of Supplementary Table 5 also shows that cell densities are significantly different across each pair of adjacent layers.

We also confirmed that other cortical regions could be processed similarly with our approach by applying it to the medial parietal association cortex - MPtA (see Supplementary Figure 11).

## 4 Discussion

We have produced the most detailed publicly available dataset of Nissl-positive cells in a rodent species to date, accompanied by an open-source, semi-automatic cell detection pipeline. This pipeline accurately segments and counts the total number of cells within the somatosensory hindlimb cortical column of eight juvenile rats, stained using a traditional Nissl method. By incorporating machine learning models, the pipeline efficiently assigns layer boundaries and categorizes segmented cells accordingly. The Cellpose algorithm was particularly well-suited to the challenge of dense cell segmentation. We expect that these pipelines could be adapted for use with other staining methods, as long as the sample preparation and acquisition parameters support precise cell annotation for generating reliable ground truth data.

As demonstrated in the original Cellpose paper, the IoU comparison of different models (Cellpose, Mask R-CNN, Stardist, U-Net3 and U-Net2) shows Cellpose's superior precision (doi: 10.1038/s41592-020-01018-x; Figure 4). While Cellpose may struggle with cell overlap, this issue is mitigated using thin (50 μ*m*) slices and has little effect on the classification of brain regions based on cell shape and localization features.

Although superposition of cells may influence the overall cell density results, it does not compromise the layer border properties. We were aware of this limitation during the training phase. Cytoarchitectonic distortions, such as concave/convex cell shapes, do not pose a significant challenge to our analysis. Even when mask convexity decreases, leading to a potential increase in missed cells, Cellpose still performs better than other contemporary models (Stringer et al., 2021).

We adapted the stereological exclusion method to estimate the total number of cells in the S1HL without skipping slices. The exclusion factor (roughly 10%) may be on the higher end, and alternative exclusion analysis methods should be explored in the future. Nevertheless, our results show that the overall cell density is lower than initially expected but remains remarkably consistent across individual animals, litters, and experimental days. A study using similar methods also reported comparable cell density in the barrel cortex.

Ever since Broca reported the left hemisphere's dominance for language (Broca, 1861), brain lateralization has been studied both behaviorally (Güntürkün et al., 2020; Rogers et al., 2013) and anatomically (Elkind et al., 2023; Tobet et al., 1993). Previous data from mice report some variation within the cortical layers (LII/III tendency for left hemisphere being denser than the right hemisphere; LIV and LVI with a tendency for right to be denser than the left; see Figure 3C of Elkind et al. (2023). Our data reports no significant differences in the juvenile male rat somatosensory cortex cell densities between the right and the left hemisphere.

However, comparing datasets in this field is inherently complex due to species and age differences. Additionally, differences in brain regions, fixation protocols, staining techniques, sample sizes, and potential human biases complicate direct comparisons between studies.

Our dataset offers an opportunity for further analysis, beyond the metrics explored in this study, and can be adapted for use in other species. We have high confidence in the dataset's quality, due to its exceptional imaging resolution, the standardized methods employed during data collection, and the remarkable reproducibility of the results. While this investigation focused on the somatosensory cortex, the versatility of the dataset allows for potential applications to other brain regions.

We employed machine learning algorithms to categorize cells into layers, minimizing human bias and achieving an accuracy of 87%. Unlike studies that rely on tissue-level differentiation (Wagstyl et al., 2020), our method provides a cell-level classification, offering finer granularity. This accuracy is on par with recent studies (Štajduhar et al., 2023), despite differences in species, staining techniques and acquisition parameters. Direct experimental validation of layer boundaries using a combination of cell-specific stains remains challenging. Staining data, such as receptor profile curves in the isocortex, are often blurred and the localization of stained layers rarely aligns precisely to cytoarchitectonic boundaries (Palomero-Gallagher and Zilles, 2019).

In our classification approach, spatial location was the most critical factor for layer assignment, which is unsurprising given the columnar organization of the cortex. Since all cells must be assigned to a layer, spatial information played a dominant role, particularly for cells within a given layer. For cells at the layer boundaries, morphological features were more influential. Future studies could explore models trained specifically on these boundary cells, where morphology would likely have a greater impact on classification decisions. While our study aimed to reduce human bias in layer assignment, it is important to note that different brain regions would require re-training of machine learning models to account for their unique properties. Classifying cells situated at layer boundaries remains a significant challenge compared to those located inside layers. Once a layer is defined by the model, cells within that layer can be classified based on their distance from the pia. Future studies should prioritize the development of metrics to evaluate boundary prediction accuracy and explore methods that do not rely solely on spatial information for boundary cells, such as training distinct models for this specific population.

The accuracy of automated annotation may fluctuate in regions characterized by unique neuronal properties or when utilizing different staining techniques. As our approach relies on supervised machine learning, the precision of cell prediction is contingent upon the expertise of human annotators. Nonetheless, our method lays the groundwork for more efficient and standardized cortical layer annotation. Notably, the predicted layer thicknesses in our study are consistent with those reported in previous studies on the barrel cortex (Meyer et al., 2013; Narayanan et al., 2017).

Our study also provides a more detailed characterization of the somatosensory cortex by analyzing the distribution of cell soma diameters within each predicted layer. We identified two primary cell populations: a smaller population with a cell diameter ranging from 4.8 to 6.1 μ*m* and a larger population with a cell diameter ranging from 8.1 to 10.7 μ*m*. The exact locations of the peaks differed slightly according to layer. The smaller population, present throughout the cortical column, is likely composed of inhibitory cells, especially since Layer I predominantly contains these small cell types (Muralidhar et al., 2014). The population with larger diameters likely includes excitatory cells, as it is absent in Layer I but present in all other layers. One should remember that our dataset includes not only neuronal cells but a diverse range of cell types including astrocytes, oligodendrocytes, neurons and glial cells. Accordingly, the cell size difference alone may not be sufficient to definitely categorize them as inhibitory or excitatory neurons.

By automating cell classification into cortical layers, our methodology accelerates a traditionally labor-intensive task. Accurate quantification of cell densities in brain tissues, enabled by our approach, holds significant potential for advancing our understanding of neuropathologies, such as neurodegenerative diseases that affect specific cell populations. Additionally, this work supports the construction of detailed cortical microcircuit models, contributing to a more comprehensive understanding of brain structure. Ultimately, our study lays the groundwork for fully automated, high-throughput investigations leveraging the wealth of histological data being produced worldwide, enabling more efficient exploration of the complexities of cortical microstructure.

## Data Availability

The datasets presented in this study can be found in online repositories. The names of the repository/repositories and accession number(s) can be found below: Nissl_1: https://doi.org/10.5281/zenodo.12204988; Nissl_2: https://doi.org/10.5281/zenodo.12514990; Nissl_3: https://doi.org/10.5281/zenodo.12516971; Nissl_4: https://doi.org/10.5281/zenodo.12516982; Nissl_5: https://doi.org/10.5281/zenodo.12517030; Nissl_6: https://doi.org/10.5281/zenodo.12517073; QuPath_LayerBoundaries_GroundTruth_20220927: https://doi.org/10.5281/zenodo.12656468. The source code for the analysis is available at: https://github.com/BlueBrain/layer-recognition.
