# Supplementary material for: Cell density quantification of high resolution Nissl images of the juvenile rat brain
Source: Front Neuroanat. 2024 Dec 18;18:1463632. doi: 10.3389/fnana.2024.1463632 (PMC11688196; doi:10.3389/fnana.2024.1463632)
Supplement: Supplementary file 1 [file Data_Sheet_1.PDF]

Layer Boundaries Project – Cell segmentation

Date: 30 May 2022, JulieMeystre (last edited 04 Jul 2024)

Context

This document describes:

1. Which and how the cell segmentation algorithm is trained in the context of the Layer Boundaries project (with examples of cell results)
2. The semi-automated pipeline to trigger cell segmentation from a QuPath project and provide an analysis of the different cell densities along the cortical column.

Contents

Layer Boundaries Project – Cell segmentation 1
Context 1
Algorithm training – Stardist – model 1 and 2 3
General 3
Results 4
Images detail 5
Algorithm training - Cellpose 7
General 7
Summary 7
Inclusion and exclusion factors 7
Cellpose - Version 0 8
Cellpose - Version 1 - capillaries partially excluded *20240704 jm: Project: QuPath_Training_2_20220923.qpproj* 11
Cellpose - Version 2 – capillaries excluded 17
Cell segmentation - Cellpose 27
Data availability 27
QuPath objects to create (here for the 2021_Layer Boundaries Project) 27
How to Setup and use the BIOP-QuPath-Cellpose extension v0.1 28
Bibliography 31
Addendum 31
Run StarDist.groovy 31
Convert StarDist Detections to Annotations.groovy 32
Run-Cellpose_Default_cyto2.groovy 32
Run-Cellpose_Trained_Julie_1200_Epochs_Full.groovy 33
Run-Cellpose_Trained_Julie_1200_Epochs_Downsample_2.groovy 33
Run-Cellpose_Trained_Julie_1200_Epochs_Full_DBSCAN.groovy 34
Run-Cellpose_20220113.groovy 34
Train-Cellpose-Model_202220110.groovy 35
1. Run CellposeCyto2ModelPrediction.groovy 35
2. Run Cellpose V1 Model Prediction.groovy 36
2.Run Cellpose V2 Model Prediction.groovy 36

Algorithm training – Stardist – model 1 and 2

Two algorithms available in QuPath are tested: Stardist and Cellpose.

General

StarDist is an ImageJ/Fiji plugin, a cell/nuclei segmentation method for microscopy images with star-convex shape priors. The plugin can be used to apply already trained models to new images. See the main repository for links to our publications and the full-featured Python package that can also be used to train new models: https://imagej.net/plugins/stardist

Uwe Schmidt, Martin Weigert, Coleman Broaddus, and Gene Myers. Cell Detection with Star-convex Polygons. International Conference on Medical Image Computing and Computer-Assisted Intervention (MICCAI), Granada, Spain, September 2018.

Inclusion and exclusion factors

RordrigoPerin defined the inclusion factors:

- Astrocytes
- Oligodendrocytes
- if two cells are faint and on top of each other, count them as one cell
- if a dark small cell is present inside a fainter one, count them as two cells.

(20210520 RP)

The following exclusion factors were defined:

- capillaries endothelial cells
- the thick layer of cells above pia/layer I

(20210520 RP)

Training notebook, Model, Scripts, Project Folder

Training notebook: \LBR_Algorithm\StarDist\Training Notebooks\

- 1_Load_And_Test_User_Data-Julie.ipynb
- 2_Train_StarDist_Model-RGB_Julie.ipynb

Models: \LBR_Algorithm\StarDist\Models\

- StarDist Model version 1: 'julie-1-slice_nissl_r16_p128_g2_k3_e400_se100_b32_aug'
- StarDist Model version 2: 'julie-nissl-round2_nissl-2_r16_p128_g2_k3_e400_se100_b32_aug'

QuPath Scripts: \LBR_Algorithm\StarDist\Scripts\

- `'Run Stardist.groovy'`
- `'Convert StarDist Detections to Annotations.groovy'`

Project folder:

- \LBR_Algorithm\StarDist\QuPath_StarDist_Training_v1\QuPath_StarDist_Training_v1.qpproj \

Images used:

- SLD_0000463.vsi - 20x_04 (5 training regions, 1 validation region; region area μm^2 125535)
- SLD_0000464.vsi - 20x_01 (5 training regions, 1 validation region; region area μm^2 125535)

Results

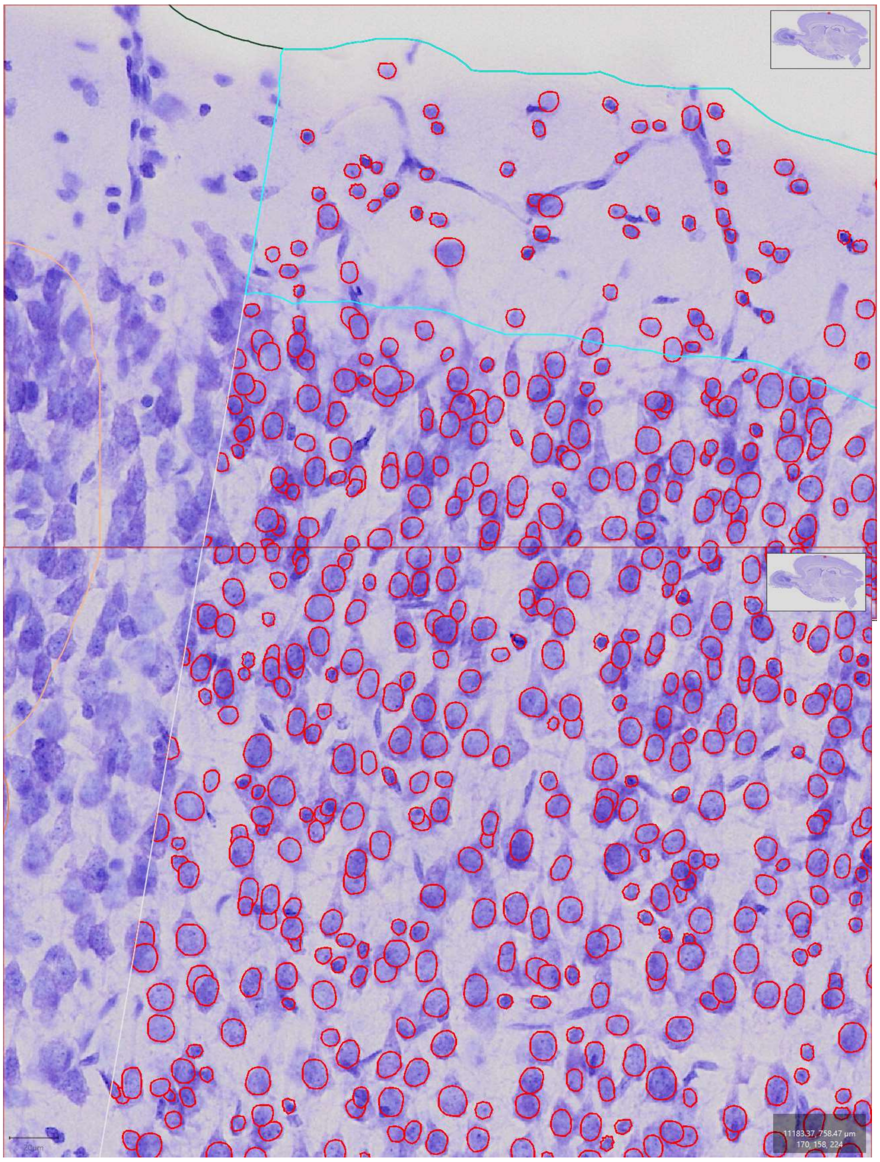

Model version 1: Cells are not well separated and the contour is not following the actual cell contour.

Model version 2 (28 Oct 2021 JulieMeystre): Two iterations were performed to improve cell segmentation without success. When annotating cells, we excluded on purpose tiny cells in one region (WhiteMatter) but not in others (Layer 1 to Layer 6); the algorithm seems lost and less accurate than in version 1. It does not follow well the cells contour either. We decided to abort training StarDist and to test another cell segmentation model, Cellpose, a cell segmentation model with less constraints on the cell shape than StarDist and which training can be done directly in QuPath.

Images detail

SLD_0000463.vsi - 20x_04 (5 training regions, 1 validation region; region area µm^2 125535)

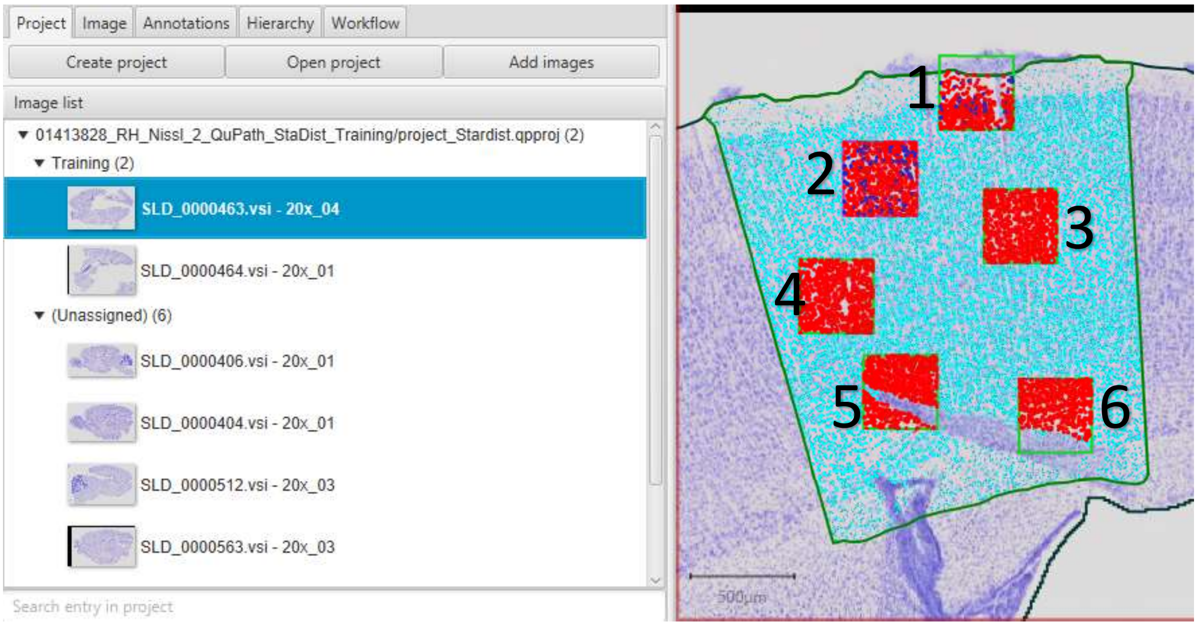

Region 1
Training

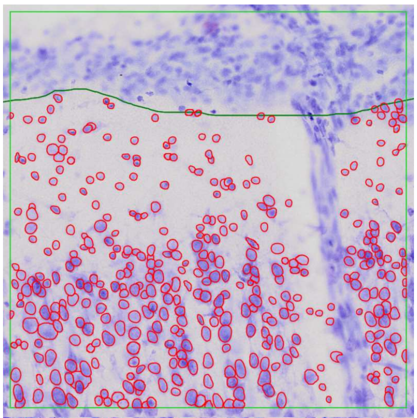

Region 2
Validation

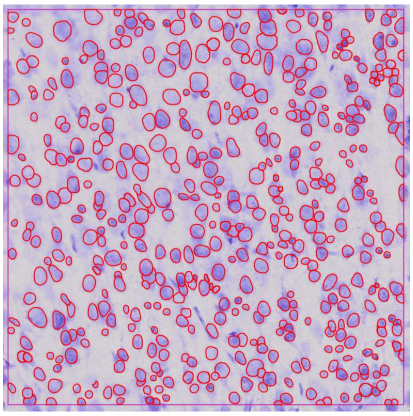

Region 3
Training

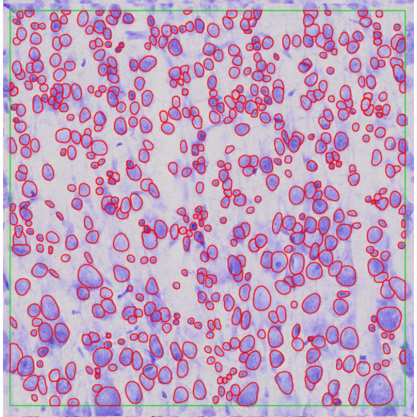

Region 4
Training

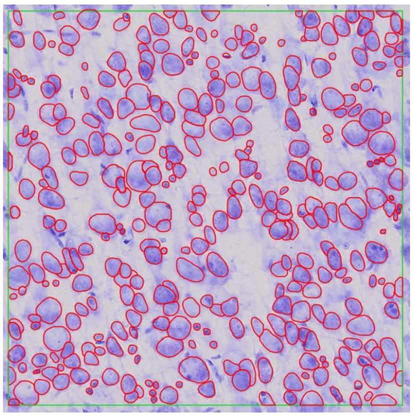

Region 5
Training

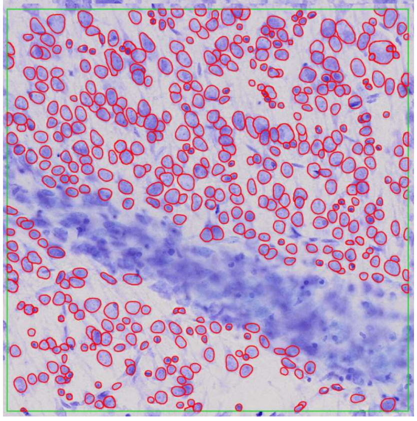

Region 6
Training

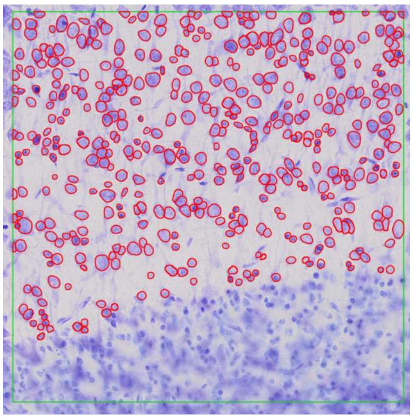

SLD_0000464.vsi - 20x_01 (5 training regions, 1 validation region; region area µm² 125535)

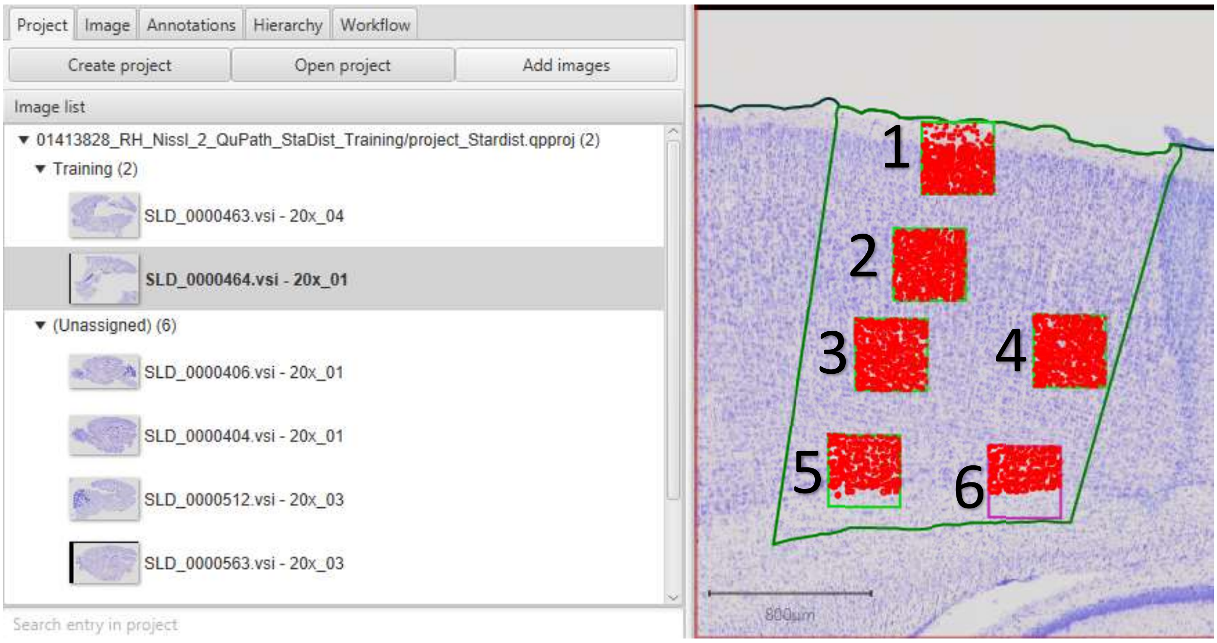

Region 1
Training

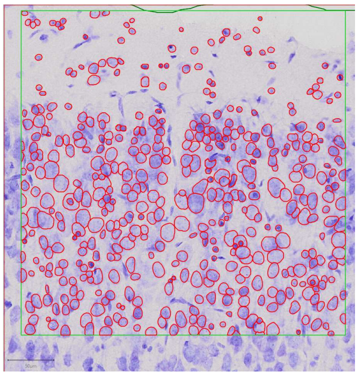

Region 2
Training

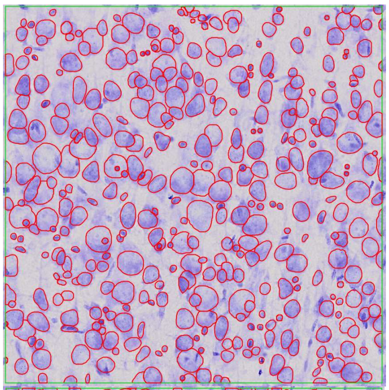

Region 3
Training

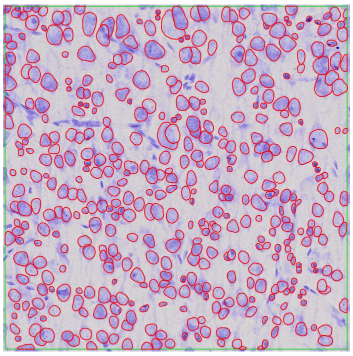

Region 4
Training

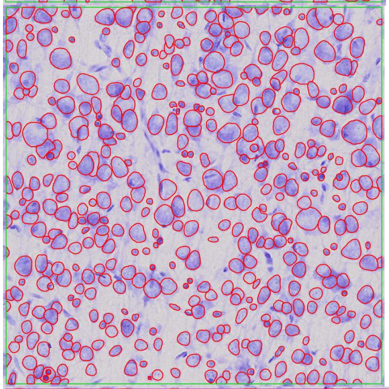

Region 5
Training

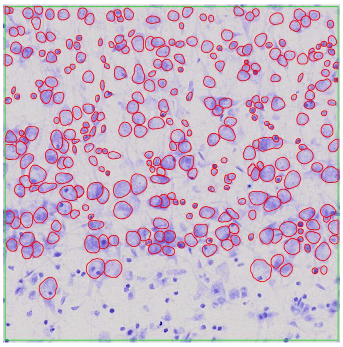

Region 6
Validation

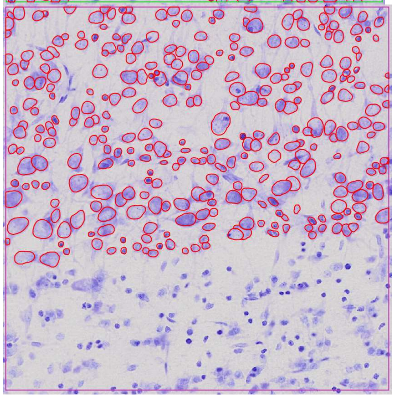

Algorithm training - Cellpose

General

Cellpose is a generalist, deep learning-based segmentation method, that can precisely segment cells from a wide range of image types and does not require model retraining or parameter adjustments. Cellpose can be trained directly inside QuPath.

Stringer, C., Wang, T., Michaelos, M. et al. Cellpose: a generalist algorithm for cellular segmentation. Nat Methods 18, 100–106 (2021). [*https://doi.org/10.1038/s41592-020-01018-x*](https://doi.org/10.1038/s41592-020-01018-x)

Summary

Cellpose was sequentially trained on images with pixel size (x, y): 0,346 x 0,346 µm

1. Cell segmentation from the Cellpose model available ('Cyto2') → result not accurate, missing tiny cells and difficulty to decluster → described as v0 (''Cyto2'')
2. Modify manually the segmentation on several images (multiple users)
3. Train model with the modifications → described as v1 (''Julie Annotation''; ''Julie Annotation downsampled''; ''Julie Annotation DBSCAN'') → *Result*: the annotation modifications 'Julie Annotation' improved the segmentation. Neither downsampling the image nor using the DBSCAN model improved it, rather it made it worst.
4. Modify the v1 segmentation (cells on top of each other counting as one instead of two in v1; rounded cell shape at the border of the regions)
5. Train model with the modifications → described as v2 (Jan 2022)
6. **Version v2 was selected to detect cells on the Rat sample datasets ("Layer Boundaries Refinement" project)**

Inclusion and exclusion factors

RP defined the inclusion factors: astrocytes; oligodendrocytes; if 2 cells are faint and on top of each other count as 1 cell; if dark small cell inside a fainter one count as 2 cells. 20210520. The following exclusion factors were defined: capillaries, endothelial cells, the thick layer of cells above pia/layer I. 20210520 RP.

Cellpose - Version 0

Training notebook, Model, Scripts, Project Folder

Training notebook: None, done directly inside QuPath

Model and model parameter:

- Cellpose Model : `'cyto2'`
- Model parameters: pixelSize(0,3460); TileSize(2048); Diameter(30); PathModel = 'cyto2'

Scripts:

- *`'1. Run CellposeCyto2ModelPrediction.groovy'`* (20211028, edited 2024 EPFL-PTBIOP-OlivierBurri)

Project folder:

- /LayerBoundariesProject/LBR_Algorithm/Cellpose/QuPath_Training_2_20220923/QuPath_Training_2_20220923.qpproj

Images used:

- SLD_0000463.vsi - 20x_04 (5 training regions, 1 validation region; region area µm^2 125535)
- SLD_0000464.vsi - 20x_01 (5 training regions, 1 validation region; region area µm^2 125535)

Results

Default segmentation model «Default cyto2»: It misses cells, especially big ones. Cells are round shape. Has difficulty separating cells cluster.

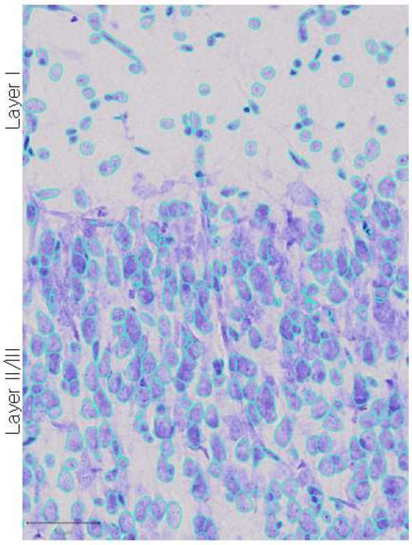

Images detail

SLD_0000463.vsi - 20x_04 (5 training regions, 1 validation region; region area μm^2 125535)

Region 1
Training

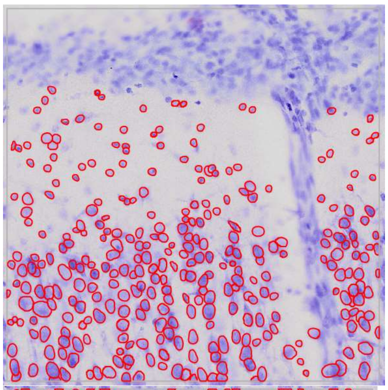

Region 2
Validation

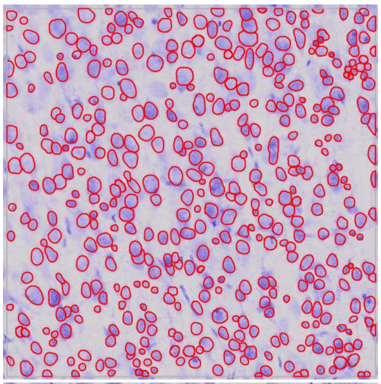

Region 3
Training

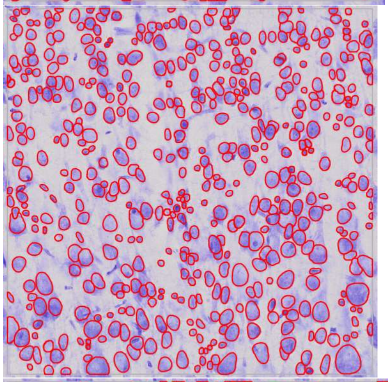

Region 4
Training

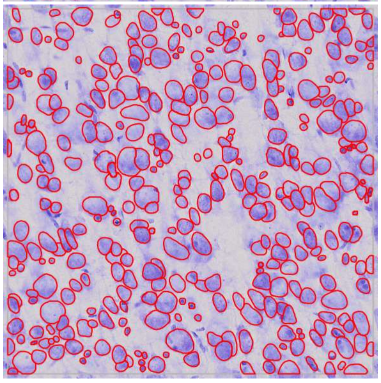

Region 5
Training

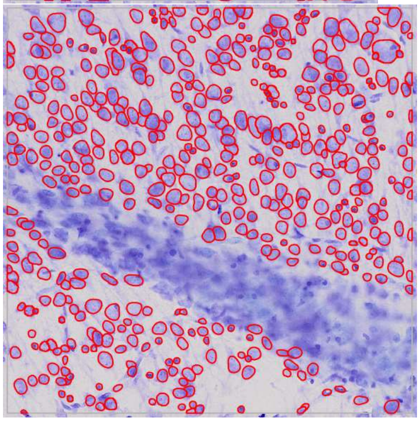

Region 6
Training

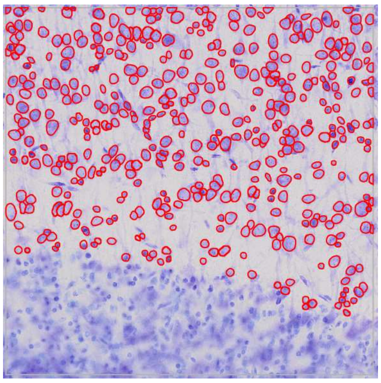

SLD_0000464.vsi - 20x_01 (5 training regions, 1 validation region; region area µm² 125535)

Region 1
Training

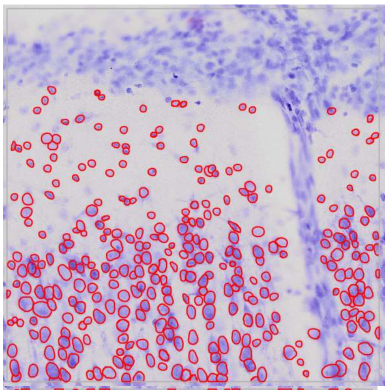

Region 2
Validation

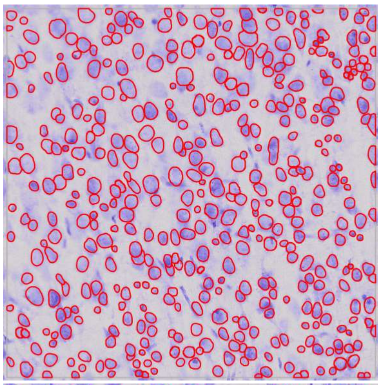

Region 3
Training

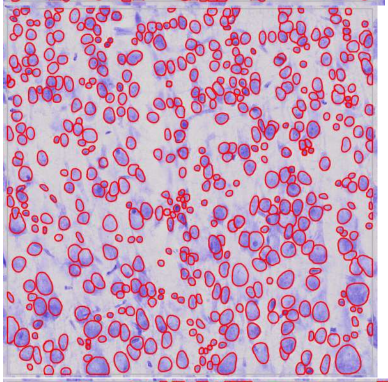

Region 4
Training

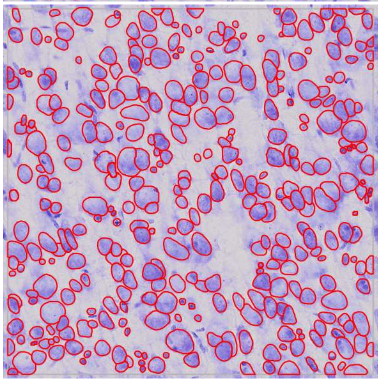

Region 5
Training

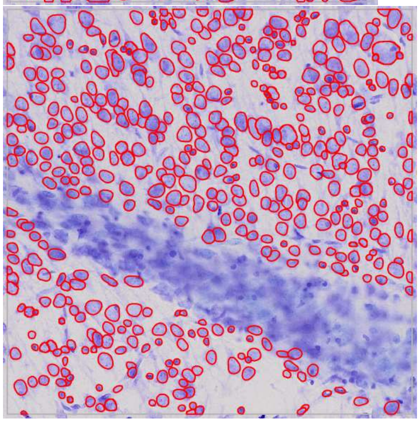

Region 6
Training

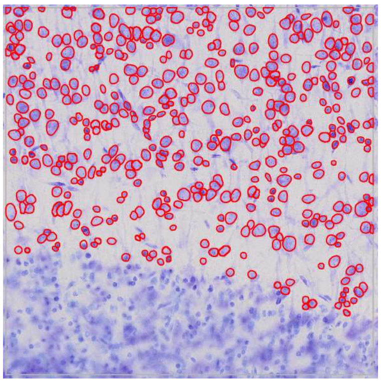

Cellpose - Version 1 - capillaries partially excluded 20240704 jm: Project: QuPath_Training_2_20220923.qpproj

- *v1_training result*
  - *model: cellpose_residual_on_style_on_concatenation_off_train_2021_12_13_11_14_32.300178*
  - *script:* `2. Run Cellpose V1 Model Prediction.groovy`

Scripts:

- '`2. Run Cellpose V1 Model Prediction.groovy`' (20211028, edited 2024 EPFL-PTBIOP-OlivierBurri)

Project folder:

- /LayerBoundariesProject/LBR_Algorithm/Cellpose/QuPath_Training_2_20220923/QuPath_Training_2_20220923.qpproj

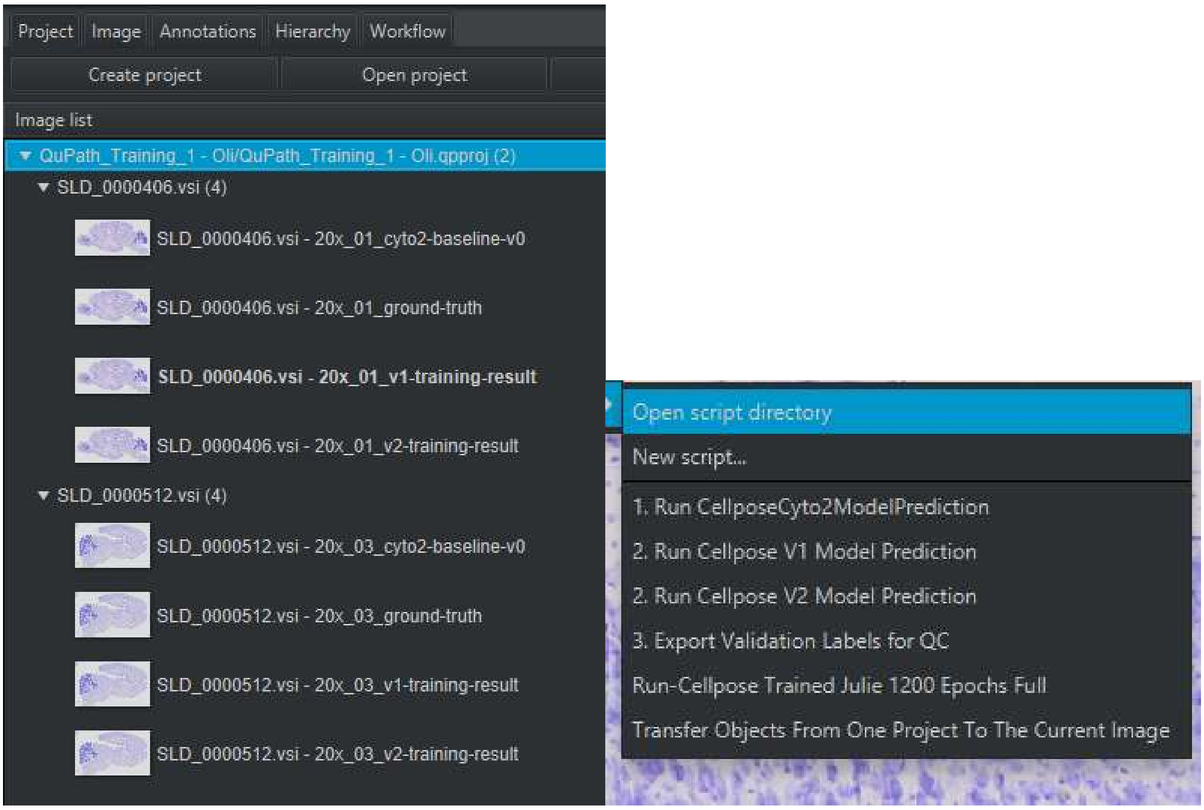

Goal: Multiple users (JulieMeystre, ClémentineLévy-Fidel, JoaoPrado) modified manually the segmentation on same images than version 0 and added squares

3 paradigms were tested (ie: "Training model"):

Training model 1: New annotations

Training model 2: New annotations, images downsampled

Training model 3: DBSCAN model

Images used for the 3 paradigms:

- SLD_0000406.vsi - 20x_01 (8 training regions; region area μm^2 31385)
- SLD_0000512.vsi - 20x_03 (7 training regions, 1 validation region; region area μm^2 31385)

Training notebook, Model, Scripts, Project Folder

<u>Training model 1: New annotations</u>

Parameters: Cellpose2D.builder (pathModel); epochs(1200); pixelSize(0,3460); TileSize(2048); Diameter(30)
Model:
`‘Cellpose_residual_on_style_on_concatenation_off_train_`**`2021_12_13`**`_11_14_32.300178’`
Script: `‘Run-Cellpose_Trained_Julie_1200_Epochs_Full.groovy’`
\2021_LayerBoundariesRefinement\LBR_Algorithm\Cellpose\QuPath_Cellpose_Training_20211213_v1\QuPath_Cellpose_Training_20211213_v1.qpproj\

Training model 2: New annotations, images downsampled

Parameters: Cellpose2D.builder (pathModel); epochs(1200); pixelSize(0,3460 * 2); TileSize(2048); Diameter(30)
Model:
`‘Cellpose_residual_on_style_on_concatenation_off_train_`**`2021_12_13`**`_11_14_32.300178’`
Script: `‘Run-Cellpose_Trained_Julie_1200_Epochs_Downsample_2.groovy’`
\2021_LayerBoundariesRefinement\LBR_Algorithm\Cellpose\QuPath_Cellpose_Training_20211213_v1\QuPath_Cellpose_Training_20211213_v1.qpproj

Training model 3: DBSCAN clustering

Parameters: Cellpose2D.builder (pathModel); epochs(1200); pixelSize(0,3460); TileSize(2048); Diameter(30); use DBScan Omnipose Clustering
Model:
`‘Cellpose_residual_on_style_on_concatenation_off_train_`**`2021_12_13`**`_11_14_32.300178’`
Script: `‘Run-Cellpose_Trained_Julie_1200_Epochs_Full_DBSCAN.groovy’`
\2021_LayerBoundariesRefinement\LBR_Algorithm\Cellpose\QuPath_Cellpose_Training_20211213_v1\QuPath_Cellpose_Training_20211213_v1.qpproj

Results

Training model 1: New annotations

Among the 3 paradigms, version 1 is the best cell segmentation 1. It is quite accurate; it detects all cells; It separates well clusters (but still some mistakes)
-> v1 could be improved with:

a. If a dark cell on top of another fainter, define only one cell (instead of 2)
b. Continue the cell contour at the training region border (for the algorithm not to be lost)
c. Discuss if capillaries should be included at the algorithm training step and then excluded at the analysis (it might be better to include them at segmentation and exclude them at analysis)
d. Go to -> v2

Training model 2: New annotations, images downsampled

Downsampled the images of 2. It misses a lot of cells (large and small) compared to the original pixel size and has difficulty separating cells cluster. **-> abort**

Training model 3: DBSCAN clustering

DBScan was trained for bacteria analysis (elongated cells and in cluster). On our datasets, it shows difficulties separating large cells and it detects weird cell shape. **-> abort**

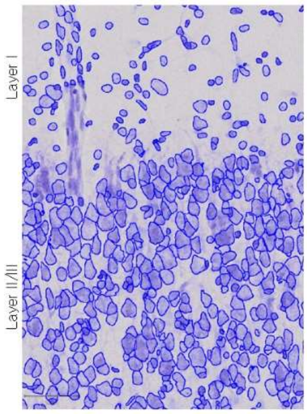

Training model 1
New annotations

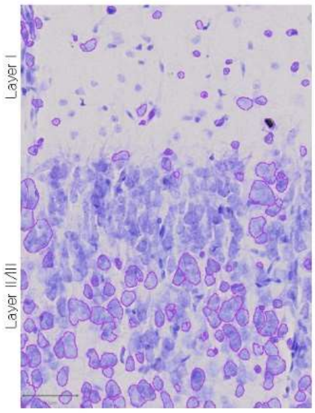

Training model 2
New annotations, images downsampled
ABORT

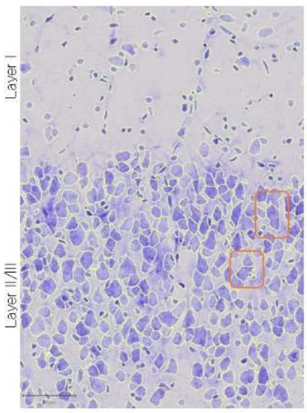

Training model 3
DBSCAN clustering
ABORT

Images detail

SLD_0000406.vsi - 20x_01 (8 training regions; region area µm2 31385)

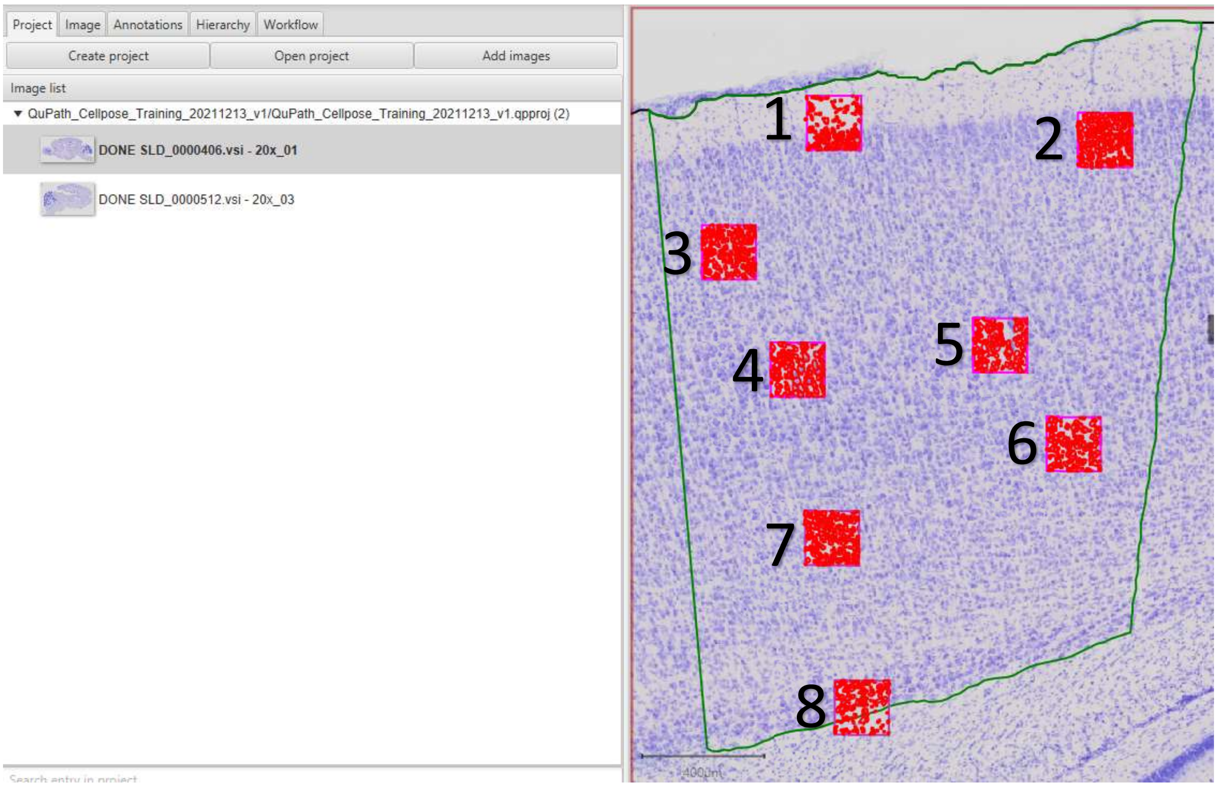

Region 1
Training

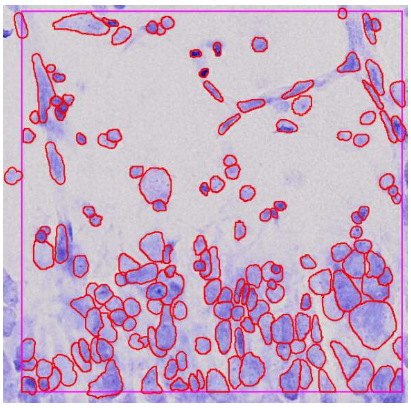

Region 2
Training

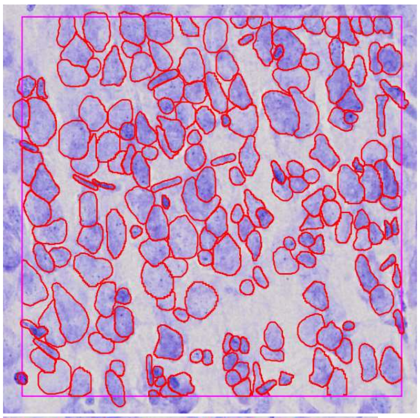

Region 3
Training

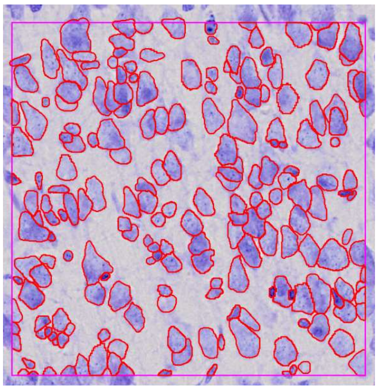

Region 4
Training

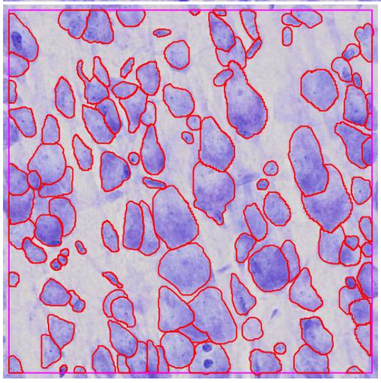

Region 5

Training

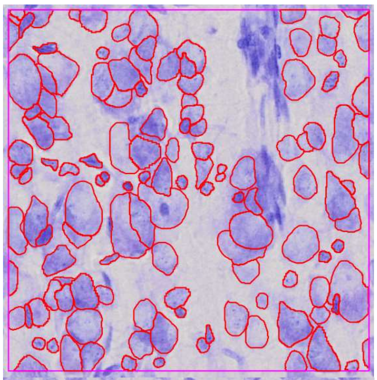

Region 6

Training

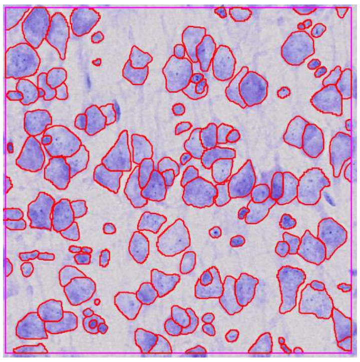

Region 7

Training

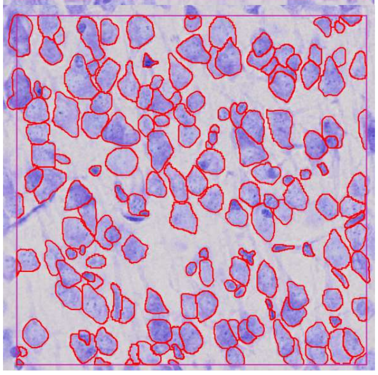

Region 8

Training

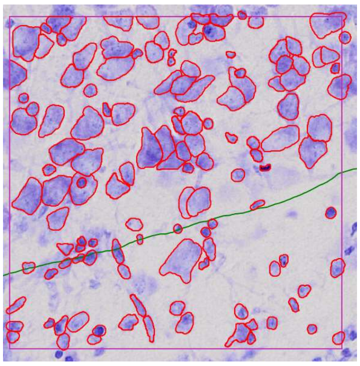

SLD_0000512.vsi - 20x_03 (7 training regions, 1 validation region; region area µm2 31385)

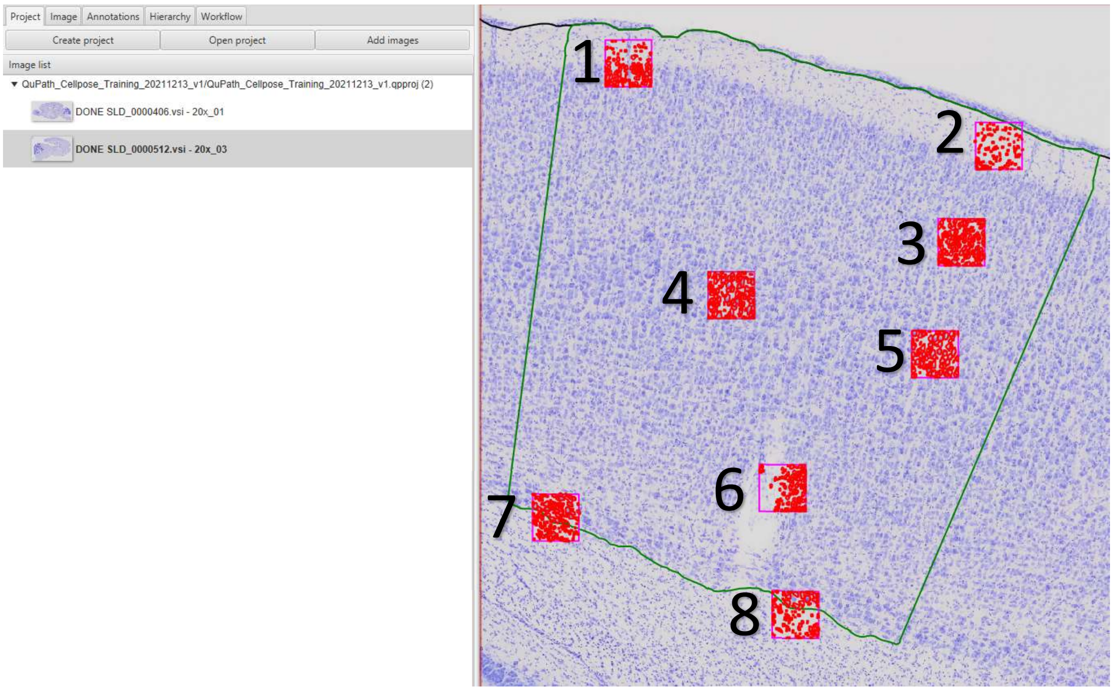

Region 1

Training

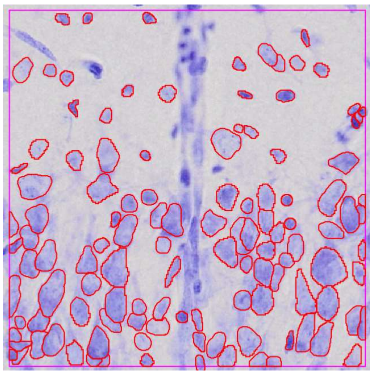

Region 2

Training

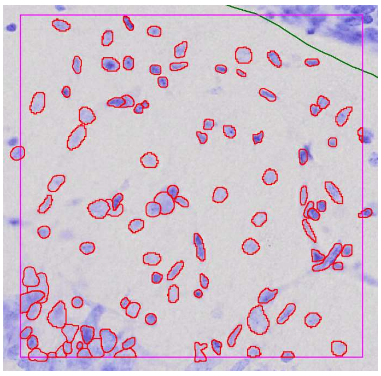

Region 3
Training

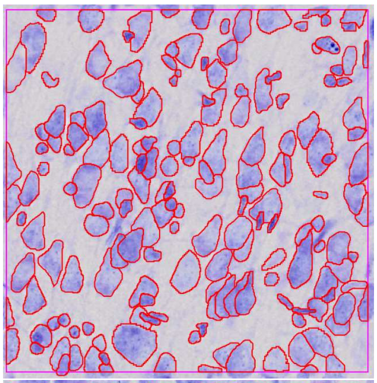

Region 4
Validation

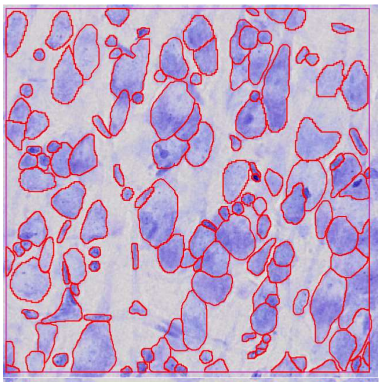

Region 5
Training

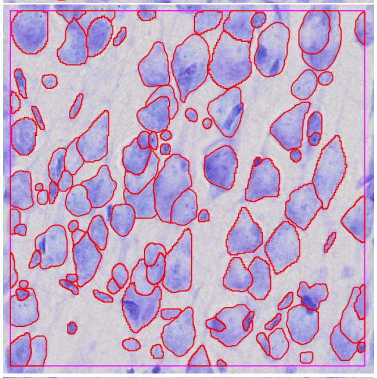

Region 6
Training

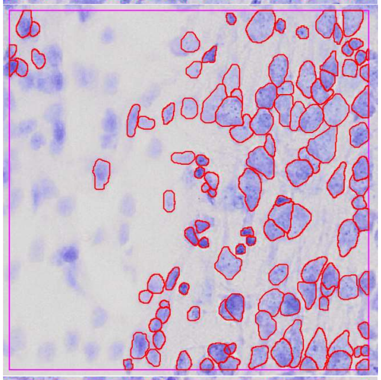

Region 7
Training

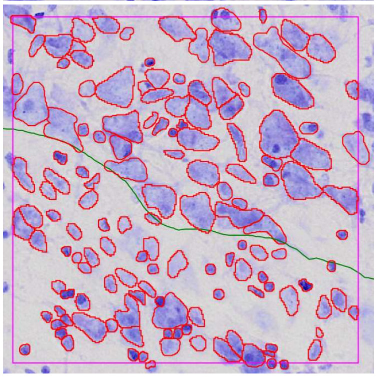

Region 8
Training

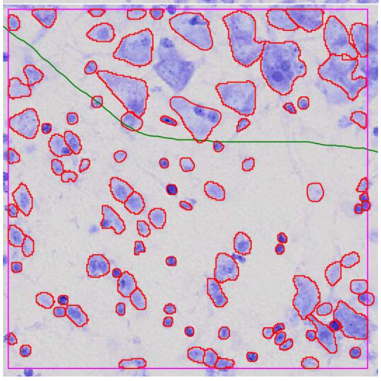

Comparison - version 0 vs version 1

V0 ('Cyto2')	V1 ('Julie Annotations')	V1 ('Julie Annotations Downsampled')	V1 ('Julie Annotations DBSCAN')

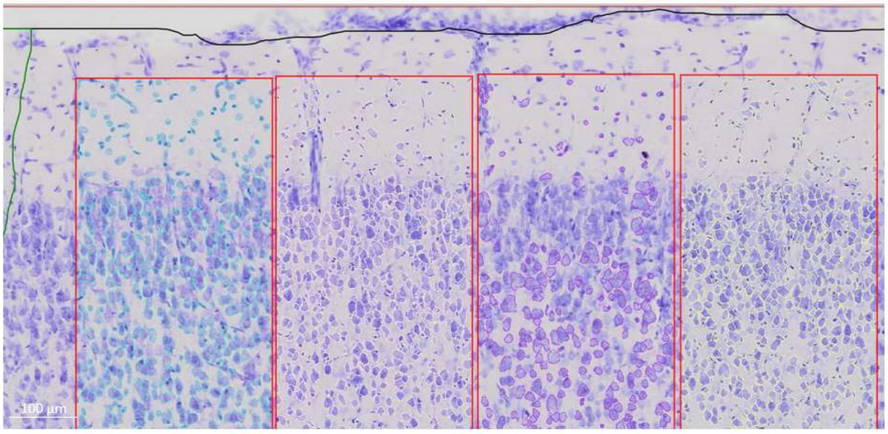

V0 ('Cyto2')	V1 ('Julie Annotations')	V1 ('Julie Annotations Downsampled')	V1 ('Julie Annotations DBSCAN')
It misses cells, especially big ones<br>Cells are round shape<br>Has difficulty separating cells cluster	It is quite accurate it detects all cells<br>It separates well clusters (some mistakes)	It misses a lot of cells (large and small) compared to the original pixel size<br>Has difficulty separating cells cluster	It has some difficulties separating large cells<br>It detects weird cell shape

QuPath_Cellpose_Training_20211213_v1.qpproj:

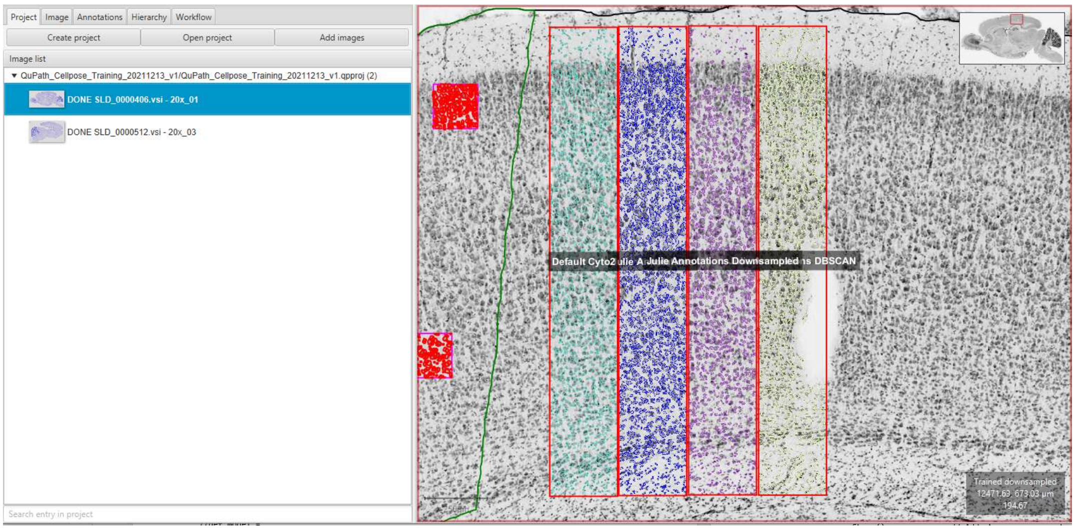

Cellpose - Version 2 – capillaries excluded

- *v2_training result*
  - *model: cellpose_residual_on_style_on_concatenation_off_train_2022_01_11_15_43_09.386142*
  - *script:* `2. Run Cellpose V2 Model Prediction.groovy`

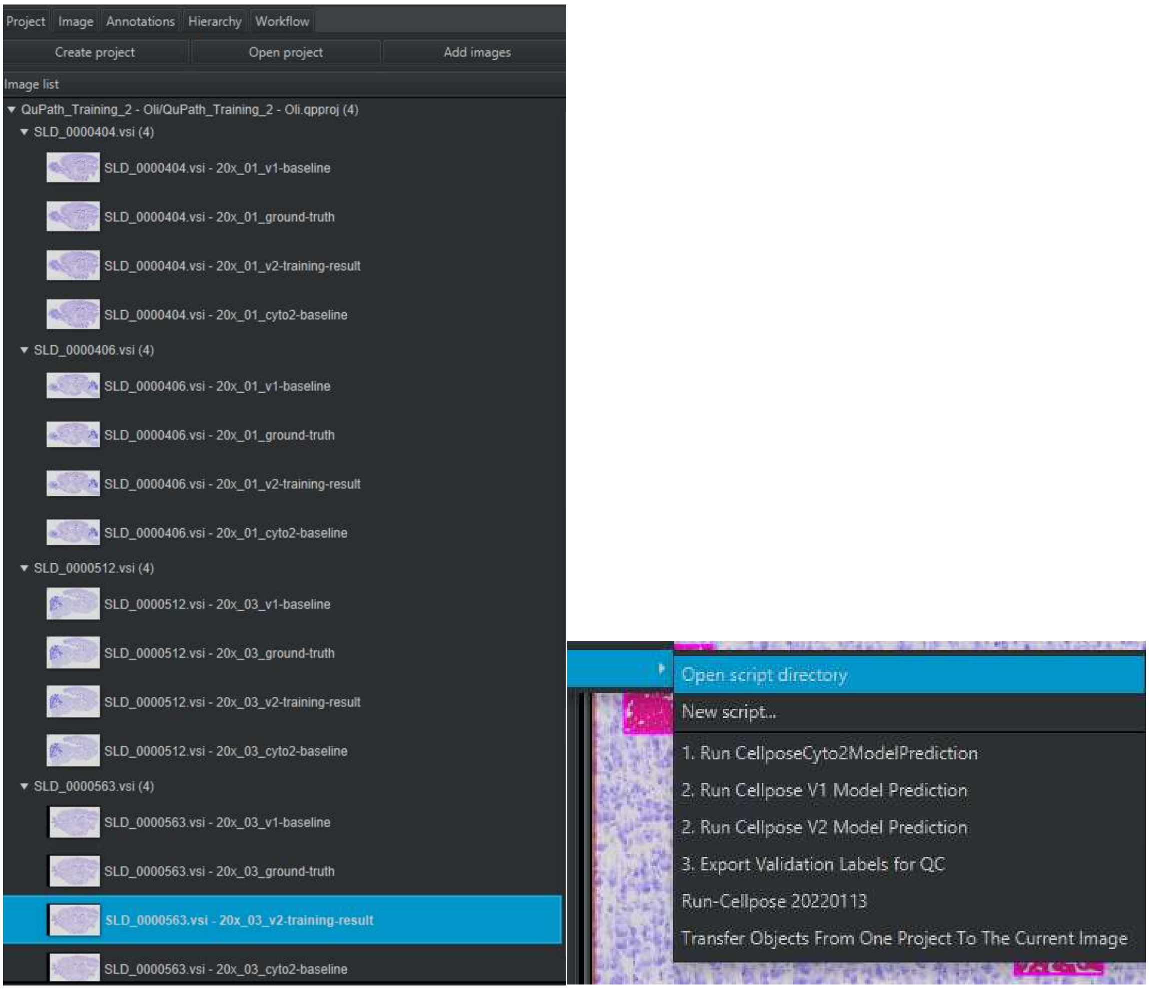

Training notebook, Model, Scripts, Project Folder

Parameters: pixelSize(0,3460); Diameter(30); CellExpansion (5,0); CellConstrainScale(1.5)

Model: `Cellpose_residual_on_style_on_concatenation_off_train_`**`2022_01_11`**`_15_43_09.386142`

Script: ‘`Run-Cellpose_20220113.groovy`’

Project folder:

- /LayerBoundariesProject/LBR_Algorithm/Cellpose/QuPath_Training_2_20220923/ QuPath_Training_2_20220923.qpproj

Results

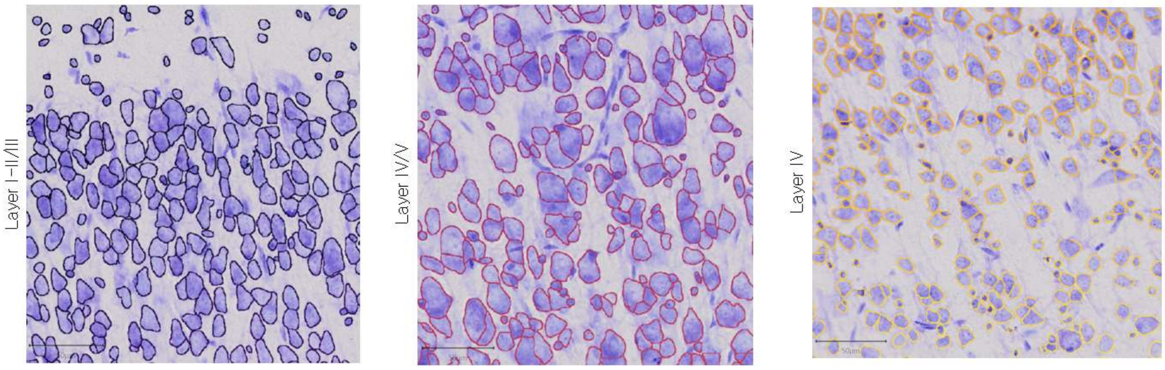

Images detail

SLD_0000406.vsi - 20x_01_JPR_v2_done

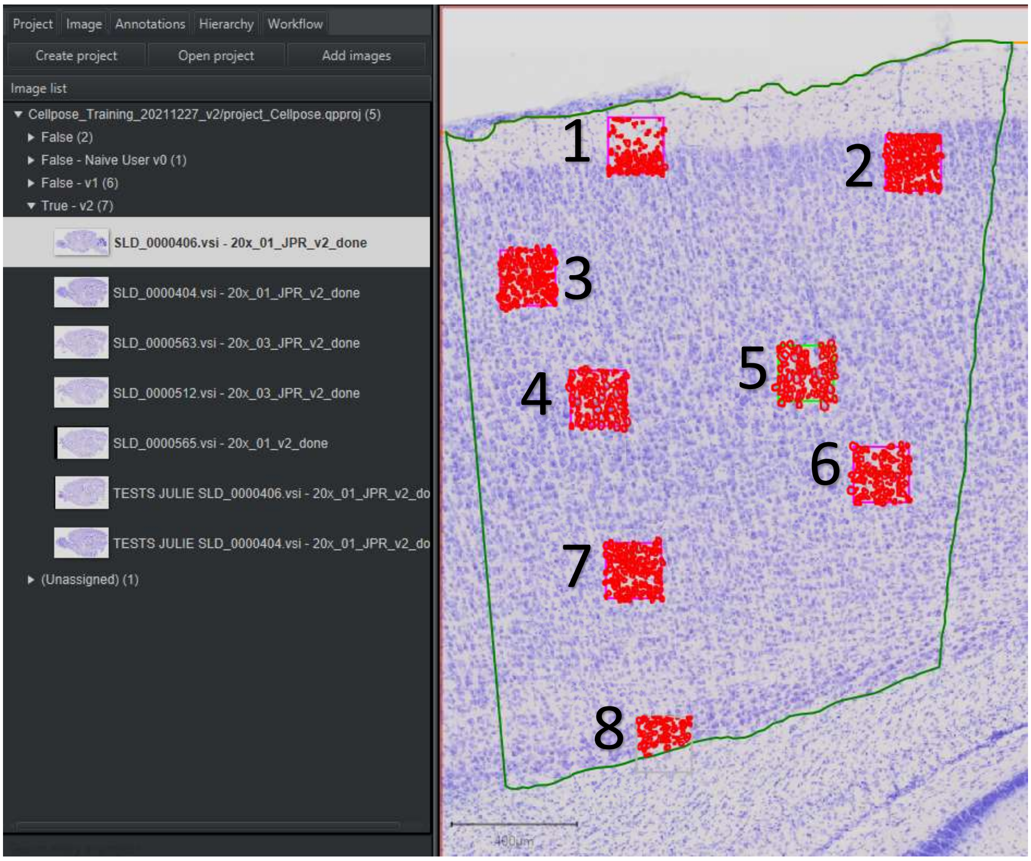

Region 1 Training

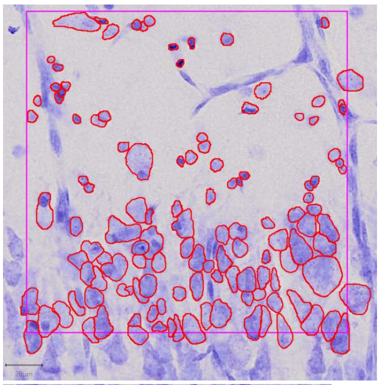

Region 2 Training

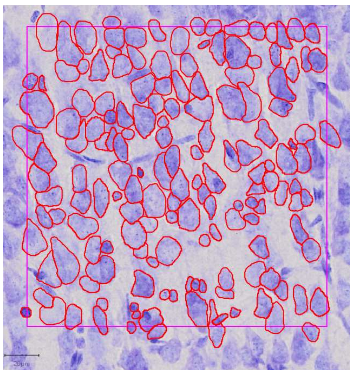

Region 3 Training

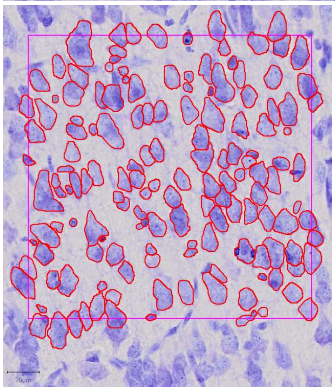

Region 4 Training

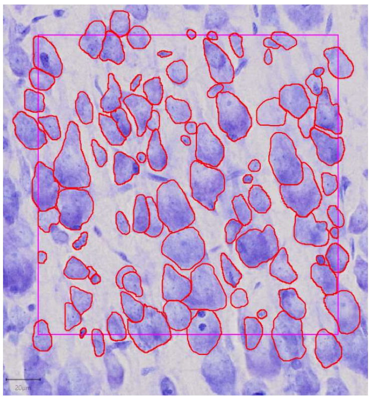

Region 5
Validation

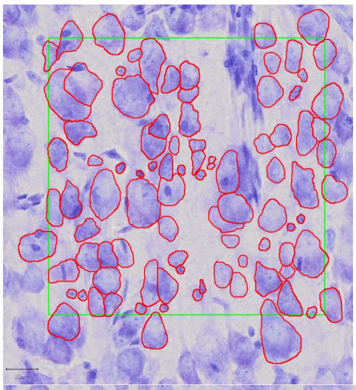

Region 6
Training

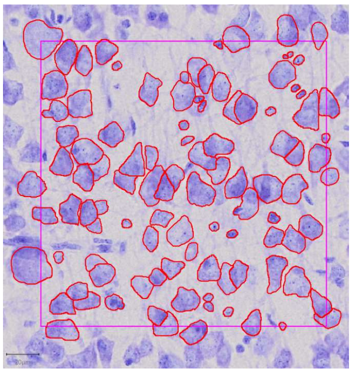

Region 7
Training

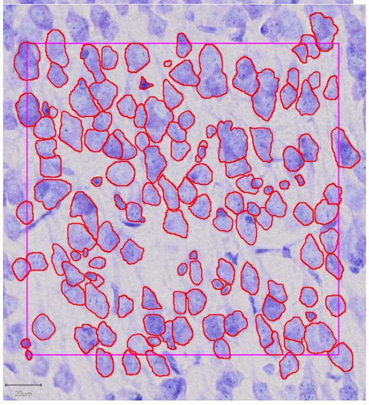

Region 8
Ignore

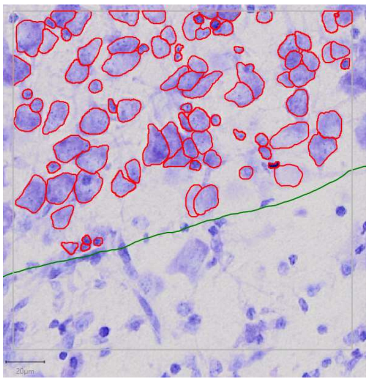

SLD_0000404.vsi - 20x_01_JPR_v2_done

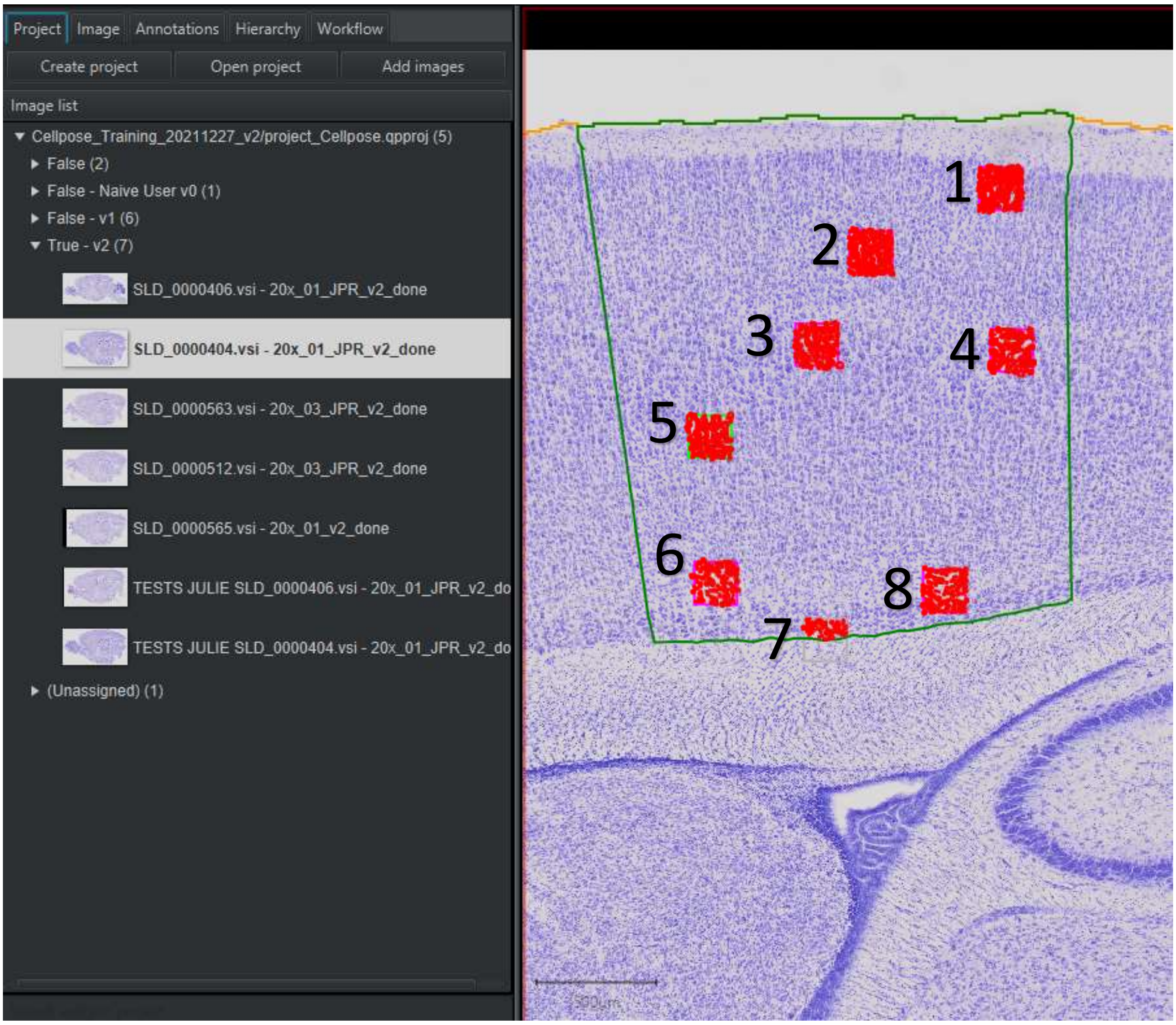

Region 1
Training

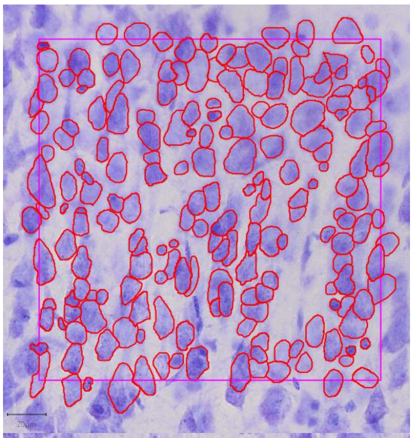

Region 2
Training

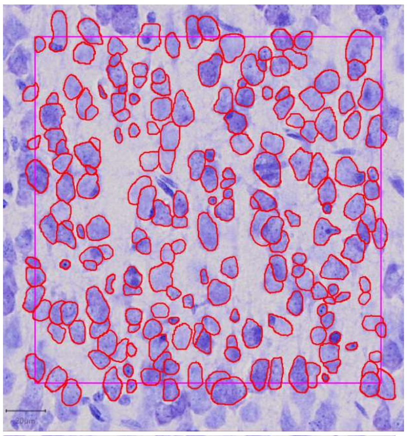

Region 3
Training

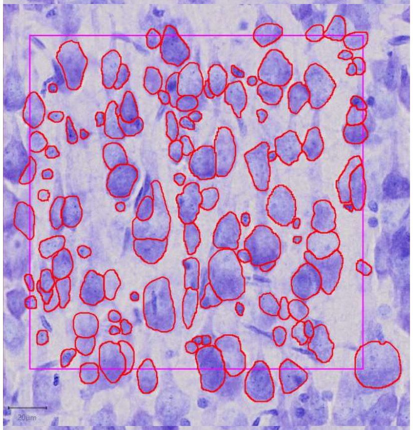

Region 4
Training

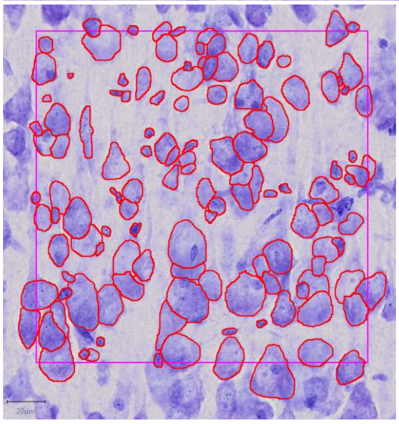

Region 5
Validation

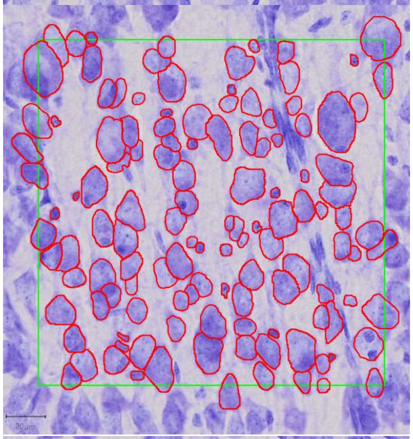

Region 6
Training

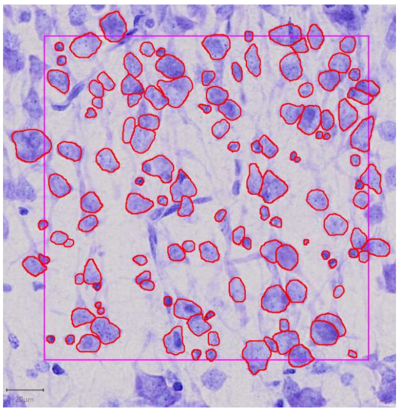

Region 7
Ignore

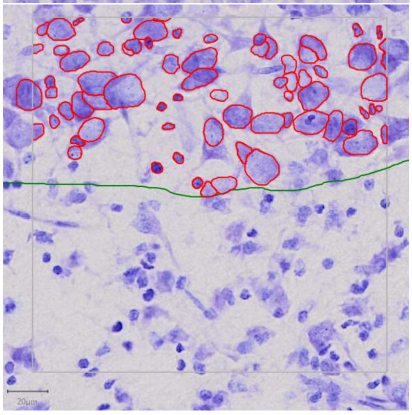

Region 8
Training

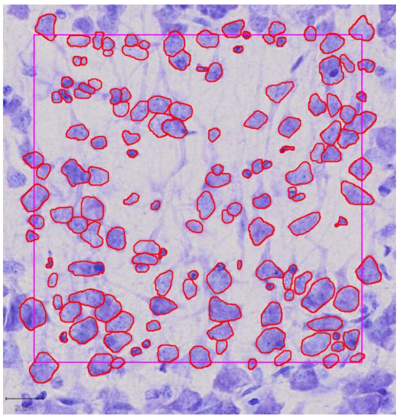

SLD_0000563.vsi - 20x_03_JPR_v2_done

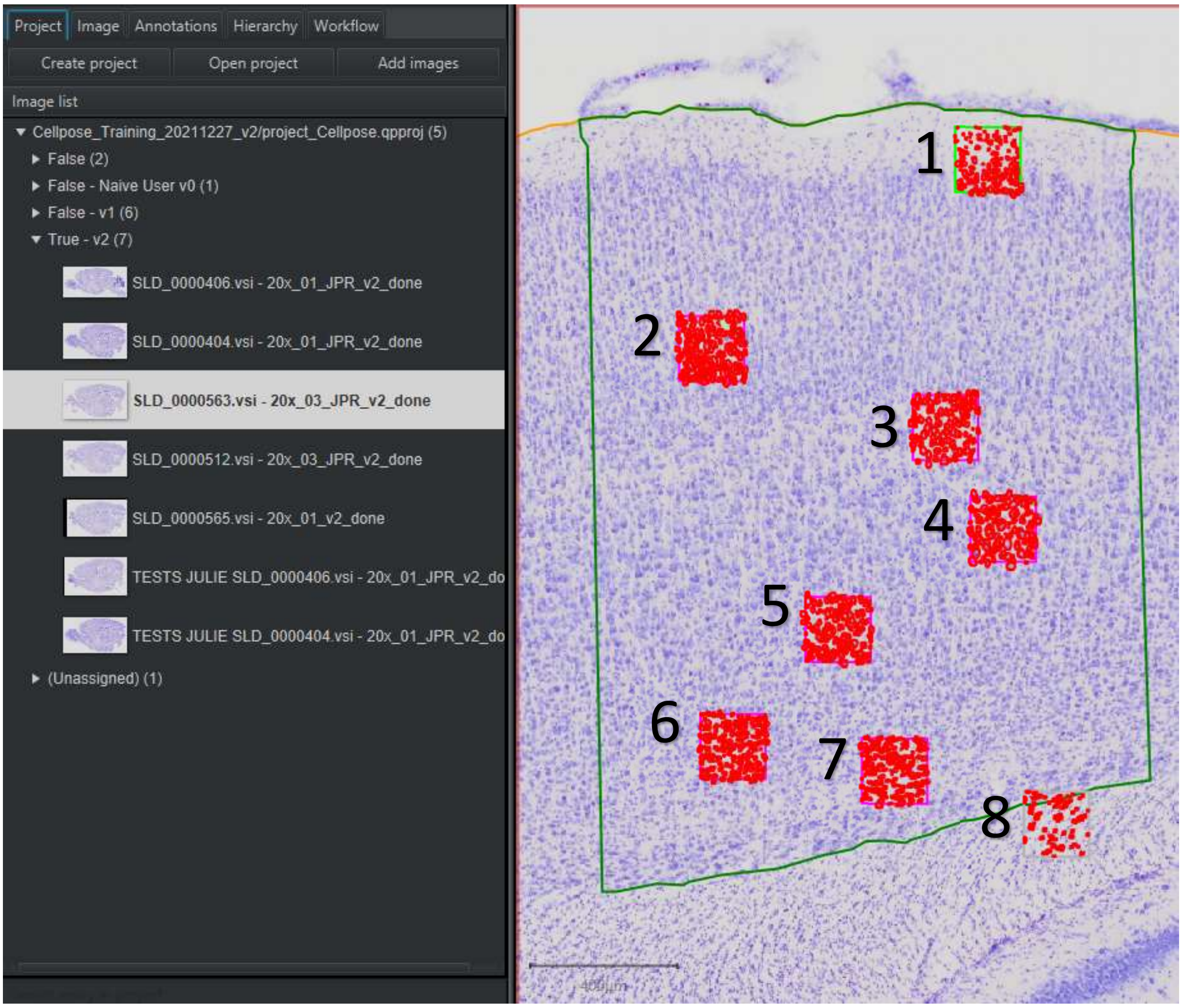

Region 1
Validation

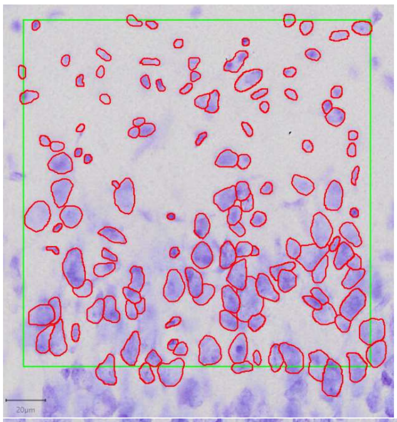

Region 2
Training

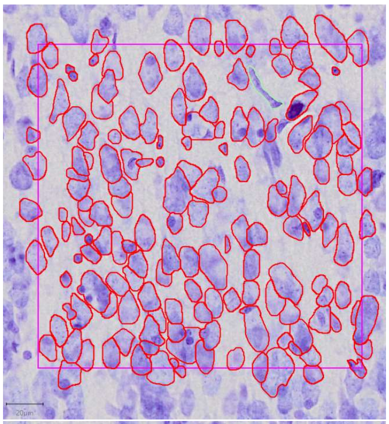

Region 3
Training

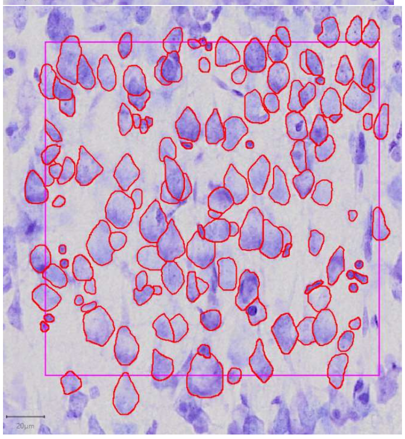

Region 4
Training

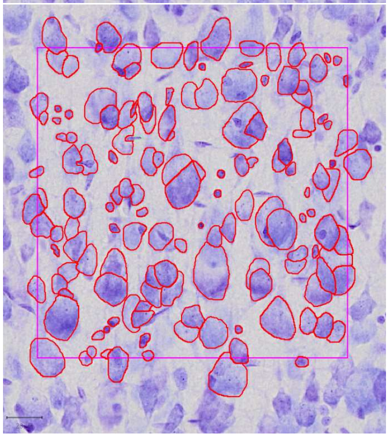

Region 5
Training

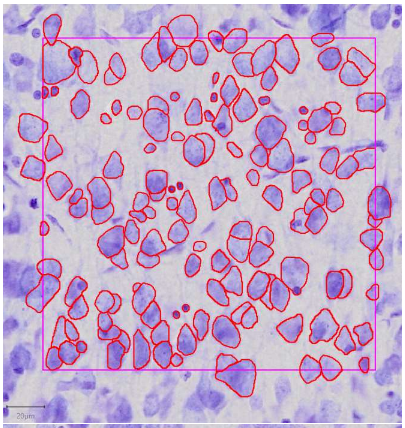

Region 6
Training

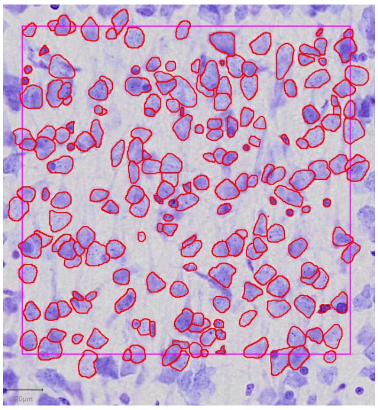

Region 7
Training

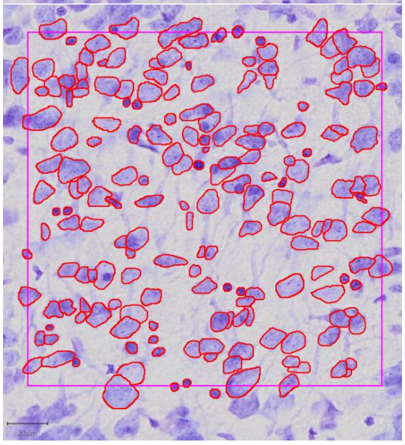

Region 8
Ignore

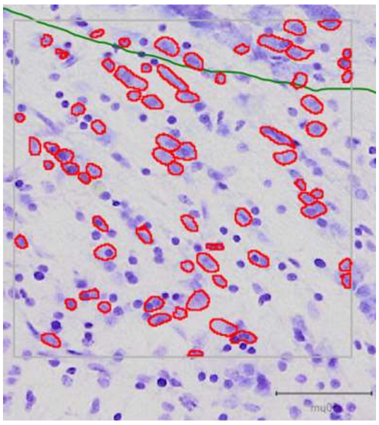

SLD_0000512.vsi - 20x_03_JPR_v2_done

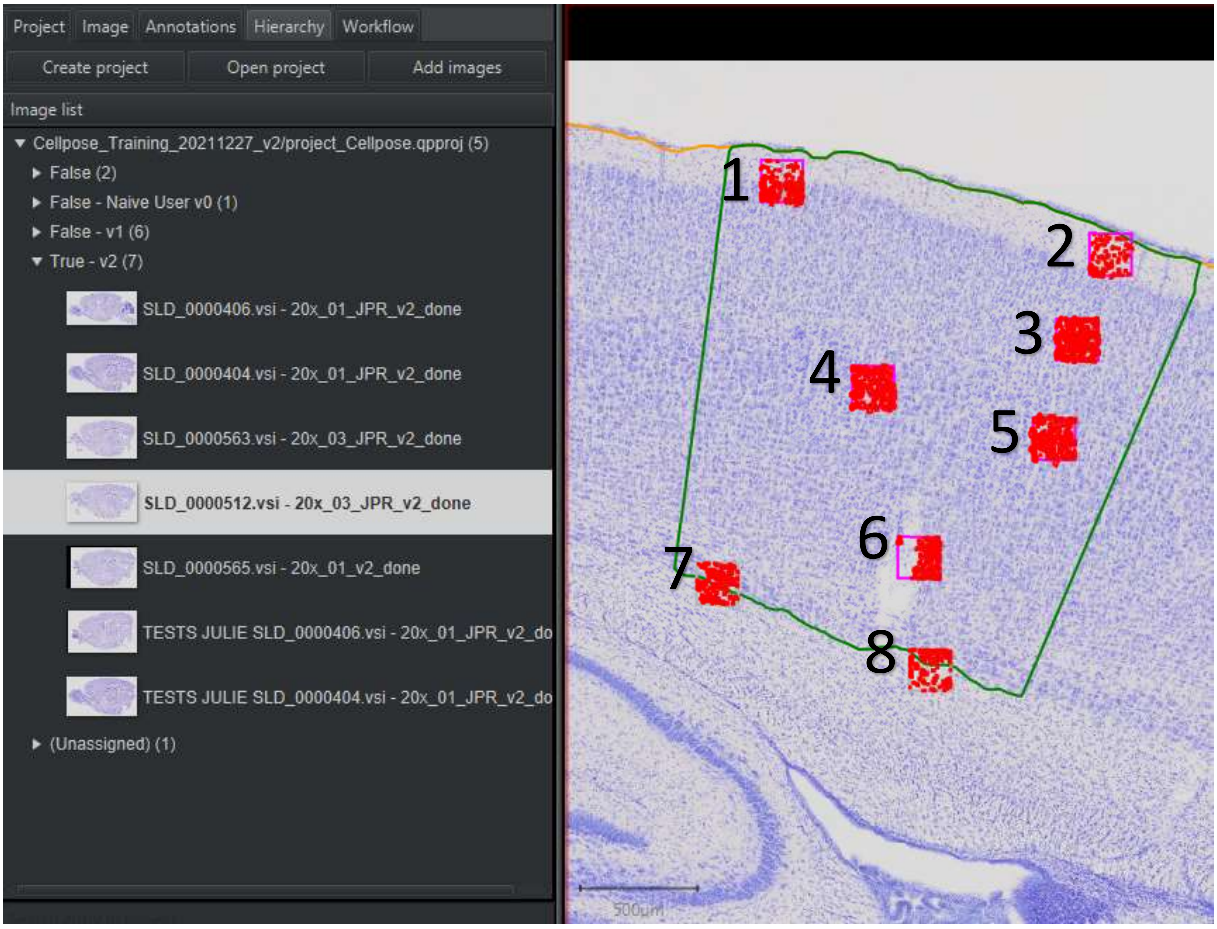

Region 1
Training

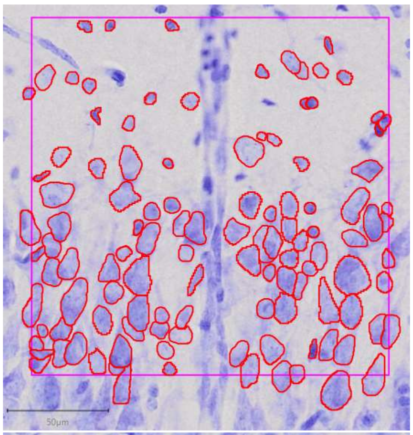

Region 2
Training

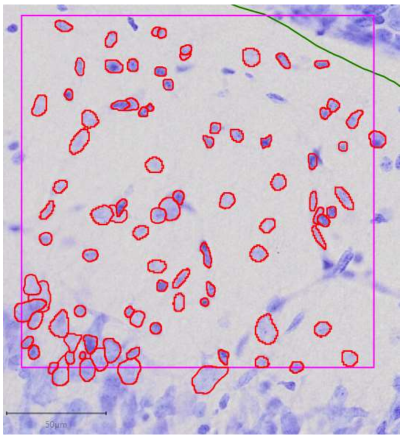

Region 3
Training

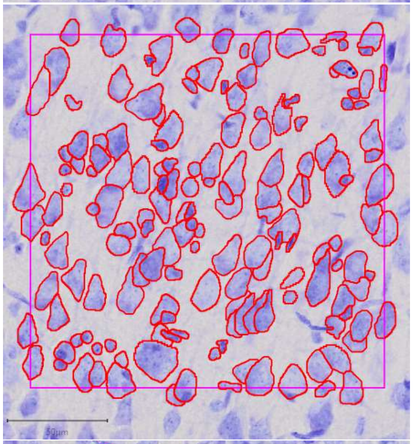

Region 4
Training

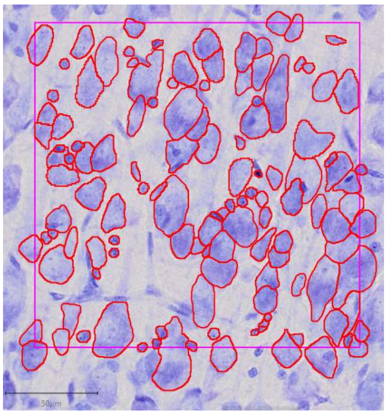

Region 5
Training

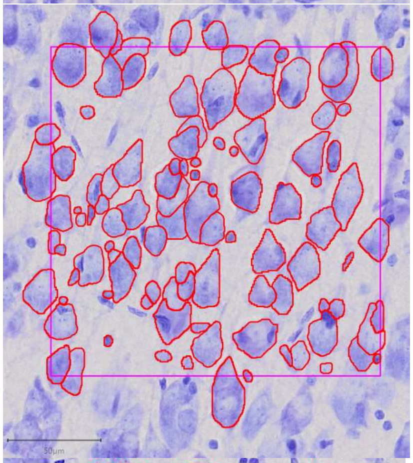

Region 6
Training

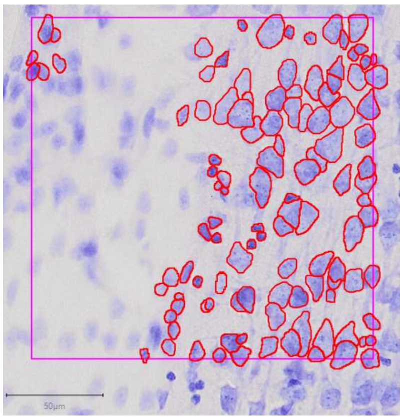

Region 7
Ignore

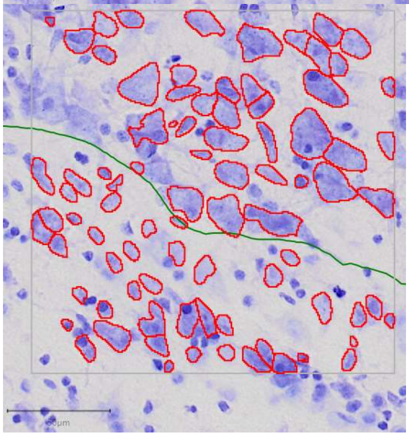

Region 8
Ignore

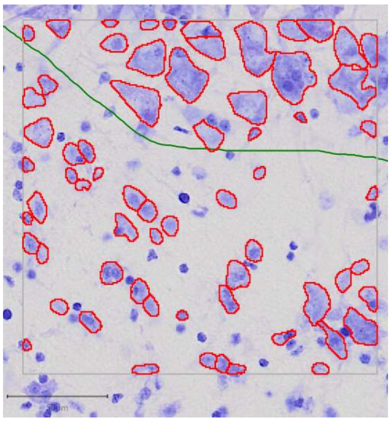

SLD_0000565.vsi - 20x_01_v2_done

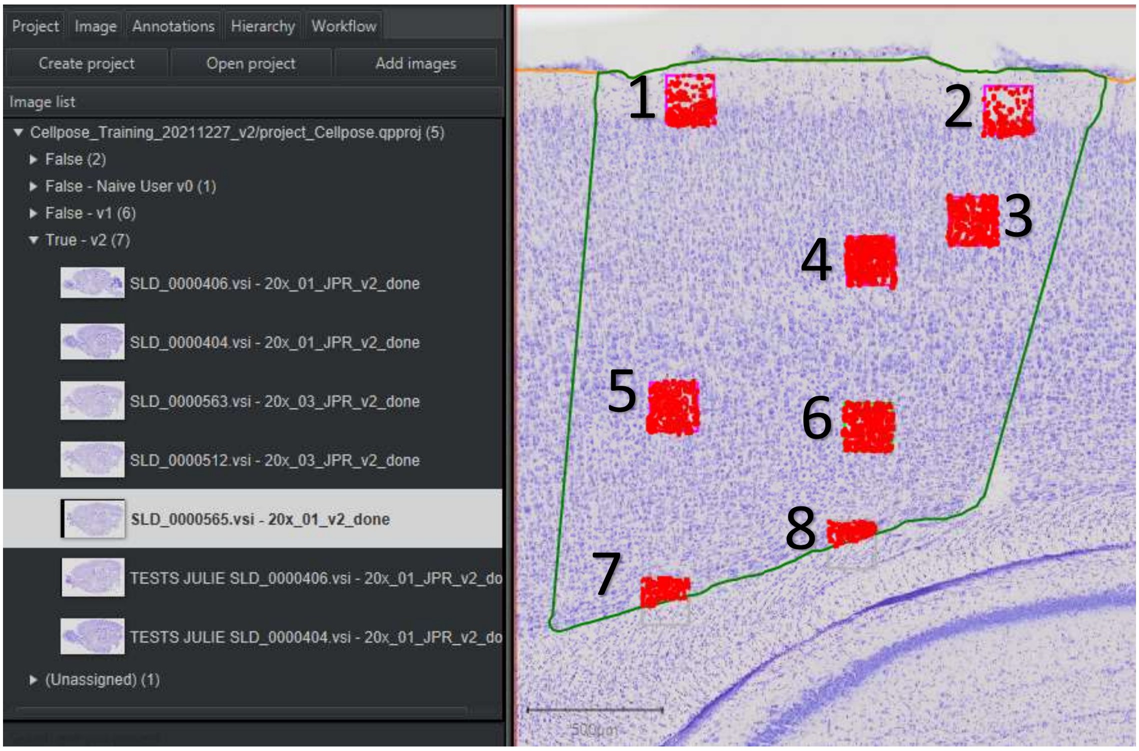

Region 1
Training

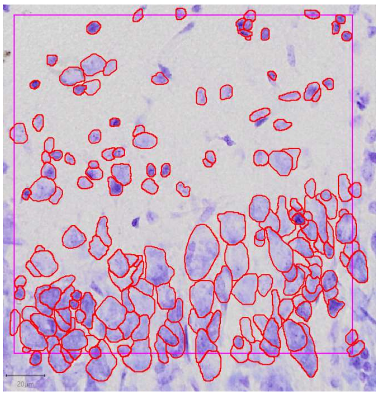

Region 2
Training

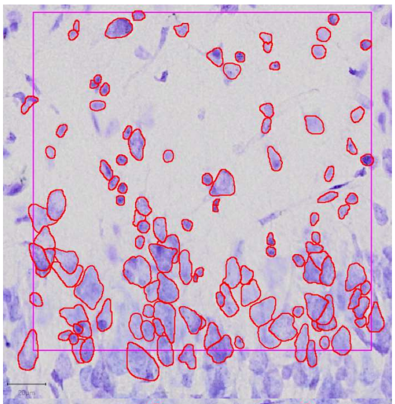

Region 3
Training

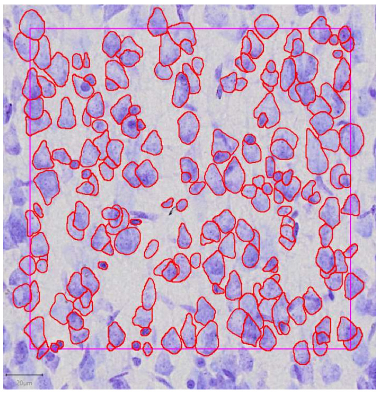

Region 4
Training

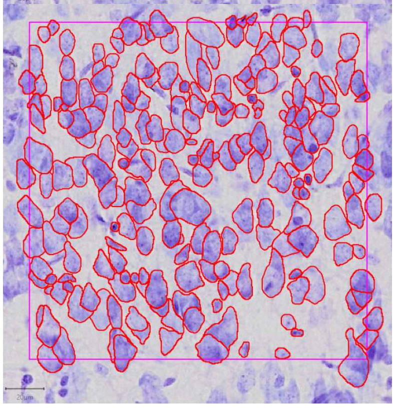

Region 5
Training

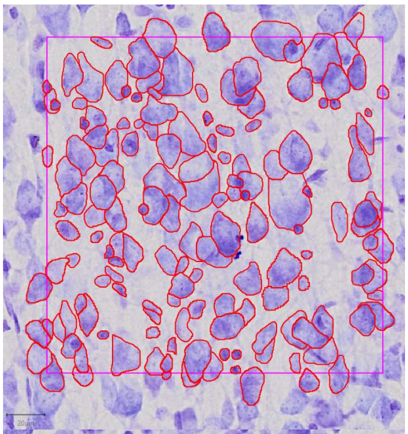

Region 6
Validation

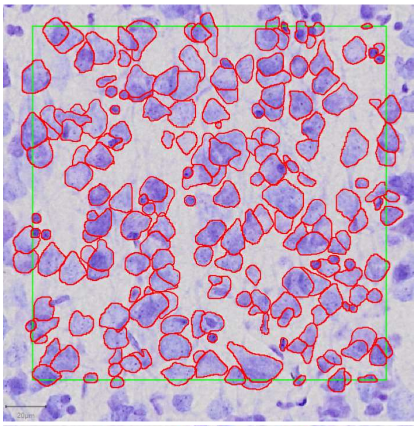

Region 7
Ignore

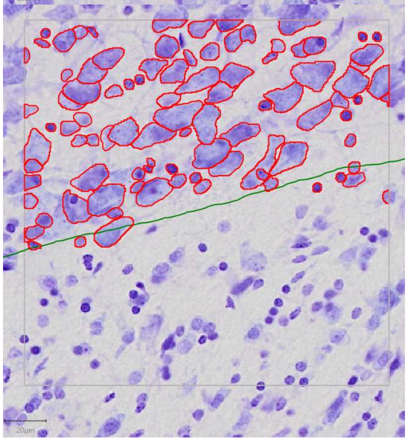

Region 8
Ignore

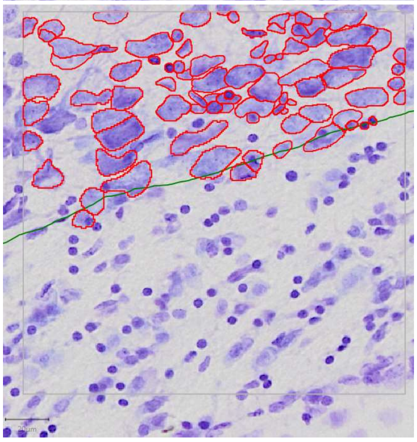

Comparison - version 1 vs version 2

RAW

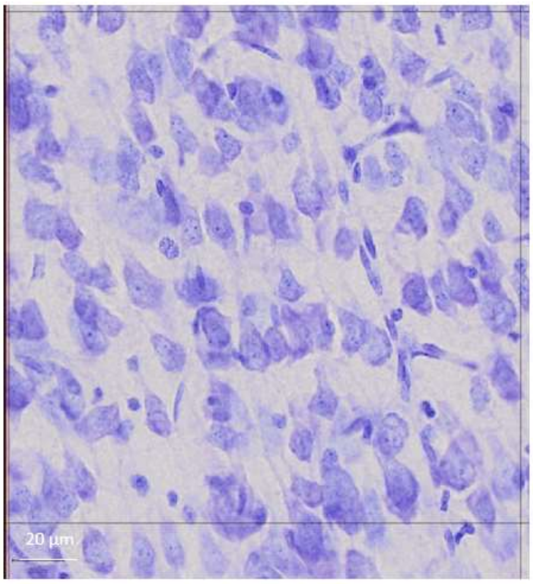

OUTPUT

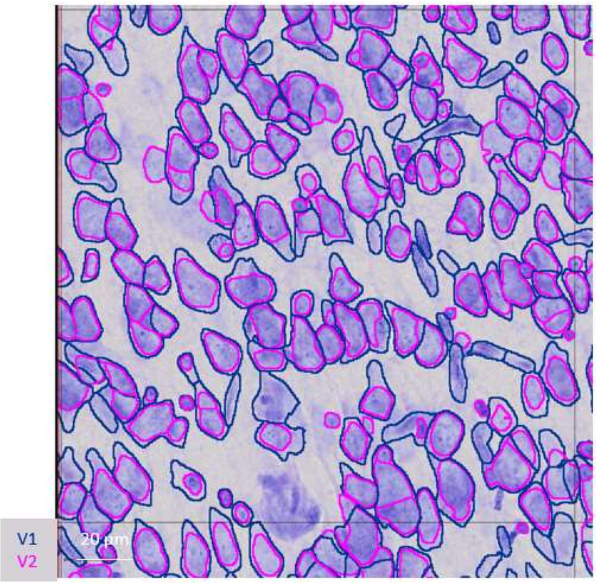

Cell segmentation - Cellpose

Please refer to Chapter “How to Setup and use the BIOP-QuPath-Cellpose extension v0.1”. This protocol describes all the steps needed to run cell segmentation on a given ROI in QuPath.

Data availability

Before starting, a QuPath project must be created. Regions of interest (ROI) and metadata must be completed.

QuPath objects to create (here for the 2021_Layer Boundaries Project)

Image pixel size is of 0,346 µm / pixel

- “SliceContour” ROI
- “S1HL” ROI
- “top_right” reference point (intersection of SliceContour and S1HL ROIs)
- “top_left” reference point (intersection of SliceContour and S1HL ROIs)
- “bottom_right” reference point (bottom right of S1HL ROIs)
- “bottom_left” reference point (bottom left of S1HL ROIs)
- “Distance to midline” metadata (according to the Paxinos Atlas (7th Ed.), lateral information in millimeter mm)
    - Empty (slice distance was not estimated because too far from the S1HL region)
    - around 2.40mm (slice distance estimated)
    - 2.40mm (slice mounting sequence is accurate)
- “Comment” metadata
    - Empty
    - broken slice
    - layer I is missing
    - …
- “Analyze” metadata
    - True
    - False

How to Setup and use the BIOP-QuPath-Cellpose extension v0.1

Date: 13 January 2022, Joao Prado

Context

This protocol is destined to users that wish to use BIOP's extension for QuPath-Cellpose integration.

Source of the extension: https://github.com/BIOP/qupath-extension-cellpose

Extension setup instuctions https://github.com/BIOP/qupath-extension-cellpose/blob/main/README.md

And for BIOP Instructions for cellpose installation

https://wiki-biop.epfl.ch/en/ipa/mamba#cellpose-fiji-cellpose-and-qupath-cellpose

Setup

1. Installation of conda:
   To see if conda is already installed, run in a command line (cmd.exe):
   - `conda list`

   a list of installed packages should appear if conda is already installed
   If not installed, follow https://docs.conda.io/projects/conda/en/4.6.1/user-guide/install/

2. Creation of a conda environment for Cellpose:
   Run in a command line (cmd.exe):
   - `conda create --name cellpose python=3.8`
   - `conda activate cellpose`
   - `python -m pip install cellpose`

3. Run Cellpose (in order to download pre-trained built-in models)
   Open a new terminal (cmd.exe) and run:
   - `conda activate cellpose`
   - `Cellpose`

   Cellpose may not download the built-in models by itself, depending on authorized protocols.
   One can check the download in the folder C:/yourusername/.Cellpose/models

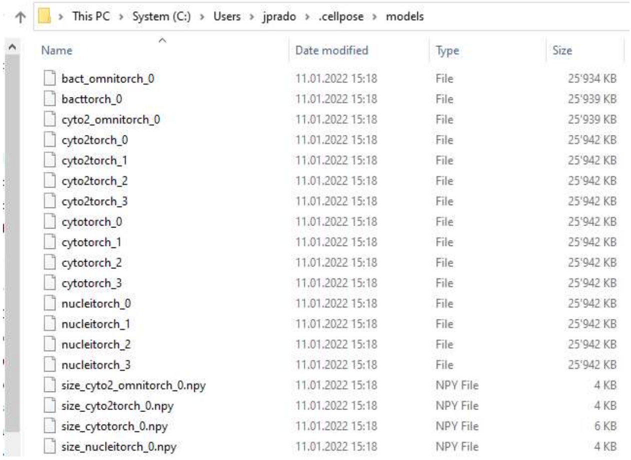

   If the folder is empty, the models can be downloaded from a Google Drive link available at https://github.com/MouseLand/cellpose

4. Download extension qupath-extension-Cellpose-[version].jar from Project Releases (https://github.com/biop/qupath-extension-cellpose/releases)
5. Drag & drop the file in an open QuPath project. Save and Quit QuPath.
6. Re-open QuPath and click in Edit -> Preferences -> Cellpose, input:
   a. Cellpose Environment Name: Cellpose

b. Cellpose Version: Omnipose after v0.7.2
c. Cellpose Environment Type: Anaconda or Miniconda

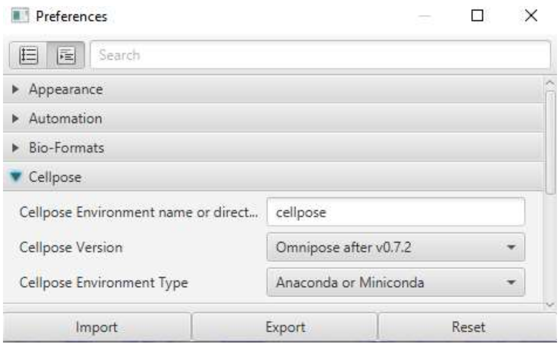

User Guide

Before start, the following scripts should be added to the "scripts" folder of the QuPath project:

" Run-Cellpose_20220113.groovy"

If missing, they can be found and copied from the project named "Cellpose_Training_20211227_v2"

Running cell identification with Cellpose

- Select the ROI :

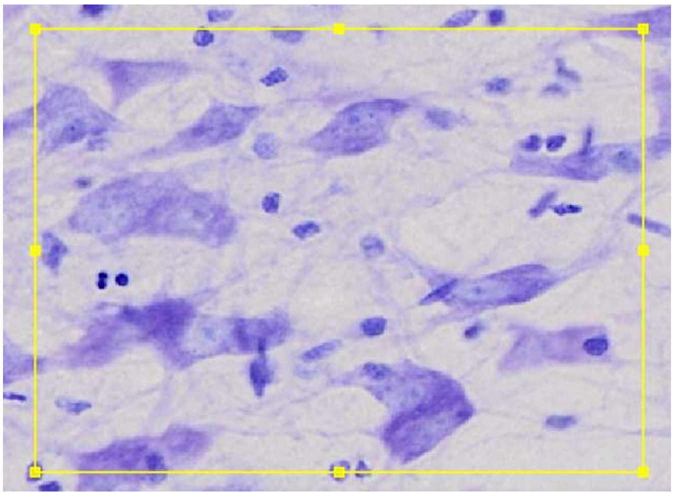

- Go to: Automate -> Project scripts… -> "Run-Cellpose_20220113"
- Go to: Run -> Run (or Control+R). If successfully finished, the command line outputs the following:

```
INFO: Saving images for 1 tiles
INFO: Saving to Z:\Training\Cellpose_Training_20211227_v2_old-BIOP\cellpose-temp\Temp_36371_13017.tif
INFO: Executing command: [cmd.exe /C conda activate cellpose & python -W ignore -m cellpose --dir Z:\Training\Cellpose_Training_20211227_v
INFO: cellpose: 2022-01-13 15:08:40,579 [INFO] WRITING LOG OUTPUT TO C:\Users\jprado\.cellpose\run.log
INFO: cellpose: 2022-01-13 15:08:42,342 [INFO] >>>> using CPU
INFO: cellpose: 2022-01-13 15:08:42,345 [WARNING] model path does not exist, using cyto model
INFO: cellpose: 2022-01-13 15:08:42,349 [INFO] >>>> running cellpose on 1 images using chan_to_seg GRAY and chan (opt) NONE
INFO: cellpose: 2022-01-13 15:08:42,349 [INFO] >>>> omni is 0, cluster is 1
INFO: cellpose: 2022-01-13 15:08:42,351 [WARNING] pretrained model has incorrect path
INFO: cellpose: 2022-01-13 15:08:42,351 [INFO] >>cyto<< model set to be used
INFO: cellpose: 2022-01-13 15:08:42,428 [INFO] >>>> using diameter 30.00 for all images
INFO: cellpose: 2022-01-13 15:08:42,430 [INFO] 0%|          | 0/1 [00:00<?, ?it/s]
INFO: cellpose: 2022-01-13 15:09:33,008 [INFO] 100%|##########| 1/1 [00:50<00:00, 50.58s/it]
INFO: cellpose: 2022-01-13 15:09:33,008 [INFO] 100%|##########| 1/1 [00:50<00:00, 50.58s/it]
INFO: cellpose: 2022-01-13 15:09:33,012 [INFO] >>>> completed in 50.670 sec
INFO: Virtual Environment Runner Finished
INFO: Cellpose command finished running
INFO: Getting objects for Z:\Training\Cellpose_Training_20211227_v2_old-BIOP\cellpose-temp\Temp_36371_13017_cp_masks.tif
INFO: Making measurements for Annotation (Rectangle)
INFO: Done!
```

- The detected region should look as follows:

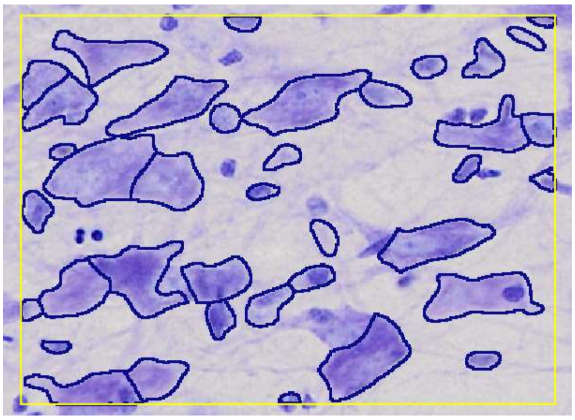

- The detected cells depend on the parameters chosen as input in the script for the Cellpose2D.builder:

```
def cellpose = Cellpose2D.builder(pathModel)
        .pixelSize(0.3460)              // Resolution for detection
        .diameter(30)                  // Average diameter of objects in px (at the requested pixel sie)
//        .cellExpansion(5.0)           // Approximate cells based upon nucleus expansion
//        .cellConstrainScale(1.5)     // Constrain cell expansion using nucleus size
        .measureShape()                // Add shape measurements
        .measureIntensity()            // Add cell measurements (in all compartments)
        .classify("Full")
        .useGPU()
        .build()
```

Two parameters are of special importance:

- .diameter( ): should be the mean size (in pixels) of the cells to be detected, an smaller diameter will help segmentation of smaller cells and hinder segmentation of bigger cells. This can be determined by exporting the ROI to ImageJ (Extensions -> ImageJ -> Send Region to ImageJ), and measuring cell diameter without scal. ImageJ can be downloaded here: (https://imagej.nih.gov/ij/download.html)
- .pixelSize( ): Size of a pixel in micrometers. (e.g. an image of 145.33 by 105.88 micrometers with 420 by 306 pixels should have 145.33/420 = 0.3460 micrometers as pixel size.

Training a new model (to be avoided if no GPU is available)

Requirements:

- The project contains at least one image with one locked Square ROI with class "Training": annotations must be hand drawn inside this ROI **with class None**.
- The project contains at least one image with one locked Squared ROI with class "Validation": annotations must be hand drawn inside this ROI **with class None**.
- The following scripts should be added to the "scripts" folder of the QuPath project: "Train-Cellpose-Model_20220110.groovy"
- If missing, this script can be found and re-copied from the folder named \2021_LayerBoundariesRefinement\LBR_Algorithm\Cellpose\Scripts

Ideally, for an efficient training:

- Each image in the project should contain multiple "Training" ROI and at least one "Validation" ROI. The Square ROI should be chosen randomly in the region being studied. If this region contains multiple layers or domains, the set of all "Validation" and "Training" ROIs should be a uniform sample of all layer/domains

Training procedure:

- Go to: Automate -> Project scripts… -> "Train-Cellpose-Model_20220110"

- Add the path of your QuPath project to .modelDirectory() parameter in line 13. (e.g. .modelDirectory(new File("Y:\\public\\julie.meystre_LNMC\\Your_QuPath_project\\models"))

```
1 import qupath.ext.biop.cellpose.Cellpose2D
2
3
4 // Example for training cellpose
5 // Requries a project, where there are annotations (usually rectangles) of class "Training" and "Validation" in which there are objects inside.
6 // The objects that you have annotated which will be exported as labeled images should have no PathClass at all.
7
8
9 def cellposeTrainer = Cellpose2D.builder('cyto2')
10 //        .channels("CY3 Laminin") // Up to two channels for training.
11         .pixelSize(0.6920)
12         .epochs(1500)
13         .modelDirectory(new File("Y:\\public\\julie.meystre_LNMC\\Cellpose_Training_20211227_v2\\models"))
14         .useGPU()
15         .build()
16
17
18 // train() will:
19 // 1. Go through the current project and save all "Training" and "Validation" regions into a temp folder (inside the current project)
20 // 2. Run the cellpose training via command line
21 // 3. Recover the model file after training, and copy it to where you defined in the builder, returning the reference to it
22
23 def resultModel = cellposeTrainer.train()
24
25 println "Model Saved under "+resultModel
26
```

- Change number of epochs according to the project needs in line 12. Be aware that increasing epochs will increase time of training.
- Run -> Run (or Control+R).
- When training is over, recover the output model's path from command line and input it in the cell identification script (line 5).
    - Save the modified segmentation script as a new script.
    - All backlashes "\" in a Windows path should be written as double backslashes "\\" in the script. Example: "Y:\public\julie.meystre_LNMC\Cellpose_Training_20211227_v2\models\Cellpose_resid"ual_on_style_on_concatenation_off_train_2022_01_11_15_43_09.386142
    - becomes
    - "Y:\\public\\julie.meystre_LNMC\\Cellpose_Training_20211227_v2\\models\\Cellpose_residual_on_style_on_concatenation_off_train_2022_01_11_15_43_09.386142"

Bibliography

(as of 13/01/2021)

Cellpose (Installation and user guide): https://github.com/MouseLand/Cellpose

QuPath: https://qupath.readthedocs.io/en/stable/index.html

Extension QuPath-Cellpose : https://github.com/BIOP/qupath-extension-Cellpose

Troubleshooting: https://forum.image.sc/, https://forum.image.sc/t/Cellpose-in-qupath-qupath-extension-Cellpose/58901

Addendum

(To be copy&pasted if .groovy files are lost, authorship: Olivier Burri, BIOP, EPFL)

Run StarDist.groovy

```
// Run StarDist Detection on images that contain the metadata key "Analyze" with value "True"
// Otherwise the image is skipped
// Olivier Burri, EPFL - PTECH - SV - BIOP
// For Julie Meystre, Markram Group
// Last Update: June 4th 2021
```

```
// Specify the model directory (you will need to change this!)
//def                                         model                                         =
'julie.meystre_LNMC\01413828_RH_Nissl_2_QuPath_StaDist_Training\Training_20210910'
def model = 'julie-1-slice_nissl_r16_p128_g2_k3_e400_se100_b32_aug'

// Start of Script
def pathModel = buildFilePath(PROJECT_BASE_DIR, "models", model)

def entry = getProjectEntry()

    println "Detecting Cells for $entry"
    def stardist = StarDist2D.builder(pathModel)
            .threshold(0.47)                // Probability (detection) threshold//
            .normalizePercentiles(1, 99.8)  // Percentile normalization
            .pixelSize(0.6920)              // Resolution for detection
            .ignoreCellOverlaps(false)      // Set to true if you don't care if cells expand
into one another
            .measureShape()                 // Add shape measurements
            .measureIntensity()             // Add cell measurements (in all compartments)
            .includeProbability(true)         // Add probability as a measurement (enables
later filtering)
            .simplify(1)                  // Control how polygons are 'simplified' to remove
unnecessary vertices
            .doLog()                        // Use this to log a bit more information while
running the script
            .build()

    // Run detection for the selected objects
    def imageData = getCurrentImageData()
    def pathObjects = getObjects{ it.getPathClass().equals( getPathClass( "Training" ) ) }

    stardist.detectObjects(imageData, pathObjects)

    println 'Done!'

    getDetectionObjects().each{ it.setPathClass( getPathClass("StarDist") ) }

// Imports
import qupath.tensorflow.stardist.StarDist2D
```

Convert StarDist Detections to Annotations.groovy

```
// NEED A SELECTED RECTANGLE IN YOUR IMAGE that has StarDist Detections on it.
def pathObjects = getObjects{ it.getPathClass().equals( getPathClass( "Training" ) ) }

pathObjects.each{ object ->


    insertObjects(object)

    def annots = object.getChildObjects().collect{ cell ->
        return PathObjects.createAnnotationObject( cell.getROI() )
    }

    println(annots)
    object.clearPathObjects()

    addObjects(annots)
}
fireHierarchyUpdate()
```

Run-Cellpose_Default_cyto2.groovy

```
import qupath.ext.biop.Cellpose.Cellpose2D

def pathModel = 'cyto2'
def Cellpose = Cellpose2D.builder(pathModel)
        .pixelSize(0.3460)
        .tileSize(2048)
```

```
        .diameter(30)                // Average diameter of objects in px (at the requested
pixel sie)
        .measureShape()              // Add shape measurements
        .measureIntensity()          // Add cell measurements (in all compartments)
        .classify("Cellpose cyto2")
        .build()

// Run detection for the selected objects
def imageData = getCurrentImageData()
def pathObjects = getSelectedObjects()
if (pathObjects.isEmpty()) {
    Dialogs.showErrorMessage("Cellpose", "Please select a parent object!")
    return
}
Cellpose.detectObjects(imageData, pathObjects)
println 'Done!'
```

Run-Cellpose_Trained_Julie_1200_Epochs_Full.groovy

```
import qupath.ext.biop.Cellpose.Cellpose2D

def            pathModel            =            'E:\\Julie\\Cellpose            Training
20211209\\models\\Cellpose_residual_on_style_on_concatenation_off_train_2021_12_13_11_14_32
.300178'
// Model trained on full size image, with cyto2 model as base for 1200 epochs
def Cellpose = Cellpose2D.builder(pathModel)
        .pixelSize(0.3460)
        .tileSize(2048)
        .diameter(30)                // Average diameter of objects in px (at the requested
pixel size)
        .measureShape()              // Add shape measurements
        .measureIntensity()          // Add cell measurements (in all compartments)
        .classify("Cellpose Julie Full")
        .build()

// Run detection for the selected objects
def imageData = getCurrentImageData()
def pathObjects = getSelectedObjects()
if (pathObjects.isEmpty()) {
    Dialogs.showErrorMessage("Cellpose", "Please select a parent object!")
    return
}
Cellpose.detectObjects(imageData, pathObjects)
println 'Done!'
```

Run-Cellpose_Trained_Julie_1200_Epochs_Downsample_2.groovy

```
import qupath.ext.biop.Cellpose.Cellpose2D

def            pathModel            =            'E:\\Julie\\Cellpose            Training
20211209\\models\\Cellpose_residual_on_style_on_concatenation_off_train_2021_12_13_11_14_32
.300178'
// Model trained on full size image, with cyto2 model as base for 1200 epochs
def Cellpose = Cellpose2D.builder(pathModel)
        .pixelSize(0.3460 * 2)
        .tileSize(2048)
        .diameter(30)                // Average diameter of objects in px (at the requested
pixel sie)
        .measureShape()              // Add shape measurements
        .measureIntensity()          // Add cell measurements (in all compartments)
        .classify("Cellpose Julie Downsample")
        .build()

// Run detection for the selected objects
def imageData = getCurrentImageData()
def pathObjects = getSelectedObjects()
if (pathObjects.isEmpty()) {
    Dialogs.showErrorMessage("Cellpose", "Please select a parent object!")
    return
```

```
}
Cellpose.detectObjects(imageData, pathObjects)
println 'Done!'
```

Run-Cellpose_Trained_Julie_1200_Epochs_Full_DBSCAN.groovy

```
import qupath.ext.biop.Cellpose.Cellpose2D

def             pathModel            =            'E:\\Julie\\Cellpose            Training
20211209\\models\\Cellpose_residual_on_style_on_concatenation_off_train_2021_12_13_11_14_32
.300178'
// Model trained on full size image, with cyto2 model as base for 1200 epochs

def Cellpose = Cellpose2D.builder(pathModel)
        .pixelSize(0.3460)
        .tileSize(2048)
        .diameter(30)                // Average diameter of objects in px (at the requested
pixel sie)
        .measureShape()              // Add shape measurements
        .measureIntensity()          // Add cell measurements (in all compartments)
              .clusterDBSCAN()                    // use DBScan Omnipose Clustering
        .classify("Cellpose Julie Full DBSCAN")
        .build()

// Run detection for the selected objects
def imageData = getCurrentImageData()
def pathObjects = getSelectedObjects()
if (pathObjects.isEmpty()) {
    Dialogs.showErrorMessage("Cellpose", "Please select a parent object!")
    return
}
Cellpose.detectObjects(imageData, pathObjects)
println 'Done!'
```

Run-Cellpose_20220113.groovy

import qupath.ext.biop.Cellpose.Cellpose2D

```
// Specify the model name (cyto, nuc, cyto2 or a path to your custom model)
// Full res model
def                                     pathModel                                     =
'Y:\\public\\julie.meystre_LNMC\\Cellpose_Training_20211227_v2\\models\\Cellpose_residual_o
n_style_on_concatenation_off_train_2022_01_11_15_43_09.386142'

// Half res model
//def                                    pathModel                                    =
'Y:\\public\\julie.meystre_LNMC\\Cellpose_Training_20211227_v2\\models\\Cellpose_residual_o
n_style_on_concatenation_off_train_2022_01_11_16_14_20.764792'
def Cellpose = Cellpose2D.builder(pathModel)
        .pixelSize(0.3460)              // Resolution for detection
        .diameter(30)                // Average diameter of objects in px (at the requested
pixel sie)
//        .cellExpansion(5.0)          // Approximate cells based upon nucleus expansion
//       .cellConstrainScale(1.5)     // Constrain cell expansion using nucleus size
        .measureShape()              // Add shape measurements
        .measureIntensity()          // Add cell measurements (in all compartments)
        .classify("Full")
        .useGPU()
        .build()

// Run detection for the selected objects
def imageData = getCurrentImageData()
def pathObjects = getSelectedObjects()
if (pathObjects.isEmpty()) {
    Dialogs.showErrorMessage("Cellpose", "Please select a parent object!")
    return
```

```
}
Cellpose.detectObjects(imageData, pathObjects)
println 'Done!'
```

Train-Cellpose-Model_202220110.groovy

import qupath.ext.biop.Cellpose.Cellpose2D

```
// Example for training Cellpose
// Requries a project, where there are annotations (usually rectangles) of class "Training"
and "Validation" in which there are objects inside.
// The objects that you have annotated which will be exported as labeled images should have
no PathClass at all.

def CellposeTrainer = Cellpose2D.builder('cyto2')
//        .channels("CY3 Laminin") // Up to two channels for training.
        .pixelSize(0.6920)
        .epochs(1500)
        .modelDirectory(new
File("Y:\\public\\julie.meystre_LNMC\\Cellpose_Training_20211227_v2\\models"))
        .useGPU()
        .build()

// train() will:
// 1. Go through the current project and save all "Training" and "Validation" regions into a
temp folder (inside the current project)
// 2. Run the Cellpose training via command line
// 3. Recover the model file after training, and copy it to where you defined in the builder,
returning the reference to it

def resultModel = CellposeTrainer.train()

println "Model Saved under "+resultModel
```

1. Run CellposeCyto2ModelPrediction.groovy

```
/*
 * Run Cellpose detection demo using CYTO2 model
 * @author Olivier Burri
 * Last tested on QuPath 0.5.1
 *
 * DEPENDENCIES
 * ------------
 * Please follow the installation instructions of the QuPath Extension Cellpose
 * https://github.com/BIOP/qupath-extension-cellpose/blob/main/README.md
 *
 */

def model = 'cyto2'

-- START OF SCRIPT
def cellpose = Cellpose2D.builder( model )
        .pixelSize( 0.3460*2 )
        .tileSize( 2048 )
        .diameter( 30 )
        .measureShape()
        .measureIntensity()
        .createAnnotations()
        .classify( "Labels" )
        .build()


// Run detection for all Annotations
def imageData = getCurrentImageData()
def pathObjects = getAnnotationObjects().findAll{ it.getPathClass() != getPathClass( "Labels"
) }
```

```
cellpose.detectObjects( imageData, pathObjects )
println 'Done!'

import qupath.ext.biop.cellpose.Cellpose2D
```

2. Run Cellpose V1 Model Prediction.groovy

```
/*
 * Run Cellpose detection demo using V1 NISSL model
 * @author Olivier Burri
 * Last tested on QuPath 0.5.1
 *
 * DEPENDENCIES
 * ------------
 * Please follow the installation instructions of the QuPath Extension Cellpose
 * https://github.com/BIOP/qupath-extension-cellpose/blob/main/README.md
 *
 */

def                                          model                                  =
'cellpose_residual_on_style_on_concatenation_off_train_2021_12_13_11_14_32.300178'


// START OF SCRIPT
def pathModel = buildPathInProject( "models", model )
def cellpose = Cellpose2D.builder( pathModel )
        .pixelSize( 0.3460 )
        .tileSize( 2048 )
        .diameter( 30 )
        .measureShape()
        .measureIntensity()
        .createAnnotations()
        .classify( "Labels" )
        .build()


// Run detection for all Annotations
def imageData = getCurrentImageData()
def pathObjects = getAnnotationObjects().findAll{ it.getPathClass() != getPathClass( "Labels"
) }

cellpose.detectObjects( imageData, pathObjects )
println 'Done!'

import qupath.ext.biop.cellpose.Cellpose2D
```

2.Run Cellpose V2 Model Prediction.groovy

```
/*
 * Run Cellpose detection demo using V2 NISSL model
 * @author Olivier Burri
 * Last tested on QuPath 0.5.1
 *
 * DEPENDENCIES
 * ------------
 * Please follow the installation instructions of the QuPath Extension Cellpose
 * https://github.com/BIOP/qupath-extension-cellpose/blob/main/README.md
 *
 */

def                                          model                                  =
'cellpose_residual_on_style_on_concatenation_off_train_2022_01_11_15_43_09.386142'


// START OF SCRIPT
def pathModel = buildPathInProject( "models", model )
def cellpose = Cellpose2D.builder( pathModel )
```

```
        .pixelSize( 0.3460 )
        .tileSize( 2048 )
        .diameter( 30 )
        .measureShape()
        .measureIntensity()
        .createAnnotations()
        .classify( "Labels" )
        .build()


// Run detection for all Annotations
def imageData = getCurrentImageData()
def pathObjects = getAnnotationObjects().findAll{ it.getPathClass() != getPathClass( "Labels"
) }

cellpose.detectObjects( imageData, pathObjects )
println 'Done!'

import qupath.ext.biop.cellpose.Cellpose2D
```
