# Supplementary material for: Cell density quantification of high resolution Nissl images of the juvenile rat brain
Source: Front Neuroanat. 2024 Dec 18;18:1463632. doi: 10.3389/fnana.2024.1463632 (PMC11688196; doi:10.3389/fnana.2024.1463632)
Supplement: Supplementary file 2 [file Data_Sheet_2.pdf]

Supplementary Material

1 SUPPLEMENTARY DATA

2 SUPPLEMENTARY TABLES AND FIGURES

Figures, tables, and images will be published under a Creative Commons CC-BY licence and permission must be obtained for use of copyrighted material from other sources (including re-published/adapted/modified/partial figures and images from the internet). It is the responsibility of the authors to acquire the licenses, to follow any citation instructions requested by third-party rights holders, and cover any supplementary charges.

2.1 Tables

Animal [ID]	Hemisphere [Right or Left]	QuPath project [ID]	Segmentation ground truth [N_{slices}]	Cell density S1HL [N_{slices}]	Layer boundaries ground truth [N_{slices}]	ML predictions S1HL [N_{slices}]
1 (01413827)	Right	01413827_RH_Nissl_1	2	7	2	8
1 (01413827)	Left	01413827_LH_Nissl_2		18	3	22
2 (01413828)	Right	01413828_RH_Nissl_2		12	3	16
2 (01413828)	Left	1413828_LH_Nissl_3	1	19	3	22
3 (01413829)	Right	01413829_RH_Nissl_2		20	3	23
3 (01413829)	Left	N/A: Excluded, wrong slicing orientation				
4 (01443459)	Right	1443459_RH_Nissl_4		17	3	22
4 (01443459)	Left	1443459_LH_Nissl_5				
5 (01443460)	Right	1443460_RH_Nissl_4	1	17	3	21
5 (01443460)	Left	1443460_LH_Nissl_5		16	3	17
6 (01449920)	Right	N/A: Excluded, hydrocephalic hemisphere				
6 (01449920)	Left	1449920_LH_Nissl_6		22	3	26
7 (01449921)	Right	1449921_RH_Nissl_6		19	3	24
7 (01449921)	Left	1449921_LH_Nissl_6		14	3	16
8 (01449922)	Right	1449922_RH_Nissl_6		19	3	22
8 (01449922)	Left	1449922_LH_Nissl_6		18	3	23

Table S1. Sample table organized per hemisphere, detail of the QuPath project name, and the sample size for the cell segmentation ground truth, cell densities calculation (data related to Figure 2, 3, and 4), the layer boundaries ground truth and ML method (data related to Figure 6, 7, and 8).

2.2 Figures

**Features**	**Mean importance [%]**	**STDEV importance [%]**
Smoothed: 50 μm: Distance to annotation with Outside Pia μm	20.47	0.18
Distance to annotation with Outside Pia μm	18.92	0.16
Smoothed: 50 μm: Min diameter μm	1.35	0.04
Centroid Y μm	0.69	0.04
Smoothed: 50 μm: Max diameter μm	0.26	0.05
Centroid X μm	0.16	0.03
Smoothed: 50 μm: Circularity	0.11	0.03
Smoothed: 50 μm: Delaunay: Max triangle area	0.09	0.04
Smoothed: 50 μm: CresylViolet: Std.Dev.	0.07	0.02
Smoothed: 50 μm: Solidity	0.06	0.02
Smoothed: 50 μm: Delaunay: Num neighbors	0.048	0.014
Smoothed: 50 μm: Delaunay: Min distance	0.033	0.017
Length μm	0.024	0.014
Delaunay: Median distance	0.024	0.017
CresylViolet: Std.Dev.	0.014	0.013
Max diameter μm	0.010	0.015
Area μm^2	0.006	0.009
Min diameter μm	0.005	0.013
Delaunay: Mean triangle area	0.002	0.012

Table S2. Table of feature importance with mean and standard deviation values for KNN and RF models trained on separated Layer II and III.

Features	Mean importance [%]	Std importance [%]
Distance to annotation with Outside Pia μm	22.88	0.18
Smoothed: 50 μm: Distance to annotation with Outside Pia μm	19.02	0.15
Smoothed: 50 μm: Min diameter μm	1.22	0.04
Centroid Y μm	1.11	0.06
Smoothed: 50 μm: Max diameter μm	0.21	0.04
Centroid X μm	0.16	0.03
Smoothed: 50 μm: Delaunay: Max triangle area	0.08	0.04
Smoothed: 50 μm: Circularity	0.07	0.03
Smoothed: 50 μm: Solidity	0.07	0.02
Smoothed: 50 μm: Delaunay: Min distance	0.05	0.02
Smoothed: 50 μm: Nearby detection counts	0.02	0.04
Area μm^2	0.02	0.012
Smoothed: 50 μm: CresylViolet: Min	0.02	0.03
Smoothed: 50 μm: Area μm^2	0.01	0.02
Smoothed: 50 μm: Length μm	0.01	0.03
Delaunay: Median distance	0.006	0.018
Smoothed: 50 μm: CresylViolet: Std.Dev.	0.004	0.026
Delaunay: Mean distance	0.001	0.008
Max diameter μm	0.001	0.016

Table S3. Table of feature importance with mean and standard deviation values for KNN and RF models trained on merged Layer II and III.

QuPath project	Layer I	Layer II	Layer III	Layer IV	Layer V	Layer VIa	Layer VIb
1413827_RH_Nissl_1	37606.01	89975.61	83902.81	96126.2	73692.87	86307.57	72330.72
1413827_LH_Nissl_2	41655.85	91863.04	86373.90	102113.79	75414.12	88835.58	64400.69
1413828_RH_Nissl_2	43604.52	88309.35	84324.28	97246.7	71697.34	85763.39	67552.6
1413828_LH_Nissl_3	40055.26	93246.42	85849.10	97562.45	73241.6	85544.53	60274.92
1413829_RH_Nissl_2	39608.70	91082.47	87043.63	103101.79	74001.5	89766.63	77808.71
1443459_RH_Nissl_4	33112.71	77552.67	80572.56	102984.28	70504.84	88973.03	70677.55
1443460_RH_Nissl_4	37055.69	88718.61	82868.25	100815.22	74024.25	88718.95	73250.08
1443460_LH_Nissl_5	38567.98	89669.51	86452.38	101793.26	74018.11	90098.23	75458.75
1449920_LH_Nissl_6	22999.68	100823.65	90768.04	109742.27	70321.09	93505.00	69996.13
1449921_LH_Nissl_6	19791.59	78147.69	74205.91	97781.07	66156.68	82277.75	67497.02
1449922_RH_Nissl_6	26663.05	91364.95	88904.51	107590.69	72721.59	93994.72	78377.5
1449922_LH_Nissl_6	24116.81	89828.59	85046.69	105076.67	69376.93	91461.41	76724.48

Table S4. Cell densities prediction detailed per hemisphere and per layer (unit: cells/mm^3).

	Smaller cell diameter Gaussian mean [μm]	Larger cell diameter Gaussian mean [μm]	Diameter difference p-value	Adjacent layer cell density difference p-value
Layer I	5.35	9.46	1.8e-256	
				1.61e-222
Layer II	6.77	11.05	4.0e-301	
				3.44e-10
Layer III	6.76	11.31	0.0e+00	
				4.32e-80
Layer IV	6.78	10.70	0.0e+00	
				4.29e-177
Layer V	5.92	11.28	0.0e+00	
				1.92e-150
Layer VIa	5.75	10.30	0.0e+00	
				2.31e-49
Layer VIb	5.45	10.14	1.4e-276	

Table S5. Cell population characterization. The first two columns show the lower and higher cell population means for a bimodal Gaussian fit to each layer and image [μm]. The third column shows the p-values for the difference between the two diameter distributions. The last column represents the statistical significance of the differences in the cell density across the adjacent layers.

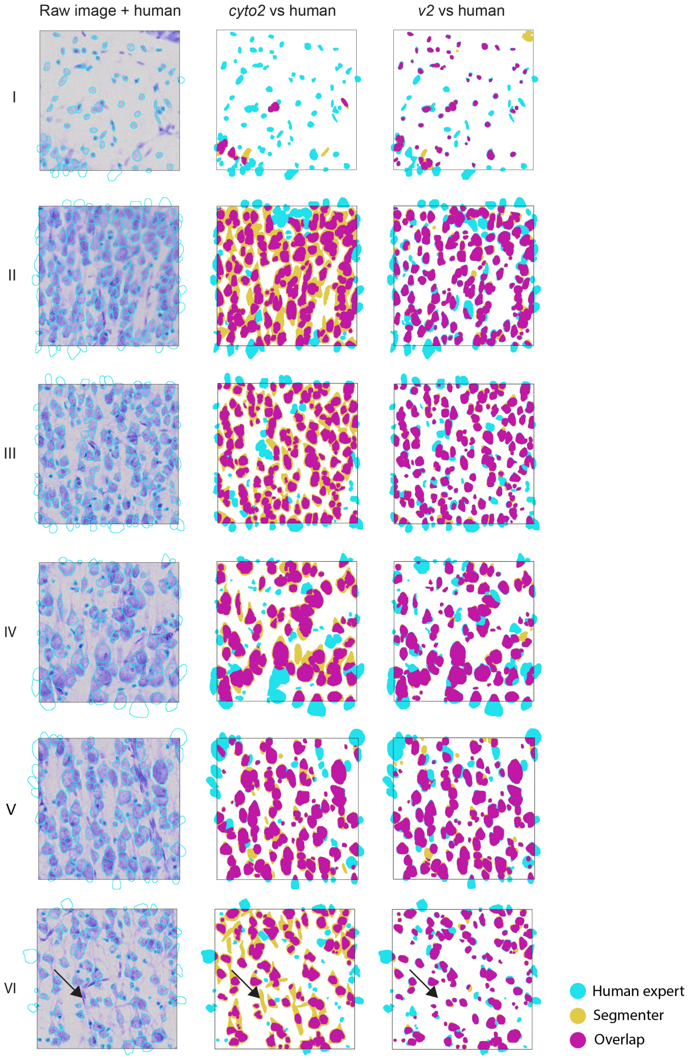

Figure S1. Exemplar of the cell segmentation in each cortical layer with in left column, the human annotation superposed on the raw image; in the middle column the superposition of the segmentation result of $cyto2$ and the human annotation, and in the right column the superposition of the segmentation result of $v2$ with the human annotation. Arrows in Layer VI show a capillary being excluded from the segmentation.

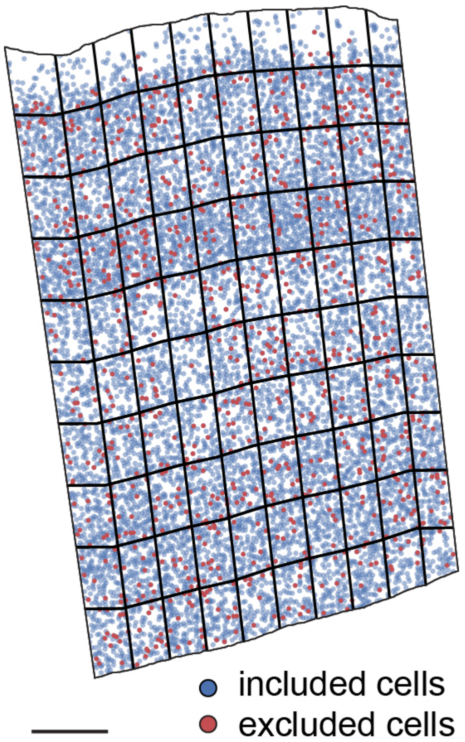

Figure S2. The stereology exclusion overlaid with the cell density calculation grid.

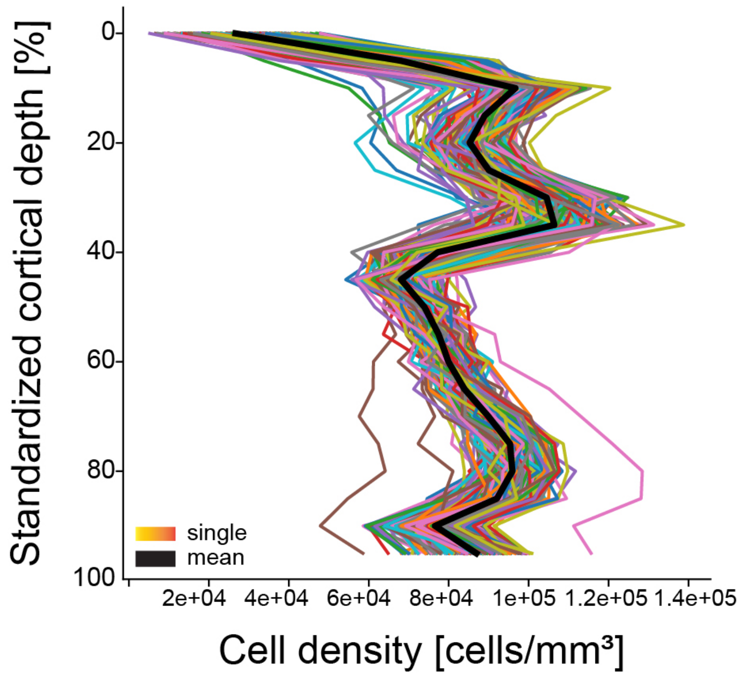

Figure S3. Individual cell densities per standardized cortical depth for Animals 1 to 8 are shown, along with the pooled data. Each trace represents one image.

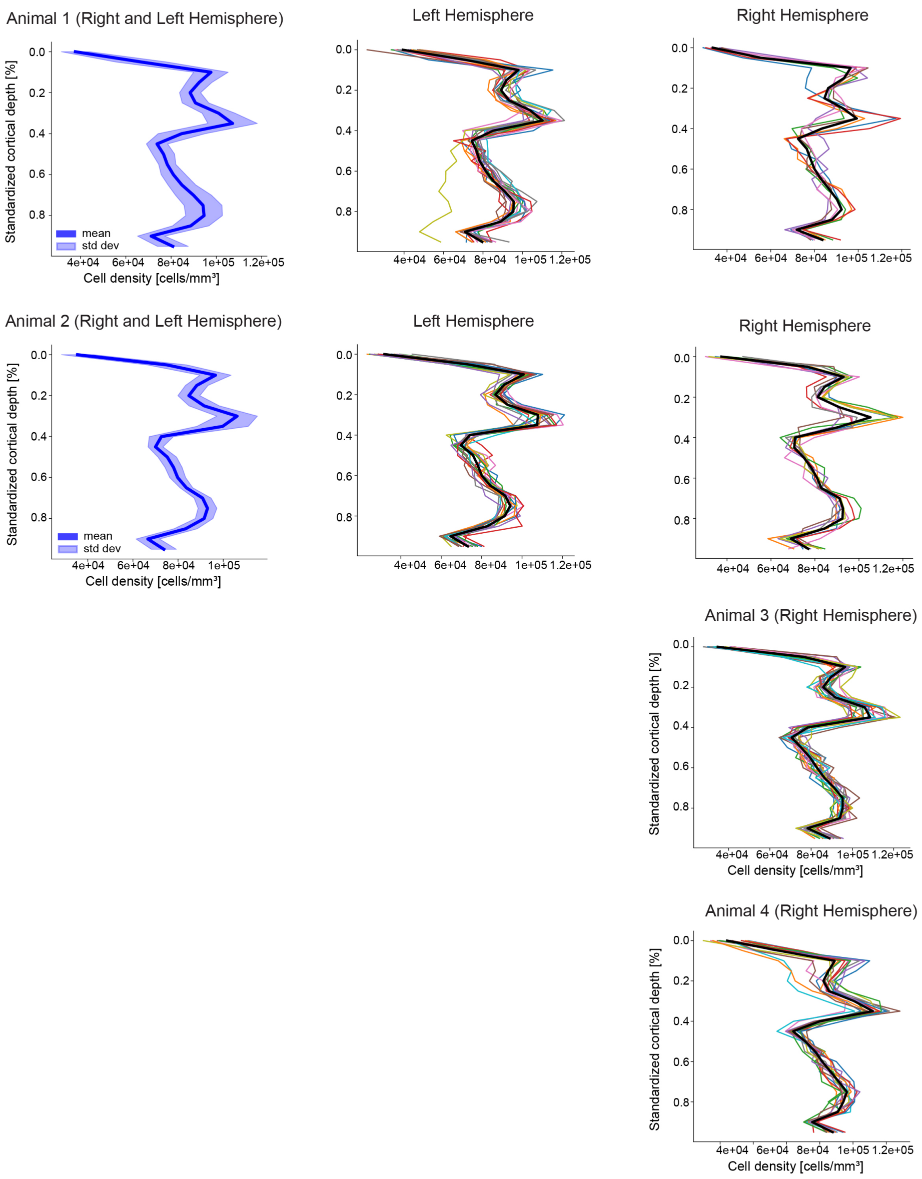

Figure S4. Individual cell densities per standardized cortical depth for hemispheres of Animals 1 to 4 are shown, along with the pooled data one animal per line. If a hemisphere was excluded, the remaining hemisphere results are shown and not the pooled data.

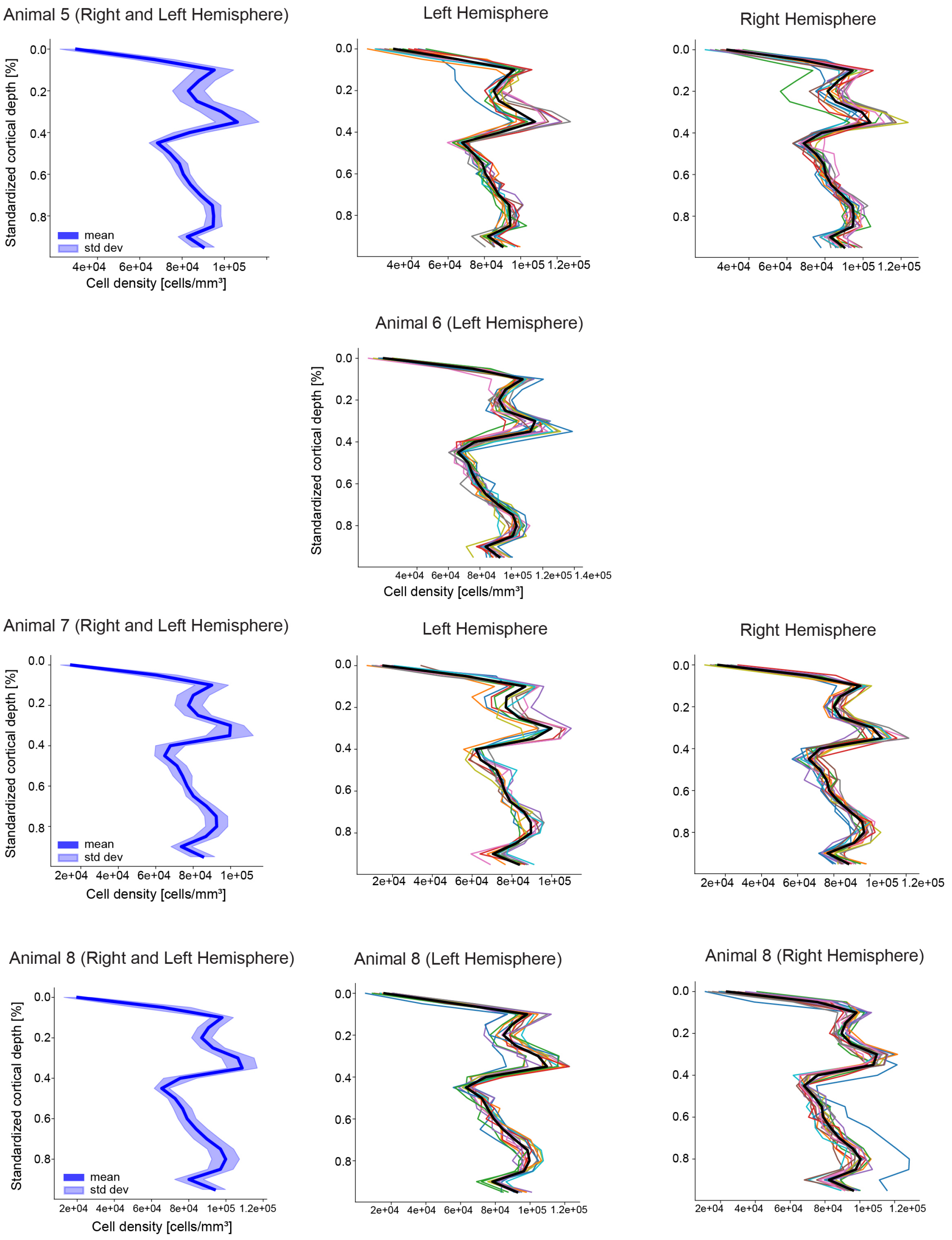

Figure S5. Individual cell densities per standardized cortical depth for hemispheres of Animals 5 to 8 are shown, along with the pooled data per animal, one animal per line. If a hemisphere was excluded, the results for the remaining hemisphere are shown, not the pooled data.

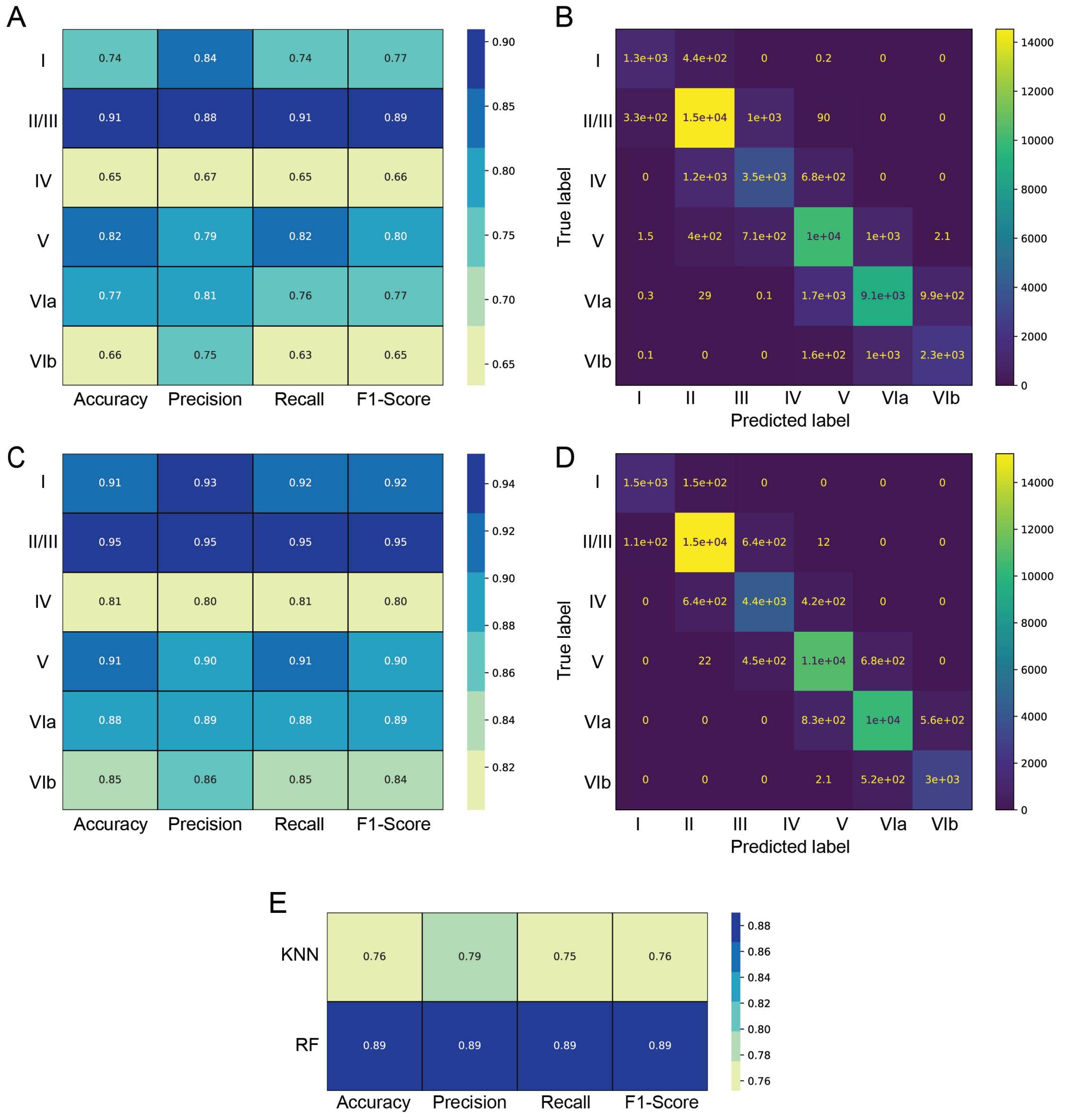

Figure S6. Classification metrics for KNN and RF models on merged Layer II and III. (A): KNN generated classification metrics per layer (B) confusion matrix of the KNN model (C) RF generated classification metrics per layer (D) confusion matrix of the RF model (E) overall per-model metrics.

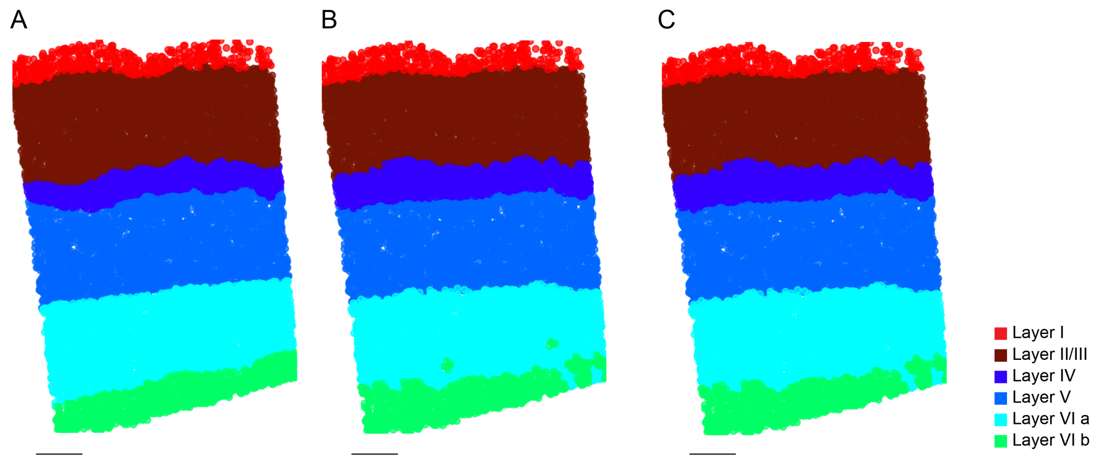

Figure S7. Comparison of predictions with and without post-processing when layer II and III are merged. The overall impact of post processing in terms of metrics is small since few cells are affected. However the predictions appear subjectively cleaner. (A) Ground truth, (B) RF predictions without post processing and (C) RF predictions with post processing.

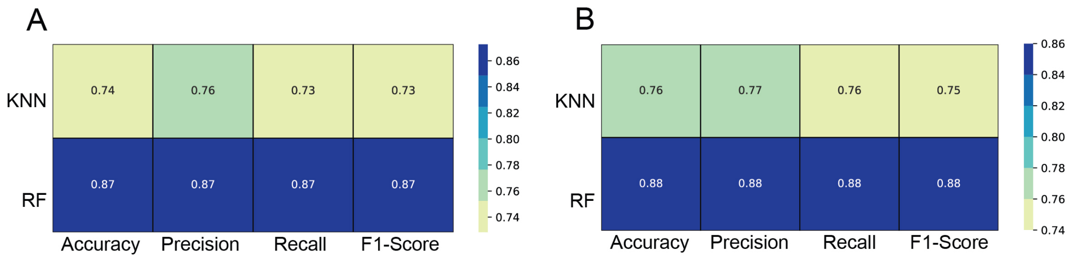

Figure S8. Comparison of the per-class metrics for each model, with in (A): classes with varying sample sizes contribute equally to the final metric values. The values here are simple averages of each per-class metric. And in (B): classes with more samples having a proportionally higher weight in the metrics. The values are weighted averages (proportional to the per-class sample size) of each per-class metric.

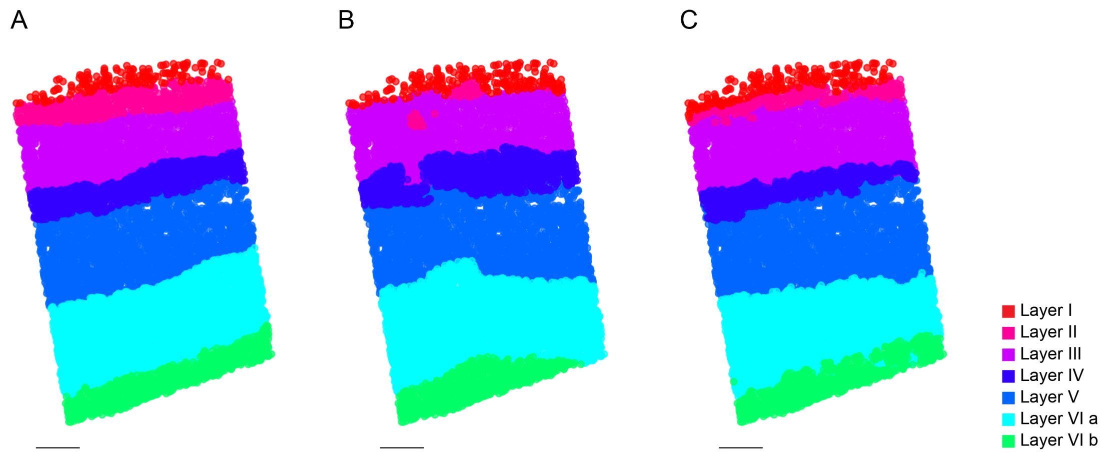

Figure S9. Comparison of the predictions of the KNN model and the RF model with the ground truth. (A) Human expert, (B) KNN predictions, (C) RF prediction. Scale bar: 250 μm.

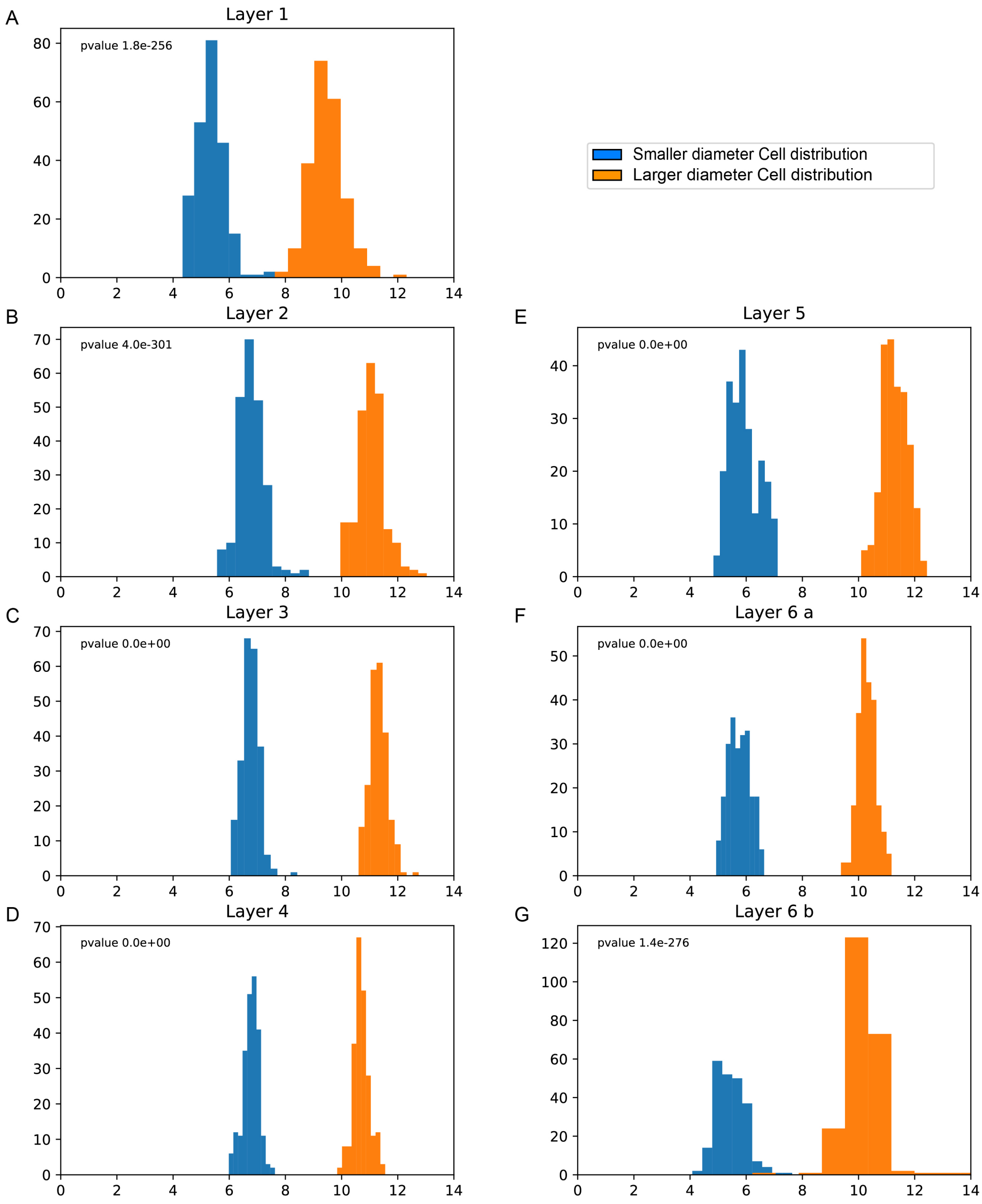

Figure S10. Distributions of the centers of Gaussian distributions fit to cell diameters (unit: μm) in each layer. (A-G) Per layer smaller (blue) and larger (yellow) cell diameter centers. Two-sample t-tests (with p-values inferior to 0.05), were conducted for each layer, between the high and low center populations.

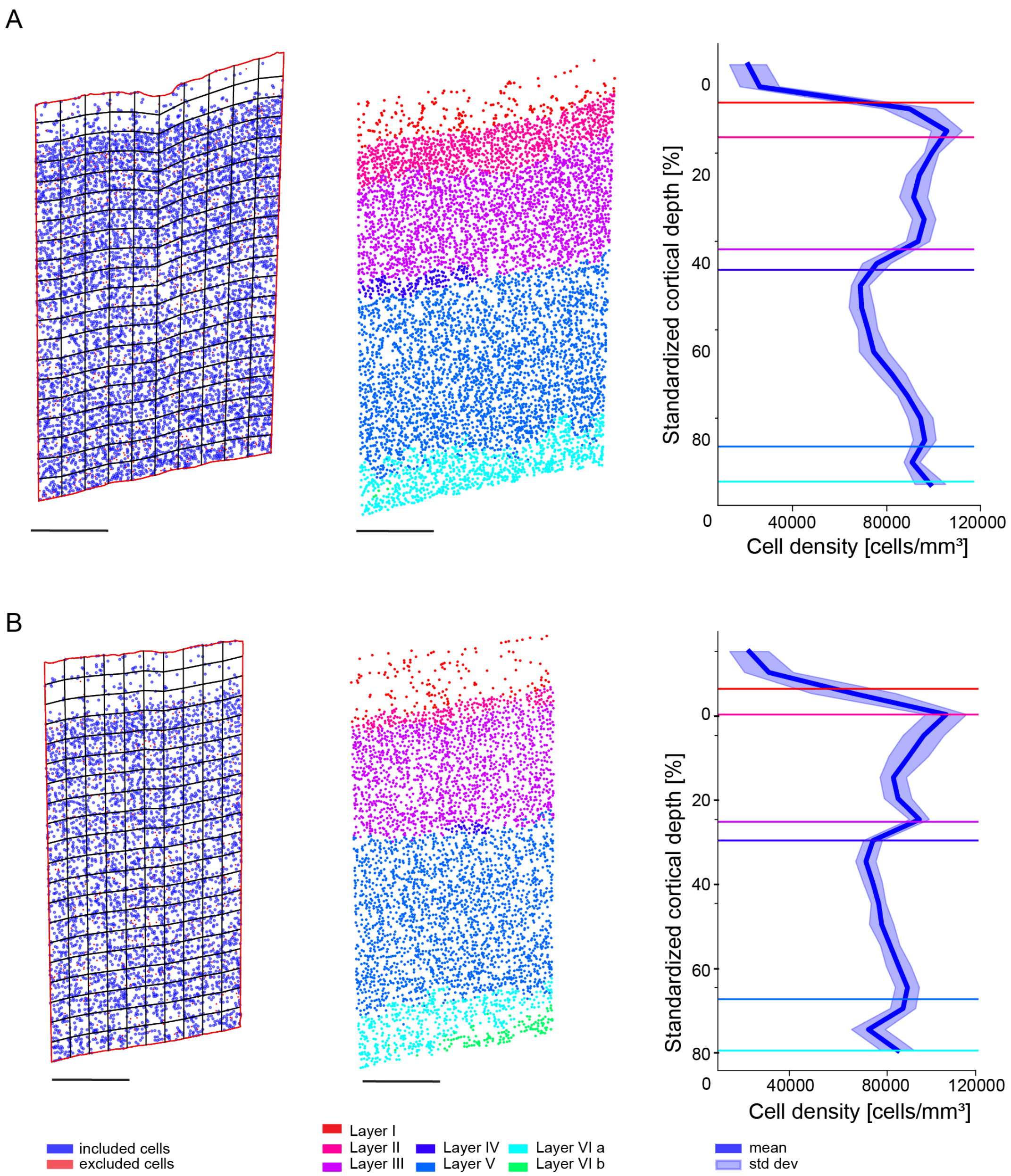

Figure S11. The MPtA brain region test. (A) and (B) from different slices and hemispheres. The left panels show the stereology exclusion overlaid with the cell density calculation grid. The center shows the S1HL-RF model prediction of the layer boundaries. The right panel graphs the cell density per cortical depth with the predicted mean of the layer boundaries superposed (black dotted lines).
